# Metabolomic Profiling and Antioxidant Activities of *Breonadia salicina* Using ^1^H-NMR and UPLC-QTOF-MS Analysis

**DOI:** 10.3390/molecules26216707

**Published:** 2021-11-05

**Authors:** Dorcas B. Tlhapi, Isaiah D. I. Ramaite, Chinedu P. Anokwuru

**Affiliations:** 1 Department of Chemistry, University of Venda, Private Bag X5050, Thohoyandou 0950, South Africa; dorcastlhapi@gmail.com; 2 Department of Pharmaceutical Sciences, Faculty of Science, Tshwane University of Technology, Pretoria 0001, South Africa; anokwuruchi@gmail.com

**Keywords:** chemical profile, phytochemical compositions, *Breonadia salicina*, antioxidant activity

## Abstract

*Breonadia salicina* (Vahl) Hepper and J.R.I. Wood is widely used in South Africa and some other African countries for treatment of various infectious diseases such as diarrhea, fevers, cancer, diabetes and malaria. However, little is known about the active constituents associated with the biological activities. This study is aimed at exploring the metabolomics profile and antioxidant constituents of *B. salicina*. The chemical profiles of the leaf, stem bark and root of *B. salicina* were comprehensively characterized using proton nuclear magnetic resonance (^1^H-NMR) spectroscopy and ultra-performance liquid chromatography with quadrupole time-of-flight mass spectrometry (UPLC-QTOF-MS). The antioxidant activities of the crude extracts, fractions and pure compounds were determined using the DPPH (2,2-diphenyl-1-picrylhydrazyl) free radical scavenging and reducing power assays. A total of 25 compounds were tentatively identified using the UPLC-QTOF-MS. Furthermore, the ^1^H-NMR fingerprint revealed that the different parts of plant had differences and similarities among the different crude extracts and fractions. The crude extracts and fractions of the root, stem bark and leaf showed the presence of α-glucose, β-glucose, glucose and fructose. However, catechin was not found in the stem bark crude extracts but was found in the fractions of the stem bark. Lupeol was present only in the root crude extract and fractions of the stem bark. Furthermore, 5-*O*-caffeoylquinic acid was identified in the methanol leaf extract and its respective fractions, while the crude extracts and fractions from the root and dichloromethane leaf revealed the presence of hexadecane. Column chromatography and preparative thin-layer chromatography were used to isolate kaempferol 3-*O*-(2″-*O*-galloyl)-glucuronide, lupeol, d-galactopyranose, bodinioside Q, 5-*O*-caffeoylquinic acid, sucrose, hexadecane and palmitic acid. The crude methanol stem bark showed the highest antioxidant activity in the DPPH (2,2-diphenyl-1-picrylhydrazyl) free radical scavenging activity with an IC_50_ value of 41.7263 ± 7.6401 μg/mL, whereas the root crude extract had the highest reducing power activity with an IC_0.5_ value of 0.1481 ± 0.1441 μg/mL. Furthermore, the ^1^H-NMR and UPLC-QTOF-MS profiles showed the presence of hydroxycinnamic acids, polyphenols and flavonoids. According to a literature survey, these phytochemicals have been reported to display antioxidant activities. Therefore, the identified hydroxycinnamic acid (caffeic acid), polyphenol (ellagic acid) and flavonoids (catechin and (epi) gallocatechin) significantly contribute to the antioxidant activity of the different parts of plant of *B. salicina*. The results obtained in this study provides information about the phytochemistry and phytochemical compositions of *Breonadia salicina*, confirming that the species is promising in obtaining constituents with medicinal potential primarily antioxidant potential.

## 1. Introduction

*Breonadia salicina* (Vahl) Hepper and J.R.I. Wood is a plant species belonging to the family Rubiaceae. The Rubiaceae family consists of ~13,500 species in ~620 genera and is one of the largest of the angiosperm family. It is divided into four subfamilies: Cinchonoideae, Ixoroideae, Antirheoideae and Rubioideae [1,2]. *Breoanadia salicina* is commonly known as ‘‘Transvaal teak’’ (in English), “mingerhout”, “waterboekenhout” or “basterkiaat” (in Afrikaans), “mutulume” (in Venda) and “matumi” (in Pedi). It is a small to large tree up to 40 m in height [3]. *B. salicina* occurs in the tropical and subtropical regions of Africa and Saudi Arabia. In South Africa, it is widely distributed in the northeast, from KwaZulu-Natal to Mpumalanga and Limpopo near the banks or in the waters of permanent streams and rivers [4]. The secondary metabolite characteristic of the Rubiaceae family includes alkaloids, terpenes, iridoids, quinonic acid glycosides, flavonoids, coumarins, anthraquinones and other phenolic derivatives [5]. These secondary metabolites possess pharmacological activities such as antioxidant, anti-inflammatory, anti-diabetic, antimicrobial, antiplasmodial, antidiarrheal and antitumor [6].

Different parts of *Breonadia salicina* are commonly used by traditional healers in South Africa and other African countries for the treatment of many infectious diseases such as arthritis, pneumonia, tachycardia, vomiting, ulcers, stomach pains, gastrointestinal illness, headaches, inflamed wounds, and bacterial and fungal infections [7,8,9,10]. Despite *B. salicina* being used in traditional medicine, little is known about the phytochemistry and pharmacological activities. Moreover, many of the reports on the biological activities of this plant have been limited to crude extracts. Therefore, there is a need to isolate compounds from different parts of *B. salicina* and evaluate their pharmacological activities which can contribute to human health. Studies on the phytochemistry of *Breonadia salicina* has revealed very few isolated compounds, mainly pentacyclic triterpenoids (ursolic acid and *α*-amyrin [8,11]), hydroxycinnamic acid (2,4-dihydroxycinnamic acid [12]), phytosterol (stigmasterol [11]) and coumarins (7-(*β*-D-apiofuranosyl (1-6)-*β*-D-glucopyranosyl) umbelliferone, 7-hydroxy coumarin and 6- hydroxy-7-methoxy coumarin [11]). According to a literature survey, the biological activities of these compounds isolated from *B. salicina* have never been evaluated. In order to characterize the chemical fingerprints or profile completely; the crude extracts and fractions of the stem bark, root and leaf of *Breonadia salicina* were evaluated using metabolomics approach that coupled the use of proton nuclear magnetic resonance (^1^H-NMR) and UPLC-QTOF-MS. The distribution of antioxidants in different parts of the plant were determined using the DPPH free radical scavenging and reducing power assays. Natural antioxidants play an important role as part of the human diet and for their potential health benefits [13]. Oxidative stress, caused by the accumulation of free radicals and reactive oxygen species (ROS), has been associated with the pathogenesis of many degenerative and chronic conditions such as atherosclerosis, cancer, inflammation, Alzheimer’s, diabetes and inflammation [14]. Antioxidants protect biological molecules (DNA) from oxidation to reduce the risk of developing degenerative and chronic diseases [15]. Studies have revealed that medicinal plants are good sources of antioxidants, because they are rich in phenolic compounds [16]. These compounds can protect humans from high level of free radicals, inhibit lipid peroxidation, scavenge free radicals and chelate metal ions [17].

A previous study proved that the stem bark crude extracts of *B. salicina* has strong antioxidant activity against DPPH free radical scavenging assay [18]. However, the antioxidant activity of the crude root extract from *B. salicina* has never been assessed. Furthermore, there are no reports on the isolation and evaluation of the compounds responsible for the antioxidant activity of this plant. Therefore, this study aimed at determining the phytochemical of different parts of *Breonadia salicina* and linking these results with antioxidant activity using a metabolomics approach to isolate the major compounds and to evaluate their role in the antioxidant activity. Therefore, this study is the first study to detect significant antioxidant activity of different parts of *Breonadia salicina*.

## 2. Results and Discussion

### 2.1. Chemical Fingerprint of the Crude Extracts and Fractions of the Stem Bark, Root and Leaf Using ^1^H-NMR

The *Breonadia salicina* crude extracts and fractions were subjected to ^1^H-NMR analysis, and the chemical shifts were compared to known standards or from literature [19,20,21,22,23,24]. Different classes of identified metabolites such as triterpenoids, fatty acids, sugars (monosaccharides), phenols and quinic acids were identified. This is the first study to identify these metabolites from different parts of *B. salicina*. The chemical shifts of the identified metabolites are presented in Table 1. In the aromatic region of fraction S_1_, proton signals belonging to catechin were detected at δ_H_ 7.05 ppm, δ_H_ 6.72–6.85 ppm, δ_H_ 5.86 ppm and δ_H_ 5.94 ppm, respectively, as shown in Figure S1A. However, fraction S_2_ showed the aromatic proton signals for catechin at δ_H_ 7.06 ppm, δ_H_ 6.72–6.86 ppm, δ_H_ 5.87 ppm and δ_H_ 5.94 ppm, respectively, as presented in Figure S2. However, catechin was not found in the stem bark crude extract but was found in the fractions of the stem bark. This may be because the signals of catechin were not visible in the ^1^H-NMR spectra of the stem bark crude extract or the concentration of catechin was low in the stem bark extract. The signals of lupeol, a pentacyclic triterpenoid, were identified in the root crude extract (R.crude, Figure S4), fraction S_1_ (Figure S1B) and fraction S_2_ (Figure S3). Moreover, signals belonging to 5-*O*-caffeoylquinic acid were detected in the methanol leaf crude extract (LM.crude, Figure S5), fraction LM_2_ (Figure S6) and fraction LM_3_ (Figure S7). The dichloromethane leaf crude extract (LD.crude, Figure S8), fraction R_1_ (Figure S9) and fraction LD_3_ (Figure S10) exhibited proton signals of hexadecane at δ_H_ 0.90 (6H, t)-1.69 (28H, m) ppm, δ_H_ 0.84 (6H, t)-1.27 (28H, m) ppm and δ_H_ 0.87 (6H, t)-1.27 (28H, t) ppm, respectively. Monosaccharides (sugars) such as α-glucose, β-glucose and fructose were observed in the root crude extract (R.crude, Figure S4), stem bark crude extract (S.crude, Figure S11), methanol leaf crude extract (LM.crude, Figure S5) and fraction LM_3_ (Figure S7). The ^1^H-NMR spectra revealed that the crude and fractions of the stem bark, root and leaf contained differences and similarities among the different crude extracts and fractions. The crude extracts and fractions of the root, stem bark and leaf showed the presence of α-glucose, β-glucose, glucose and fructose. However, catechin was not found in the stem bark crude extracts but was found in the fractions of the stem bark. Lupeol was present only in the root crude extract and fractions of the stem bark. Furthermore, 5-*O*-caffeoylquinic acid was identified in the methanol leaf extract and its respective fractions, while the crude extracts and fractions from the dichloromethane leaf revealed the presence of hexadecane.

### 2.2. Identification of Constituents from the Crude Extracts and Fractions of the Stem Bark, Root and Leaf Using UPLC-QTOF-MS Analysis

The identification of the components was also carried out by UPLC-QTOF-MS. A total of twenty-five metabolites from the extracts and fractions of the stem bark, root and leaf of *Breonadia salicina* have been identified and tentatively characterized by comparing their spectral data with values in the literature. UPLC-QTOF-MS data for the identified compounds, namely, their fragmentation ions, retention time, the molecular ion [M−H]^−^ and the main product ions, were provided in Table 2. Peak 1 (Figure S12, *m/z* 377.08633 [M-H]^−^), corresponding to the elemental composition C_18_H_18_O_9_, generated fragments ions at *m/z* 341, 215 and 160. The data for peak 1 are similar to published data of caffeic acid derivative [25]. Peak 2 (Figure S13, *m/z* 461.07350 [M-H]^−^), peak 3 (Figure S14, *m/z* 300.99929 [M-H]^−^), peak 4 (Figure S15, *m/z* 447.05763 [M-H]^−^) and peak 5 (Figure S16, *m/z* 289.07225 [M-H]^−^) were unambiguously confirmed as 4′-*O*-methyellagic acid-3-*O*-α-L-rhamnopyranoside, ellagic acid, ellagic acid-rhamnopyranoside isomer I and catechin, respectively. Furthermore, peaks 2–5 showed fragment ions at *m/z* 315, 299; *m/z* 242, 174; *m/z* 300.99898 and *m/z* 245.08155, respectively [26,27,28]. However, peak 6 (Figure S17, *m/z* 485.32825 [M-H]^−^), peak 7 (Figure S18, *m/z* 458.33667 [M-H]^−^), peak 8 (Figure S19, *m/z* 499.34385 [M-H]^−^), peak 9 (Figure S20, *m/z* 453.33834 [M-H]^−^) and peak 10 (Figure S21, *m/z* 499.37155 [M-H]^−^) were identified as hydroxyglycyrrhetinic acid (C_30_H_46_O_5_), neotigogenin acetate (C_29_H_46_O_4_), 25-hydroxy-3-epi-dehydrotumulosic acid (C_31_H_48_O_5_), micromeric acid (C_30_H_46_O_3_) and 3-acetylursolic acid (C_32_H_50_O_4_), respectively. The MS-MS spectrum of peaks 6, 7, 8 and 10 yielded fragment ions at *m/z* 485, 441; *m/z* 503, 457; *m/z* 455.35408; and *m/z* 497.36498, respectively. A data comparison with the literature confirmed the identification of these compounds [29,30,31,32,33]. Peak 11 (Rt = 1.876 min), peak 12 (Rt = 4.728 min) and peak 13 (Rt = 11.530 min) were assigned to be (epi) gallocatechin (Figure S22, *m/z* 305.06698 [M-H]^−^), 4-*O*-methylgallic acid (Figure S23, *m/z* 183.02975 [M-H]^−^) and myricetin 3-*O*-glucoside (Figure S24, *m/z* 479.08529 [M-H]^−^), respectively. The MS-MS spectrum of peaks 12 and 13 revealed fragment ions at *m/z* 184.03304 and *m/z* 480.08721, respectively [34,35,36]. In comparison with literature, peak 14 (Figure S25, *m/z* 455.35397 [M-H]^−^) and peak 15 (Figure S26, *m/z* 487.33699 [M-H]^−^) were identified as ursolic acid (C_30_H_48_O_3_) and asiatic acid (C_30_H_48_O_5_), respectively, whereas peak 15 showed a fragment ion at *m/z* 485.32825 [37,38]. Furthermore, comparison with literature data led to the unequivocal identification of peak 16 (Rt = 12.569, C_19_H_14_O_12_) as ellagic acid pentoside (Figure S27, *m/z* 433.04194 [M-H]^−^) and peak 17 (Rt = 1.140, C_7_H_6_O_5_) as gallic acid (Figure S28, *m/z* 170.01742 [M-H]^−^) [27,39]. Peak 18 had a retention time of 0.663 min and exhibited an [M-H]^−^ ion at *m/z* 533.17341 (Figure S29), corresponding to the elemental composition C_19_H_34_O_17_ [40]. However, peak 19 (Figure S30, *m/z* 353.08839 [M-H]^−^) was assigned to chlorogenic acid due to the presence of characteristic product ion at *m/z* 191.05585 [quinic acid-H]^−^ [41]. Moreover, peak 20 (Rt = 8.421 min), 21 (Rt = 10.819 min), 22 (Rt = 10.181 min), 23 (Rt = 12.918 min), 24 (Rt = 14.242 min) and 25 (Rt = 0.671min) with [M-H]^−^ ions at *m/z* 389.10941 (Figure S31), *m/z* 367.10424 (Figure S32), *m/z* 451.10412 (Figure S33), *m/z* 609.14777 (Figure S34), *m/z* 515.12070 (Figure S35) and *m/z* 191.05581 (Figure S36) were tentatively identified as deacetyl asperuloside acid (C_16_H_22_O_11_), 5-methyl caffeoylquinic acid (C_17_H_20_O_9_), cinchonain I isomer (C_24_H_20_O_9_), rutin (C_27_H_30_O_16_), di-*O*-caffeoylquinic acid (C_25_H_24_O_12_) and quinic acid (C_7_H_12_O_6_), respectively. Peaks 20–24 generated fragments ions at *m/z* 390.11317; *m/z* 174.95588; *m/z* 341.06822; *m/z* 463, 447; and *m/z* 353.08946 [26,42,43,44,45], respectively. Finally, a data comparison with the literature confirmed the identification of these compounds. Therefore, metabolites including polyphenols, flavanoids, hydrolyzable tannin, triterpenoids, hydroxycinnamic acids and quinic acids were tentatively identified and characterized from *Breonadia salicina*. Many of these metabolites were mostly found in the stem bark than in the root or leaf samples. Furthermore, this is the first study to identify and report these metabolites from *Breonadia salicina.*

### 2.3. Isolation and Identification of Chemical Constituents

Kaempferol 3-*O*-(2″-*O*-galloyl)-glucuronide (**1**) showed a [M-H]^−^ ion peak at *m/z* 613.08129 matched with the molecular formula of C_28_H_22_O_16_. The ^1^H-NMR spectrum showed six aromatic protons signals at δ_H_ 8.25 ppm (2H, d, H-2′, H-6′), δ_H_ 6.70 ppm (2H, d, H-3′, H-5′), δ_H_ 6.39 ppm (1H, d, H-6), and δ_H_ 6.25 ppm (1H, d, H-8), respectively. Total assignment was done by a close examination of the 1D-NMR (^1^H-NMR and ^13^C-NMR), HRMS and literature data.

The ^13^C-NMR spectrum of lupeol (**2**) revealed the presence of 30 carbon atom signals. Furthermore, the carbon atoms at δc 108.7 ppm and δc 150.5 ppm assigned to carbons at positions 29 and 20, respectively, has showed the presence of C=C group. Moreover, the ^13^C-NMR spectrum displayed that the carbon signal at δc 78.2 ppm has been attributed to C-3. The ^1^H-NMR spectrum revealed the presence of seven tertiary methyl protons at δ_H_ 0.74 ppm (3H, s, 24-H), δ_H_ 0.77 ppm (3H, s, 28-H), δ_H_ 0.87 ppm (3H, s, 25-H), δ_H_ 0.96 ppm (3H, s, 27-H), δ_H_ 0.98 ppm (3H, s, 23-H), δ_H_ 1.02 ppm (3H, s, 26-H) and δ_H_ 1.71 ppm (3H, s, 30-H). A multiplet of one proton at δ_H_ 2.23 ppm (1H, m) has been assigned to 19-H while 3-H proton displayed a multiplet at δ_H_ 3.02 ppm (1H, m). A pair of broad singlets at δ_H_ 4.72 ppm (1H, s) and δ_H_ 4.61 ppm (1H, s) was indicative of olefinic protons at (29a-H and 29b-H). This supported the double bond between methylene carbon (C-29) and quaternary carbon (C-20). Finally, data comparison with the literature confirmed the isolation of lupeol, a pentacyclic triterpenoid.

d-Galactopyranose (**3**), a monosaccharide sugar showed a [M-H]^−^ ion peak at *m/z* 179.0557 matched with the molecular formula of C_6_H_12_O_6_. The ^1^H-NMR spectrum showed the anomers proton at δ_H_ 5.16 ppm (H-1α), δ_H_ 4.52 ppm (H-1β) and δ_H_ 3.32 ppm (H-3β) give rise to peaks positioned outside this envelope of ring protons atom signals between δ_H_ 4.52 ppm (H-1β) and δ_H_ 5.16 ppm (H-1α). According to a literature report, the α anomeric proton atom signals of d-sugars are mainly found between δ_H_ 4.9 ppm and δ_H_ 5.5 ppm, whilst the β proton atom signals are mostly found between δ_H_ 4.3 ppm and δ_H_ 4.7 ppm [46]. A spectroscopic data comparison with the literature confirmed the isolation of D-galactopyranose.

Bodinioside Q (**4**) showed a [M-H]^−^ ion peak at *m/z* 487.3448 [M-Glc-Rha-Xyl-H]^−^ matched with the molecular formula of C_47_H_76_O_18_. The ^1^H-NMR spectrum displayed six methyl signals at δ_H_ 1.14 ppm (3H, s), δ_H_ 1.19 ppm (3H, s), δ_H_ 1.04 ppm (3H, s), δ_H_ 0.95 ppm (3H, s), δ_H_ 0.82 ppm (3H, s) and δ_H_ 0.82 ppm (3H, s). Moreover, the *J*_H1, H2_ coupling constants of two anomeric proton signals at δ_H_ 5.12 ppm (1H, d, H-1′), proposed that the β anomeric configuration for the xylopyranosyl. Furthermore, the HSQC spectrum showed correlation with carbons at δ_C_ 17.4 ppm (C-26), δ_C_ 17.6 ppm (C-25), δ_C_ 13.0 ppm (C-24), and δ_C_ 23.0 ppm (C-30) with protons at assigned to δ_H_ 1.14 ppm (26-H), δ_H_ 1.04 ppm (25-H), δ_H_ 0.95 ppm (24-H), and δ_H_ 0.86 ppm (30-H), respectively. In addition, the HMBC spectrum showed the signal at δ_H_ 5.27 ppm (1H, br. s), assigned to the carbon at δ_C_ 122.0 ppm (C-12) which coupled with δ_C_ 144.1 ppm (C-13), indicated the presence of a double bond. The HMBC spectrum also showed correlations from the anomeric carbon signal at position C-1′ (δ_C_ 107.8 ppm) with 2′-H (δ_H_ 3.80 ppm), and 5′-H (δ_H_ 4.62 ppm), suggested the presence of D-xylopyranose.

5-*O*-Caffeoylquinic acid (**5**) showed the presence of 16 carbons, including two carbonyl groups at δ_C_ 145.4 ppm and δ_C_ 166.5 ppm, corresponding to carbons at positions 7 and 9′, respectively, on the ^13^C-NMR spectrum. Furthermore, the ^13^C-NMR spectrum presented two aromatic carbons bonded to hydroxyl groups at δ_C_ 148.5 ppm and δ_C_ 145.8 ppm corresponding to carbons at positions 4′ and 3′, and two olefinic carbons at δ_C_ 180.2 ppm and δ_C_ 113.6 ppm corresponding to carbons at positions 7 and 8′. The ^1^H-NMR spectrum showed two ortho-coupled doublets at δ_H_ 6.79 ppm and δ_H_ 6.95 ppm corresponding to protons at positions 5′ and 6′. A broad singlet at δ_H_ 7.06 ppm has been assigned to a proton at position 2′, confirming the presence of a tri-substituted aromatic ring. Moreover, the ^1^H-NMR revealed two doublets at corresponding to protons at δ_H_ 6.21 ppm and δ_H_ 7.52 ppm positions 7′ and 8′, indicating the presence of trans-di-substituted ethylene moiety in the compound. These assignments are in good agreement with the structure of 5-*O*-caffeoylquinic acid.

Sucrose (**6**) displayed a molecular peak at *m/z* 341.1073, which is in agreement with the founded molecular formula of C_12_H_22_O_11_. The COSY spectrum showed coupling between protons H-3 (δ_H_ 3.72 ppm) with H-2 (δ_H_ 3.50 ppm) and H-4 (δ_H_ 3.39 ppm), H-2 (δ_H_ 3.50 ppm) and H-1 (δ_H_ 5.41 ppm), respectively. The only coupling constant value released for the protons of the fructoside ring was between H-4′ (δ_H_ 4.02 ppm) with H-5′ (δ_H_ 3.86 ppm) and H-6′ (δ_H_ 3.80 ppm). Furthermore, the HMBC spectrum revealed correlations between H-1′ (δ_H_ 3.63 ppm) with C-5′ (δ_C_ 81.6 ppm), H-4′ (δ_H_ 4.02 ppm) with C-6′ (δ_C_ 62.5 ppm), H-1 (δ_H_ 5.41 ppm) with C-3 (δ_C_ 72.2 ppm), H-5′ (δ_H_ 3.86 ppm) with C-2′ (δ_C_ 103.8 ppm), respectively. Moreover, the glucopyranosyl H-1′ (δ_H_ 3.63 ppm) gave a strong correlation to fructosyl C-2 (δ_C_ 70.2 ppm) establishing the linkage between the anomeric carbon of the glucopyranosyl residue and that of the fructoside one. Total assignment was done by a close examination of the 1D-NMR (^1^H-NMR and ^13^C-NMR), 2D- NMR (COSY and HMBC), HRMS and literature data.

Hexadecane (**7**) showed a triplet at δ_H_ 0.89 ppm (6H, t) corresponding to protons at position 1 and 16 on the ^1^H-NMR spectrum. The ^1^H-NMR spectrum exhibited a long peak of multiplet at δ_H_ 1.28 ppm corresponding to protons assigned to 2-H, 3-H, 4-H, 5-H, 6-H, 7-H, 8-H, 9-H, 10-H, 11-H, 12-H, 13-H, 14-H and 15-H of the long chain, was evident as a multiplet that integrated for one proton. A single long peak multiplet at δ_C_ 29.3–29.7 ppm appeared from C-4, C-5, C-6, C-7, C-8, C-9, C-10, C-11, C-12 and C-13 on the ^13^C-NMR spectra. Furthermore, hexadecane (**7**) was isolated as white crystals with a melting point of 16–18 °C. Therefore, the spectroscopic data and physical property comparison with the literature confirmed the isolation of hexadecane.

The infrared (IR) spectrum of palmitic acid (**8**) revealed absorptions at 3436.00 cm^−1^ which is characteristic of O-H stretching, 2849.51 cm^−1^ which is due to aliphatics (CH_3_) stretching, 1703.71 cm^−1^ due to carbonyl (C=O) stretching and 2917.27 cm^−1^ due to overtone of the long chain (CH_2_)n bending frequency. The ^1^H-NMR spectrum showed a long peak of multiplet at δ_H_ 1.23 ppm assigned to protons at position 4-H, 5-H, 6-H, 7-H, 8-H, 9-H, 10-H, 11-H, 12-H and 13-H of the long chain, was evident as a multiplet that integrated for one proton. Furthermore, the ^1^H-NMR spectrum displayed a triplet at δ_H_ 2.27 ppm (2H, t) and δ_H_ 0.83 ppm (3H, t) corresponding to protons at positions 2 and 16, respectively. The ^13^C-NMR has shown double bonds of the acidic group (–COOH) at δ_C_ 178.4 ppm assigned to C-1 as singlet, the alkane carbon C-16 appeared at δ_C_ 14.1 ppm as a triplet. A single long peak multiplet at δ_C_ 29.0 ppm appeared from C-4 to C-13, whereas an alpha and beta carbon to the acidic group (-COOH) appeared at δ_C_ 31.9 ppm and δ_C_ 34.0 ppm corresponding to carbons at position 3 and 2 as doublets respectively. Furthermore, an alpha and beta carbon to the alkane carbon appeared as doublets at δ_C_ 22.6 ppm and δ_C_ 24.7 ppm corresponding to carbons at position 15 and 14.

### 2.4. Antioxidant Activity

The crude methanol stem bark (S.crude) showed the highest antioxidant activity in the DPPH (2,2-diphenyl-1-picrylhydrazyl) free radical scavenging activity with an IC_50_ value of 41.7263 ± 7.6401 μg/mL, whereas the root crude extract (R.crude) had the highest reducing power activity with an IC_0.5_ value of 0.1481 ± 0.1441 μg/mL, as shown in Table 3. The presence of the identified hydroxycinnamic acids, polyphenols and flavonoids might have contributed to the potent antioxidant activities in the stem bark and root extracts, as shown in Table 1 and Table 2. Kilic et al. (2014) reported that ellagic acid, tentatively identified from the stem bark crude extract (S.crude, as shown in Table 2), exhibited a high DPPH radical scavenging activity of 85.6% at 30 μg/mL [47]. However, Grzesik et al. (2018) indicated that catechin, (epi) gallocatechin and caffeic acid found in the stem bark crude extract (S.crude, as presented in Table 2) have good antioxidant activities against DPPH radical scavenging and reducing power activities with IC_50_ values of 3.965 ± 0.067 mol TE/mol, 2.939 ± 0.037 mol TE/mol and 0.965 ± 0.015 mol TE/mol, respectively, and IC_0.5_ values of 0.793 ± 0.004 mol TE/mol, 1.032 ± 0.007 mol TE/mol and 1.018 ± 0.004 mol TE/mol, respectively [48]. Therefore, ellagic acid, catechin, (epi) gallocatechin and caffeic acid play an important role in the antioxidant activities of the stem bark crude extract (S.crude). Furthermore, d-galactopyranose (**3**) exhibited the highest antioxidant activity against DPPH free radical scavenging activity compared to the other isolated compounds, with an IC_50_ value of 44.5613 ± 2.6772 μg/mL, whereas kaempferol 3-*O*-(2″-*O*-galloyl)-glucuronide (**1**) exhibited the highest reducing power activity compared to the other isolated compounds, with an IC_0.5_ value of 3.3742 ± 1.7492 μg/mL, as shown in Table 3. Moreover, d-galactopyranose (**3**) was less active than the parent fraction S_4_, as shown in Table 3. This could be because the interaction of d-galactopyranose (**3**) with other constituents from the fraction it was isolated from could be responsible for the higher antioxidant activity observed in the DPPH free radical scavenging and reducing power. According to a literature survey, the antioxidant activities of d-galactopyranose (**3**) have never been evaluated. Therefore, our study is the first to detect important antioxidant activity of the crude extracts, fractions and pure compounds from *Breonadia salicina.*

## 3. Materials and Methods

### 3.1. General Experimental Procedure

All chemicals used were analytical grade purchased from Sigma-Aldrich (Darmstadt, Germany). Silica gel (Kieselgel 60 (0.063–0.2 mm), Merck) was used as stationary phase and solvent mixtures described below were used as mobile phase in the chromatographic separations. Thin-layer chromatography (TLC) plates packed with silica gel (normal phase) were used to locate the major constituents of the fractions.

#### 3.1.1. One- and Two-Dimensional Nuclear Magnetic Resonance (NMR) Spectroscopy

One-dimensional (1D) ^1^H-NMR and ^13^C-NMR nuclear magnetic resonance (NMR) spectra were recorded at 400 MHz and 100 MHz, respectively, with an Avance 400 spectrometer (Bruker, Fällanden, Switzerland). The ^1^H-NMR and ^13^C-NMR chemical shifts were recorded in parts per million (ppm). Deuterated (methanol) CD_3_OD, (dimethyl sulfoxide-d_6_) DMSO-d_6_ and (chloroform) CDCl_3_ were used as solvents for preparation of the NMR samples. Two-dimensional (2D) experiments performed were homonuclear correlation spectroscopy (COSY), heteronuclear single quantum correlation (HSQC) and heteronuclear multiple bond correlation (HMBC).

#### 3.1.2. High-Resolution Mass Spectrometry

A Waters Synapt G2 Quadrupole time-of-flight (QTOF) mass spectrometer (MS) (Thermo Fisher Scientific, Waltham, MA, USA) connected to a Waters Acquity ultra-performance liquid chromatograph (UPLC) (Waters, Milford, MA, USA) was used for direct injection high-resolution mass spectrometric analysis. A volume of one μL of sample was injected into a stream of 60% acetonitrile and 40% dilute (0.1%) aqueous formic acid. This conveyed the sample directly to the QTOF mass spectrometer where data were acquired using both positive and negative electrospray ionization. The following MS settings were used: cone voltage of 15 V, desolvation temperature of 275 °C, desolvation gas at 650 L/h, and the rest of the MS settings optimized for best resolution and sensitivity.

#### 3.1.3. UPLC Analysis

The ultra-performance liquid chromatography MS (UPLC-MS) was carried out using a Waters Synapt G2 Quadrupole time-of-flight (QTOF) mass spectrometer (MS) (Thermo Fisher Scientific, Waltham, MA, USA) connected to a Waters Acquity ultra-performance liquid chromatograph (UPLC) (Waters, Milford, MA, USA) and a photodiode array detector (PDA) (Waters, Milford, MA, USA). An injection volume of 2.0 μL (full-loop injection) was used. Separation was achieved on an Acquity UPLC BEH C18 column (150 mm X 2.1 mm i.d., 1.7 μm particle size; Waters, Milford, MA, USA), maintained at 40 °C. The mobile phase consisted of 0.1% formic acid (Solvent A) and HPLC grade (Merck, Darmstadt, Germany) acetonitrile (Solvent B) at a flow rate of 0.3 mL/min. Gradient elution was executed as follows: the initial ratio was 10% B for 4 min, changed to 50% B for 6 min, then 95% B in 2.5 min, maintaining for 0.5 min, before returning to the initial ratio in 0.5 min. The system was equilibrated for 2 min prior to the subsequent analysis. Both positive and negative electrospray ionization (ESI) modes were evaluated for further analysis.

#### 3.1.4. FTIR Spectral Analysis of the Isolated Compounds

Attenuated total reflection (ATR) infrared (IR) spectra were recorded on an Alpha Fourier transform infrared (FTIR) spectrometer (Bruker, Fällanden, Switzerland).

### 3.2. Sampling and Extraction

**Scheme 1:**
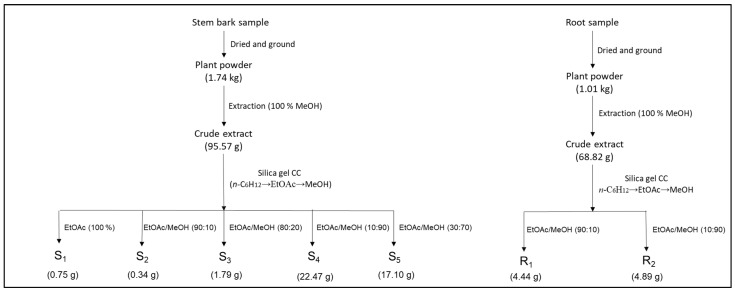
Isolation of fractions from the stem bark and root samples using column chromatography.

The leaves, stem bark and root samples of *Breonadia salicina* were collected at Fondwe, located at latitude 22°55′31.9″ South and longitude 30°15′45.0″ East in the Limpopo Province, South Africa, in October 2019. Samples were identified by Prof. P. Tshisikhawe of the Department of Botany, University of Venda. A voucher number BD 02 was assigned and the voucher was deposited in the herbarium of the Department of Botany. The stem bark, root and leaves were air-dried for four weeks and then ground to a fine powder using an industrial grinding mill (NETZSCH, Selb, Germany). Approximately 1.74 kg ground stem bark and 1.01 kg root of *Breonadia salicina* were each soaked with 2 L of methanol for 48 h at room temperature, respectively. The extracts were filtered and then concentrated using a rotary evaporator (BÜCHI Labortechnik AG, Flawil, Switzerland) at 45 °C to obtain 95.57 g and 68.82 g of dried extracts, respectively (as shown in Scheme 1).

**Scheme 2:**
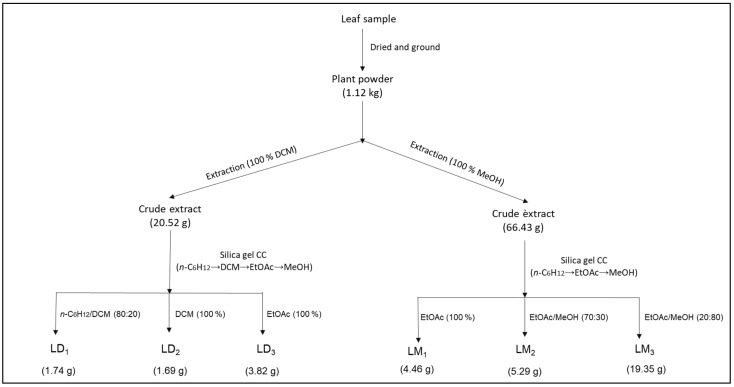
Isolation of fractions from the leaf samples using column chromatography.

Moreover, ~1.12 kg ground leaves of *Breonadia salicina* were extracted successively with 2 L of dichloromethane and methanol for 48 hours at room temperature, respectively. The leaf extracts were filtered and then concentrated using a rotary evaporator (BÜCHI Labortechnik AG, Flawil, Switzerland) at 45 °C to obtain 20.52 g and 66.43 g of dried extracts, respectively (as shown in Scheme 2). Each dried extract (stem bark, root and leaves) was subjected to column chromatography over silica gel (Kieselgel 60 (0.063–0.2 mm), Merck) [49]. The column was eluted initially with hexane and the polarity was gradually increased with ethyl acetate and finally methanol.

### 3.3. Fractionation

A portion of the crude methanol stem bark extract (75.04 g) was dissolved in methanol and adsorbed on silica gel (199.82 g). The dried sample (269.38 g) was loaded onto a silica gel (200.99 g, Kieselgel 60, Merck) column (65 cm × 4.0 cm), slurry packed in hexane/ethyl acetate (50:50). Therefore, the stem bark yielded five fractions coded as S_1_–S_5_. Fraction S_1_ was obtained with ethyl acetate (100%), yielded 0.75 g; S_2_ was obtained with ethyl acetate/methanol (90:10), yielded 0.34 g; S_3_ was obtained with ethyl acetate/methanol (80:20), yielded 1.79 g; S_4_ was obtained with ethyl acetate/methanol (10:90), yielded 22.47 g; and S_5_ was obtained with ethyl acetate/methanol (30/70), yielded 17.10 g (as shown in Scheme 1). Furthermore, a portion of the crude methanol root extract (50.69 g) was dissolved in methanol and adsorbed on silica gel (215.42 g). The dried sample (270.28) was loaded onto a silica gel (200.19 g, Kieselgel 60 (0.063–0.2 mm), Merck) column (65 cm × 4.0 cm), slurry packed in hexane/ethyl acetate (50:50). As a result, two fractions were obtained from the root coded as R_1_–R_2_. Fraction R_1_ was obtained with ethyl acetate/methanol (90:10), yielded 4.44 g, and R_2_ was obtained with ethyl acetate/methanol (10:90), yielded 4.89 g (as shown in Scheme 1). Moreover, a portion of the crude dichloromethane leaf extract (15.52 g) was dissolved in methanol and adsorbed on silica gel (269.12 g). The dried sample (292.07 g) was loaded onto a silica gel (309.67 g, Kieselgel 60 (0.063–0.2 mm), Merck) column (65 cm × 4.0 cm), slurry packed in hexane/dichloromethane (50:50). Consequently, three fractions were obtained from the dicholoromethane leaf extract coded as LD_1_–LD_3_. LD_1_ was obtained with hexane/dicholoromethane (80:20), yielded 1.74 g; LD_2_ was obtained with dicholoromethane (100%), yielded 1.69 g; and LD_3_ was obtained with ethyl acetate (100%), yielded 3.82 g (as shown in Scheme 2). Finally, a portion of the crude methanol leaf extract (50.37 g) was dissolved in methanol and adsorbed on silica gel (162.17 g). The dried sample (203.61 g) was loaded onto a silica gel (301.00 g, Kieselgel 60 (0.063–0.2 mm), Merck) column (65 cm × 4.0 cm), slurry packed in hexane/ethyl acetate (50:50). Thus, three fractions coded as LM_1_–LM_3_ were obtained from the leaf extract. Fraction LM_1_ was obtained with ethyl acetate (100%), yielded 4.46 g; LM_2_ was obtained with ethyl acetate/methanol (70:30), yielded 5.29 g; and LM_3_ was obtained with ethyl acetate/methanol (20:80), yielded 19.35 g (as shown in Scheme 2). The collected fractions from each crude extract were monitored by TLC. The thin-layer chromatograms were developed in a solvent system of chloroform/ethyl acetate/formic acid (CEF 32:4:4). A natural product reagent (1 g methanolic diphenylboric dissolved in 100 mL methanol, combined with 5 mL PEG 400 dissolved in 95 mL ethanol) was used to visualize compounds on a TLC.

### 3.4. Purification of Fractions

**Figure 1:**
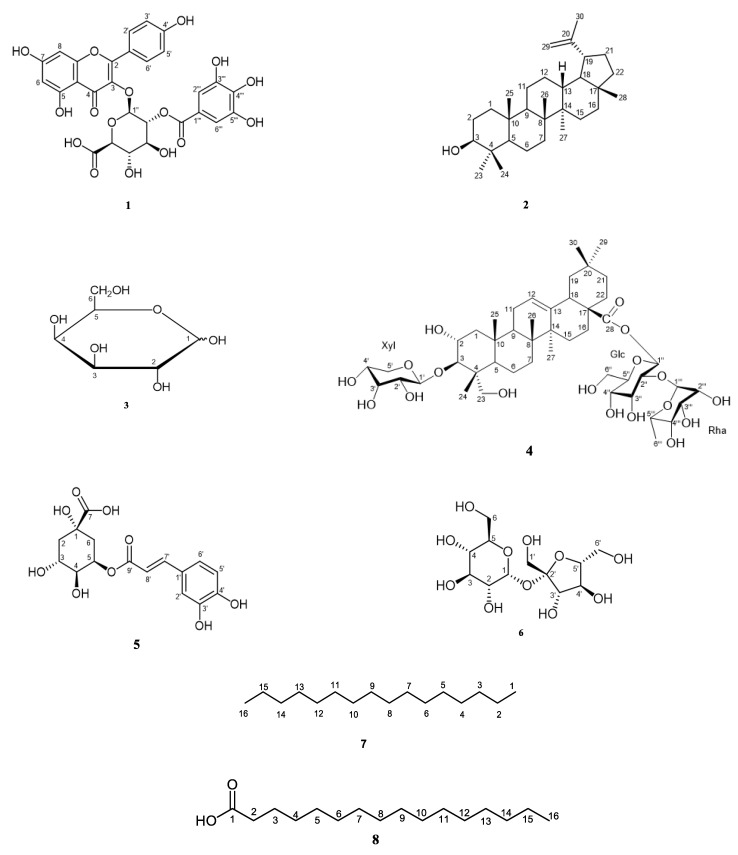
Compounds isolated from *B. salicina*: kaempferol 3-*O*-(2″-*O*-galloyl)-glucuronide (**1**), lupeol (**2**), d-galactopyranose (**3**), bodinioside Q (**4**), 5-*O*-caffeoylquinic acid (**5**), sucrose (**6**), hexadecane (**7**) and palmitic acid (**8**).

Fraction S_1_ (0.5 g) was subjected to preparative TLC (normal phase) to obtain compound **1** (0.28 g). Fraction S_2_ (0.34 g) was not purified due to having less amount of material. Fraction S_3_ (0.5 g) was also subjected to preparative TLC (normal phase) to obtain compound **2** (0.36 g). Fraction S_4_ (4 g) was subjected to silica gel column chromatography; the column was eluted using CH_2_Cl_2_/MeOH (50:50) followed by an increasing gradient of CH_2_Cl_2_/MeOH (up to 90:10) to obtain compounds **3** (0.27 g). Fraction R_2_ (4.89 g) was a pure fraction, yielded compound **4**. Fraction LM_1_ (4.46 g) was a pure fraction, yielded compound **5**. Fraction LM_3_ (5 g) was subjected to silica gel column chromatography; the column was eluted using CH_2_Cl_2_/EtOAc (50:50) followed by an increasing gradient elution with a mixture of dicholoromethane, ethyl acetate and methanol to yield compound **6** (0.96 g). Fraction LD_1_ was a pure fraction, yielded compound **7** (1.74 g). Fraction LD_3_ (2 g) was subjected to silica gel chromatography; the column was eluted using CH_2_Cl_2_/EtOAc (50:50) followed by an increasing gradient elution with a mixture of dicholoromethane, ethyl acetate and methanol to obtain compound **8** (0.13 g). Moreover, fractions S_5_ (17.10 g), R_1_ (4.44 g), LM_2_ (5.29 g) and LD_2_ (1.69 g) could not be purified due to the complexity of the fractions. Compounds **1** [50], **2** [23], **3** [46], **4** [51], **5** [24], **6** [20], **7** [52] and **8** [53] are known compounds, as shown in Figure 1.

Kaempferol 3-*O*-(2″-*O*-galloyl)-glucuronide (**1**): Yellow powder, m.p. 215–217 °C (lit, not reported). ^1^H-NMR (400 MHz, DMSO-d_6_): δ_H_ ppm: 8.25 (2H, d, H-2′, H-6′), 7.00 (2H, s, H-2″, H-6″), 6.70 (2H, d, H-3′, H-5′), 6.39 (1H, d, H-6), 6.25 (1H, d, H-8), 3.21 (1H, dd, H-2‴), 3.19 (1H, t, H-3‴). ^13^C-NMR (100 MHz, DMSO-d_6_): δ_C_ ppm: 168.0 (C-7″), 163.8 (C-7), 156.8 (C-5), 148.4 (C-2), 146.0 (C-3″, C-5″), 140.9 (C-4″), 132.8 (C-3), 130.2 (C-2′,6′), 121.0 (C-1′, C-1″), 115.4 (C-3′,5′), 109.1 (C-2″,6″), 106.3 (C-1‴), 99.4 (C-6), 94.2 (C-8), 79.4 (C-3‴), 79.2 (C-5‴), 74.0 (C-2‴), 71.25 (C-4‴). HRMS [M-H]^−^. *m/z* 613.08; calcd. for C_28_H_22_O_16_: 613.08129 (Figures S37–S40 [1]).

3β-Lup-20(29)-en-3-ol (lupeol, **2**): White powder, m.p. 217–218 °C (lit, 216–218 °C [54]). IR: ν_max_ (KBr): 3400 (O-H) cm^−1^, 2920.18 cm^−1^ (CH_2_), 2850.91 cm^−1^ (CH_3_) and 1686.74 cm^−1^ (C=C). ^1^H-NMR (400 MHz, CD_3_OD): δ_H_ (ppm): 4.72 (1H, s, 29a-H), 4.61 (1H, s, 29b-H), 3.02 (1H, m, 3-H), 2.23 (1H, m, 19-H), 1.91 (1H, m, 21-H), 1.71 (3H, s, 30-H), 1.52 (1H, m, 2-H), 1.43 (1H, m, 18-H), 1.30 (1H, m, 9-H), 1.02 (3H, s, 26-H), 0.98 (3H, s, 23-H), 0.96 (3H, s, 27-H), 0.87 (3H, s, 25-H), 0.77 (3H, s, 28-H), 0.74 (3H, s, 24-H). ^13^C-NMR (100 MHz, CD_3_OD): δ_H_ (ppm): 150.5 (C-20), 108.7 (C-29), 78.2 (C-3), 56.1 (C-5), 50.5 (C-9), 48.2 (C-18), 42.1 (C-14), 40.5 (C-8), 38.5 (C-4), 38.2 (C-1), 37.3 (C-13), 36.2 (C-10), 36.0 (C-16), 34.1 (C-7), 31.6 (C-21), 30.2 (C-23), 29.3 (C-2), 27.9 (C-15), 25.0 (C-12), 22.3 (C-11), 20.1 (C-30), 18.0 (C-6), 17.9 (C-28), 14.6 (C-26), 13.7 (C-24), 13.0 (C-27). HRMS [M-H]^−^: *m/z* 455.72; calcd. for C_28_H_22_O_16_: 455.3618 (Figures S41–S45 [2]).

d-Galactopyranose (**3**): Brown sticky oil. ^1^H-NMR (400 MHz, CD_3_OD): δ_H_ ppm: 5.16 (1H, d, H-1α), 4.52 (1H, dd, H-1β), 4.41 (1H, m, H-4β), 4.10 (1H, overlap, H-5α), 3.91 (1H, overlap, H-2α), 3.89 (1H, d, H-6), 3.71 (1H, m, H-5β), 3.32 (1H, m, H-3β). ^13^C-NMR (100 MHz, CD_3_OD): δ_C_ ppm: 97.8 (C-1β), 92.4 (C-1α), 76.4 (C-5β), 74.1 (C-3β), 73.4 (C-2β), 71.7 (C-2α), 70.3 (C-4β), 70.3 (C-3α), 70.3 (C-4α), 69.7 (C-5α), 63.1 (C-6β), 62.7 (C-6α). HRMS [M-H]^−^: *m/z* 179.156; calcd. for C_6_H_12_O_6_: 179.0557 (Figures S46–S48 [3]).

3-*O*-β-d-Xylopyranosyl-2α,23-dihydroxy-olean-12-en-28-oic acid 28-O-α-l-rhamnopyranosyl-(1→2)-β-d-glucopyranoside (bodinioside Q, **4**): Brown amorphous solid, m.p. 203–205 °C (lit, not reported). ^1^H-NMR (400 MHz, CD_3_OD): δ_H_ (ppm): 5.27 (1H, br. s, H-12), 4.48 (1H, m, H-23), 3.12 (1H, m, H-3), 2.85 (1H, d, H-18), 2.29 (1H, m, H-16), 2.20 (1H, m, H-15), 1.95 (1H, m, H-9), 1.70 (1H, m, H-19), 1.62 (1H, m, H-22), 1.46 (1H, m, H-6), 1.30 (2H, m, H-7), 1.27 (1H, m, H-21), 1.19 (3H, s, H-27), 1.14 (3H, s, H-26), 1.04 (3H, s, H-25), 0.95 (3H, s, H-24), 0.92 (1H, m, H-5), 0.86 (3H, s, H-30), 0.82 (3H, s, H-29); 3-O-sugar: Xyl: 5.12 (1H, d, H-1′), 4.62 (1H, overlap, H-5′), 4.10 (1H, overlap, H-3′), 3.80 (1H, overlap, H-2′), 28-O-sugar: Glc: 4.14 (1H, t, H-4″), 4.12 (1H, overlap, H-5″), 4.05 (1H, t, H-2″); Rha: 1.67 (1H, d, H-6‴). ^13^C-NMR (100 MHz, CD_3_OD): δ_C_ (ppm): 145.1 (C-13), 122.0 (C-12), 108.5 (C-28), 92.5 (C-3), 68.2 (C-2), 63.7 (C-23), 50.7 (C-9,17), 46.7 (C-1), 46.2 (C-5), 45.8 (C-19), 44.5 (C-14), 42.0 (C-18), 41.6 (C-4), 41.3 (C-8), 37.6 (C-10), 34.5 (C-21), 33.5 (C-7,22,29), 30.4 (C-27), 30.2 (C-20), 29.1 (C-15), 23.9 (C-11), 23.0 (C-30), 22.7 (C-16), 20.6 (C-6), 17.6 (C-25), 17.4 (C-26), 13.0 (C-24); 3-O-sugar: Xyl 107.8 (C-1′), 81.8 (C-3′), 76.0 (C-2′), 71.5 (C-4′), 68.0 (C-5′); 28-O-sugar: Glc 96.7 (C-1″), 82.8 (C-3″), 81.8 (C-5″), 75.6 (C-2″), 70.4 (C-4″), 61.4 (C-6″); Rha 101.7 (C-1‴), 74.8 (C-4‴), 73.5 (C-3‴), 72.4 (C-2‴), 69.8 (C-5‴), 19.4 (C-6‴). HRMS [M-H]^−^: *m/z* 487 [M–Glc–Rha–Xyl–H]^−^; calcd. for C_47_H_76_O_18_: *m/z*: 487.3448 [M–Glc–Rha–Xyl–H]^−^ (Figures S49–S58 [4]).

5-*O*-Caffeolquinic acid (**5**): Green powder, m.p. 207–209 °C (lit, 209 °C [55]). ^1^H-NMR (400 MHz, CD_3_OD): quinic moiety δ_H_ ppm: 5.24 (1H, m, H-5), 3.66 (1H, m, H-3), 3.32 (1H, m, H-4), 2.29–1.93 (1H, m, H-6), 1.70–1.51 (1H, m, H-2); caffeoyl moiety δ_H_ ppm: 7.52 (1H, d, H-8′), 7.06 (1H, d, H-2′), 6.95 (1H, dd, H-6′), 6.79 (1H, d, H-5′), 6.21 (1H, d, H-7′). ^13^C-NMR (100 MHz, CD_3_OD): quinic moiety δ_C_ ppm: 76.4 (C-1), 74.3 (C-5), 70.7 (C-4), 68.8 (C-3), 41.8 (C-6), 36.6 (C-2); caffeoyl moiety δ_C_: 180.2 (COO-), 166.5 (C-9′), 148.5 (C-4′), 145.8 (C-3′), 145.4 (C-7′), 126.2 (C-1′), 121.6 (C-6′), 115.1 (C-5′), 113.6 (C-2′), 113.6 (C-8′) ppm. HRMS [M-H]^−^: *m/z* 367.311; calcd. for C_16_H_18_O_9_: 367.1107 (Figures S59–S63 [5]).

*O*-α-d-Glucopyranosyl-(1→2)-β-d-fructofuranoside (sucrose, **6**): Brown sticky oil. ^1^H-NMR (400 MHz, CD_3_OD): δ_H_ ppm: 5.41 (d, 1H, H-1), 4.14 (d, 1H, H-3′), 4.02 (t, 1H, H-4′), 3.86 (m, 1H, H-5′), 3.83 (m, 1H, H-5), 3.81 (d, 2H, H-6), 3.80 (d, 2H, H-6′), 3.72 (t, 1H, H-3), 3.63 (s, 2H, H-1′), 3.50 (dd, 1H, H-2), 3.39 (t, 1H, H-4). ^13^C-NMR (100 MHz, CD_3_OD): δ_C_ ppm: 103.8 (C-2′), 92.2 (C-1), 81.6 (C-5′), 77.8 (C-3′), 72.9 (C-4′), 72.2 (C-3), 72.0 (5), 70.2 (C-2), 67.9 (C-4), 62.5 (C-6′), 61.2 (C-1), 60.7 (C-6). HRMS [M-H]^−^: *m/z* 341.30; calcd. for C_12_H_22_O_11_: 341.1073 (Figures S64–S69 [6]).

Hexadecane (**7**): White crystals, m.p. 16–18 °C (lit, 18 °C [56]). ^1^H-NMR (400 MHz, CDCl_3_): δ_H_ (ppm): 1.28 (28H, m, H-2), 1.28 (28H, m, H-3), 1.28 (28H, m, H-4), 1.28 (28H, m, H-5), 1.28 (28H, m, H-6), 1.28 (28H, m, H-7), 1.28 (28H, m, H-8), 1.28 (28H, m, H-9), 1.28 (28H, m, H-10), 1.28 (28H, m, H-11), 1.28 (28H, m, H-12), 1.28 (28H, m, H-13), 1.28 (28H, m, H-14), 1.28 (28H, m, H-15), 0.89 (6H, t, H-1, H-16). ^13^C-NMR (100 MHz, CDCl_3_): δ_C_ (ppm): 31.95 (C-3,14), 29.3–29.7 (C-4, C-5, C-6, C-7, C-8, C-9, C-10, C-11, C-12, C-13, C-14), 22.7 (C-2,15), 14.1 (C-1,6), (Figures S70–S71 [7]).

Palmitic acid (**8**): Green amorphous solid, m.p. 61–62 °C (lit, 63 °C [57]). IR: ν_max_ (KBr): 3436.00 cm^−1^ (O-H), 2917.27 cm^−1^ (CH_2_), 2849.51 (CH_3_) and 1703.71 cm^−1^ (C=O).^1^H-NMR (400 MHz, CDCl_3_): δ_H_ (ppm): 2.27 (2H, t, H-2), 1.98 (2H, q, H-15), 1.57 (2H, m, H-3), 1.23 (2H, m, H-4, H-5, H-6, H-7, H-8, H-9, H-10, H-11, H-12, H-13), 0.83 (3H, t, H-16). ^13^C-NMR (100 MHz, CDCl_3_): δ_C_ (ppm): 178.4 (C-1), 34.0 (C-2), 31.9 (C-3), 29.0–29.6 (C-4, C-5, C-6, C-7, C-8, C-9, C-10, C-11, C-12, C-13), 24.7 (C-14), 22.6 (C-15), 14.1 (C-16). HRMS [M-H]^−^: *m/z* 255.40; calcd. for C_16_H_32_O_2_: 255.23295 (Figures S72–S75 [8]).

### 3.5. Antioxidant Activities

#### 3.5.1. Free Radical Scavenging Assay (DPPH)

The DPPH free radical scavenging activity of the crude extracts, fractions and pure compounds were determined according to the modified spectrophotometric method of Motamed and Naghibi (2010) [58]. A solution of 125 mM DPPH/methanol was prepared by dissolving 10 mg DPPH (2,2-diphenyl-1-picrylhydrazyl) in 200 mL methanol. A 100 μL volume of distilled water was added in each 96-well plate. Therefore, a 100 μL volume of the crude extracts, fractions and pure compounds was added in triplicate into the first three wells followed by serial dilution using a multi-channel micropipette. Finally, a volume of 200 μL of DPPH (2,2-diphenyl-1-picrylhydrazyl) was added to each well containing the mixtures and the 96-well plate was kept in the dark for not more than 30 min. The absorbance was evaluated using a VersaMax**^TM^** tuneable microplate reader at 517 nm.

The percentage radical scavenging was determined by using the following formula:

% Free RSA = [(A_DPPH_ − A_sample_)/(A_DPPH_)] × 100 (1)

#### 3.5.2. Reducing Power

The reducing power was determined according to the modified method of Pereira et al. (2013) [59]. A volume of 100 μL of the samples (crude extracts, fractions and pure compounds) and standards (ascorbic acid and gallic acid) was added in triplicate in the first three wells of a 96-well plate, each containing 100 μL of deionized water, followed by serial dilution. A volume of 0.2 M (pH 6.6) sodium phosphate buffer (50 μL) was added into all 96-well plates and 50 μL volume of a 1% aqueous potassium hexacyanoferrate(III) [K_3_Fe (CN)_6_] solution was added in each well. The mixture was incubated for 20 minutes at 45 °C. After incubation, a volume of 50 μL of 10% trichloroacetic acid solution was added to each well. An 80 μL volume of each mixture was transferred to another 96-well plate containing a volume of 80 μL of distilled water and 16 μL ferric chloride (0.1% *w/v*). Absorbance was determined using a VersaMax™ tuneable microplate reader at 700 nm.

### 3.6. Statistical Analysis

Statistical analysis was undertaken using the SPSS package (Chicago, IL, USA). The data was shown as mean ± standard deviation (SD). Mean differences of the crude extracts, fractions and pure compounds were assessed by one-way analysis of variance (ANOVA, Graph pad prism 6) in the antioxidant tests; *p* < 0.05 was considered statistically significant.

## 4. Conclusions

The main objective of this study was to determine the phytochemistry, phytochemical compositions and antioxidant activity of *Breonadia salicina.* Eight compounds (kaempferol 3-*O*-(2″-*O*-galloyl)-glucuronide, lupeol, d-galactopyranose, bodinioside Q, 5-*O*-caffeoylquinic acid, sucrose, hexadecane and palmitic acid) were isolated from the stem bark, root and leaf extracts of *B. salicina*. This is the first study to report the isolation of these compounds from the genus *Breonadia* Ridsdale and *B. salicina* species. Consequently, a total of 25 metabolites were tentatively identified from different parts of *B. salicina* using UPLC-QTOF-MS. This is the first study to identify and report these metabolites from the genus *Breonadia* Ridsdale and *B. salicina* species. Furthermore, the study showed that the stem bark crude extract contained a significantly higher antioxidant capacity compared to the root and leaf samples. The findings in this work comprehensively indicate that polyphenols, hydroxycinnamic acids and flavonoids contribute to the biological activities evaluated.

## Figures and Tables

**Table 1:** ^1^H-NMR (δ_H_ ppm) signals of identified metabolites in *B. salicina* extracts and fractions.

Metabolites	^1^H-NMR (δ_H_ ppm)	Samples
Catechin	7.05 (1H, d), 6.72–6.85 (1H, dd), 5.86 (1H, s), 5.94 (1H, s).	fraction S_1_
	7.06 (1H, d), 6.72–6.86 (1H, dd), 5.87 (1H, s), 5.94 (1H, s), 4.57 (1H, d).	fraction S_2_
Lupeol	1.30 (1H, m), 1.04 (3H, s), 0.96 (3H, s), 0.92 (3H, s), 0.83 (3H, s), 0.71 (3H, s).	R.crude
	4.60 (1H, s), 4.72 (1H, s), 1.02 (3H, s), 1.56 (1H, m), 1.61 (1H, m), 1.71 (3H, s), 1.91 (1H, m), 2.23 (1H, m), 3.12 (1H, m), 0.98 (3H, s), 0.96 (3H, s), 0.87 (3H, s), 0.77 (3H, s).	fraction S_1_
	1.36 (1H, m), 1.02 (3H, s), 0.98 (3H, s), 0.96 (3H, s), 0.87 (3H, s), 0.77 (3H, s).	fraction S_2_
5-*O*-Caffeoylquinic acid	7.60 (1H, d), 7.67 (1H, d), 6.96 (1H, dd), 6.79 (1H, d), 6.30 (1H, d).	LM.crude
	7.52 (1H, d), 7.06 (1H, d), 6.96 (1H, dd), 6.79 (1H, d).	fraction LM_2_
	7.61 (1H, d), 7.07 (1H, d), 6.95 (1H, dd), 6.79 (1H, d), 6.295 (1H, d).	fraction LM_3_
Hexadecane	1.69 (28H, m), 0.90 (6H, t).	LD.crude
	1.27 (28H, m), 0.84 (6H, t).	fraction R1
	1.27 (28H, m), 0.87 (6H, t).	fraction LD3
α-Glucose	5.12 (d).	R.crude
	5.12 (d).	S.crude
	5.13 (d).	LM.crude
	5.13 (d).	fraction LM_3_
β-Glucose	4.48 (d), 3.12 (m).	R.crude
	4.49 (d), 3.13 (m).	S.crude
	3.01 (m).	LM.crude
	4.50 (d), 3.01 (m).	fraction LM_3_
Glucose and fructose	3.63–3.80 (m).	R.crude
	3.62–3.80 (m).	S.crude
	3.61–3.80 (m).	LM.crude
	3.69–3.81 (m).	fraction LM_3_

**Table 2:** Identification of constituents from *Breonadia salicina* by UPLC-QTOF-MS.

Peak No.	Rt (min)	Theoretical Mass [M-H]^−^(*m/z*)	Observed Mass [M-H]^−^(*m/z*)	Molecular Formula	MS/MS Fragment Ions (*m/z*)	Compound Name	Compound Class	Samples	References
1	0.661	377.0878	377.08633	C_18_H_18_O_9_	341,215,160	Caffeic acid derivative	hydroxycinnamic acid	S.crude	[25]
2	14.908	461.0720	461.07350	C_21_H_18_O_12_	315,299	4′-*O*-Methyellagic acid-3-*O*-α-L-rhamnopyranoside	polyphenol	S.crude	[26]
3	12.782	300.999	300.99929	C_14_H_6_O_8_	242,174	Ellagic acid	polyphenol	S.crude	[27]
4	13.002	447.0569	447.05763	C_20_H_16_O_12_	300.99898	Ellagic acid-rhamnopyranoside isomer I	hydrolyzable tannin	S.crude, fraction S_5_	[27]
5	5.460	289.0707	289.07225	C_15_H_14_O_6_	245.08155	Catechin	flavonoid	fraction S_1_, fraction S_2_, fraction S_3_	[28]
6	21.504	485.3271	485.32825	C_30_H_46_O_5_	485,441	Hydroxyglycyrrhetinic acid	triterpenoid	fraction S_1_	[29]
7	21.767	458.3396	458.33667	C_29_H_46_O_4_	503,457	Neotigogenin acetate	triterpenoid	fraction S_1_	[30]
8	22.507	499.3424	499.34385	C_31_H_48_O_5_	455.35408	25-Hydroxy-3-epi-dehydrotumulosic acid	triterpenoid	fraction S_1_	[31]
9	23.373	453.3347	453.33834	C_30_H_46_O_3_	-	Micromeric acid	triterpenoid	fraction S_1_	[32]
10	24.467	499.3736	499.37155	C_32_H_50_O_4_	497.36498	3-Acetylursolic acid	triterpenoid	fraction S_1_, fraction S_2_	[33]
11	1.876	305.0	305.06698	C_15_H_14_O_7_	-	(Epi) Gallocatechin	flavan-3-ol	fraction S_2_	[34]
12	4.728	183.0299	183.02975	C_8_H_8_O_5_	184.03304	4-*O*-Methylgallic acid	phenolic acid	fraction S_2_	[35]
13	11.530	479.0831	479.08529	C_21_H_20_O_13_	480.08721	Myricetin 3-*O*-glucoside	flavonoid	fraction S_3_, fraction S_4_	[36]
14	22.882	455.35412	455.35397	C_30_H_48_O_3_	-	Ursolic acid	triterpenoid	S.crude, fraction S_1_	[37]
15	21.610	487.35	487.33699	C_30_H_48_O_5_	485.32825	Asiatic acid	triterpenoid	fraction S_3_, fraction R_1_	[38]
16	12.569	433.0412	433.04194	C_19_H_14_O_12_	300.99929	Ellagic acid pentoside	polyphenol	R.crude	[27]
17	1.140	170	170.01742	C_7_H_6_O_5_	-	Gallic acid	phenolic acid	fraction R_1_	[39]
18	0.663	533.1738	533.17341	C_19_H_34_O_17_	191.05605	Quinic acid + hexose_2_	quinic acids and derivatives + monosaccharide	LM.crude, fraction LM_3_	[40]
19	6.415	353.08685	353.08839	C_16_H_18_O_9_	191,707	Chlorogenic acid [3.4-Dihydroxycinnamoylquinic acid; 5-Caffeoylquinic acid]	quinic acids	LM.crude	[41]
20	8.421	389.1088	389.10941	C_16_H_22_O_11_	390.11317	Deacetyl asperuloside acid	monoterpenoid	LM.crude	[42]
21	10.819	367.10346	367.10424	C_17_H_20_O_9_	174.95588	5-Methyl caffeoylquinic acid	quinic acid	LD.crude, fraction LM_2_	[43]
22	10.181	451.1029	451.10412	C_24_H_20_O_9_	341.06822	Cinchonain I isomer	flavonolignan	LM.crude	[26]
23	12.918	609.1464	609.14777	C_27_H_30_O_16_	463,447	Rutin	flavonoid glycoside	LM.crude, fraction LM_3_	[44]
24	14.242	515.5	515.12070	C_25_H_24_O_12_	353.08946	Di-*O*-Caffeoylquinic acid	quinic acid	LM.crude, fraction LM_3_	[45]
25	0.671	191.1	191.05581	C_7_H_12_O_6_	-	Quinic acid	quinic acids and derivatives	LD.crude, fraction LM_2_	[45]

**Table 3:** Antioxidant activity of crude extracts, fractions, pure compounds and controls.

Sample	DPPH IC_50_ (μg/mL)	Reducing Power IC_0.5_ (μg/mL)
S.crude	41.7263 ± 7.6401 ^a^	1.0738 ± 1.4316 ^a^
S_1_	49.3931 ± 0.2657 ^a^	2.7258 ± 3.5872 ^a^
S_2_	49.0216 ± 1.1209 ^a^	0.9902 ± 0.3556 ^a^
S_3_	49.6295 ± 0.1562 ^a^	0.2499 ± 0 ^a^
S_4_	48.2396 ± 0.2007 ^a^	0.1942 ± 0.0464 ^a^
S_5_	46.0939 ± 0.9941 ^a^	0.2502 ± 0.0003 ^a^
R.crude	46.569 ± 1.8444 ^a,b^	0.1481 ± 0.1441 ^a^
R_1_	45.2806 ± 0.7117 ^a^	12.5572 ± 16.7165 ^a^
LM.crude	47.3590 ± 0.7794 ^a,c^	8.5739 ± 10.1838 ^a^
LM_2_	48.4597 ± 0.6525 ^a^	1.1925 ± 0.0849 ^a^
LM_3_	45.4784 ± 1.0390 ^a,^	2.1748 ± 1.3042 ^a^
LD.crude	47.3397 ± 1.0680 ^a,d^	2.4379 ± 1.4826 ^a^
LD_2_	45.1968 ± 3.1969 ^a^	2.5178 ± 1.1822 ^a^
LD_3_	49.12 ± 0.5357 ^a^	4.1584 ± 1.7431 ^a^
Kaempferol 3-*O*-(2″-*O*-galloyl)-glucuronide (**1**)	46.9493 ± 0.1388 ^a^	3.3742 ± 1.7492 ^a^
Lupeol (**2**)	95.1091 ± 0.1501 ^a,b,c,d^	32.3413 ± 0 ^a^
d-Galactopyranose (**3**)	44.5613 ± 2.6772 ^a^	9.7237 ± 0.1625 ^a^
Bodinioside Q (**4**)	48.9097 ± 0.2266 ^a^	10.9919 ± 6.3849 ^a^
5-*O*-Caffeoylquinic acid (**5**)	48.1673 ± 0.1246 ^a^	16.7798 ± 0 ^a^
Sucrose (**6**)	47.3525 ± 0.0380 a	7.7263 ± 0 a
Hexadecane (**7**)	91.5285 ± 0.1032 a	32.0310 ± 0.022 a
Palmitic acid (**8**)	94.4295 ± 0.9197 ^a,b,c,d^	31.3131 ± 1.0497 ^a^
Ascorbic acid	48.0304 ± 2.6010 ^a^	3.4143 ± 0.1117 ^a^
Gallic acid	49.2369 ± 0.7411 ^a^	1.2361 ± 0.0352 ^a^

Notes: A different superscript letter indicates significant differences using one-way ANOVA at *p* < 0.05. Data (n = 3) expressed as mean ± standard deviation. For DPPH (2,2-diphenyl-1-picrylhydrazyl) free radical scavenging activity: ^a^—Stem bark crude extract (S.crude) was significantly different to all samples; ^a,b^—Root crude extract (R.crude) was only significantly different to lupeol (**2**) and palmitic acid (**7**); ^a,c^—Methanol leaf crude extract (LM.crude) was only significantly different to lupeol (**2**) and palmitic acid (**7**); ^a,d^—Dichloromethane leaf crude extract (LD.crude) was only significantly different to lupeol (**2**) and palmitic acid (**7**), and ^a,b,c,d^—lupeol (**2**) was only significantly different to palmitic acid (**7**). For reducing power activity: ^a^—Stem bark crude extract was not significantly different to all samples.

## Data Availability

Data is contained within the article and Supplementary Materials.

## Supplementary Materials

The following are available online, Figure S1A. The representative 1H-NMR spectra of fraction S1. 1, catechin.; Figure S1B. The representative 1H-NMR spectra of fraction S1. 3, lupeol.; Figure S2. The representative 1H-NMR spectra of fraction S2. 1, catechin; Figure S3. The representative 1H-NMR spectra of fraction S2. 2, lupeol; Figure S4. The representative 1H-NMR spectra of R.crude. 1, α-glucose; 2, β-glucose; 3, glucose and fructose; 4, lupeol; Figure S5. The representative 1H-NMR spectra of LM.crude. 1, 5-O-caffeoylquinic acid; 2, α-glucose; 3, glucose and fructose; 4, β-glucose; Figure S6. The representative 1H-NMR spectra of fraction LM2. 1, 5-O-caffeoylquinic acid; Figure S7. The representative 1H-NMR spectra of fraction LM3. 1, 5-O-caffeoylquinic acid; 2, α-glucose; 3, β-glucose; 4, glucose and fructose; Figure S8. The representative 1H-NMR spectra of LD.crude. 1, hexadecane; Figure S9. The representative 1H-NMR spectra of fraction R1. 1, hexadecane; Figure S10. The representative 1H-NMR spectra of fraction LD3. 1, hexadecane; Figure S11. The representative 1H-NMR spectra of S.crude. 1, α-glucose; 2, β-glucose; 3, glucose and fructose; Figure S12. Molecular ion of caffeic acid derivative; Figure S13. Molecular ion of 4′-O-methyellagic acid-3-O-α-L-rhamnopyranoside; Figure S14. Molecular ion of ellagic acid; Figure S15. Molecular ion of ellagic acid-rhamnopyranoside isomer I; Figure S16. Molecular ion of catechin; Figure S17. Molecular ion of hydroxyglycyrrhetinic acid; Figure S18. Molecular ion of neotigogenin acetate; Figure S19. Molecular ion of 25-hydroxy-3-epi-dehydrotumulosic acid; Figure S20. Molecular ion of micromeric acid; Figure S21. Molecular ion of 3-acetylursolic acid; Figure S22. Molecular ion of (epi) gallocatechin; Figure S23. Molecular ion of 4-O-methylgallic acid; Figure S24. Molecular ion of myricetin 3-O-glucoside; Figure S25. Molecular ion of ursolic acid; Figure S26. Molecular ion of asiatic acid; Figure S27. Molecular ion of ellagic acid pentoside; Figure S28. Molecular ion of gallic acid; Figure S29. Molecular ion of quinic acid + hexose2; Figure S30. Molecular ion of chlorogenic acid [3.4-dihydroxycinnamoylquinic acid; 5-caffeoylquinic acid]; Figure S31. Molecular ion of deacetyl asperuloside acid; Figure S32. Molecular ion of 5-methyl caffeoylquinic acid; Figure S33. Molecular ion of cinchonain I isomer; Figure S34. Molecular ion of rutin; Figure S35. Molecular ion of di-O-caffeoylquinic acid; Figure S36. Molecular ion of quinic acid; Figure S37. Mass spectrum of kaempferol 3-O-(2″-O-galloyl)-glucuronide (1) [1]; Figure S38. Expanded 1H-NMR spectrum of kaempferol 3-O-(2″-O-galloyl)-glucuronide (1) [1]; Figure S39. Expanded 1H-NMR spectrum of kaempferol 3-O-(2″-O-galloyl)-glucuronide (1) [1]; Figure S40. 13C-NMR spectrum of kaempferol 3-O-(2″-O-galloyl)-glucuronide (1) [1]; Figure S41. Mass spectrum of lupeol (2) [2]; Figure S42. IR spectrum of lupeol (2) [2]; Figure S43. Expanded 1H-NMR spectrum of lupeol (2) [2]; Figure S44. 13C-NMR spectrum of lupeol (2) [2]; Figure S45. Expanded 13C-NMR spectrum of lupeol (2) [2]; Figure S46. Mass spectrum of D-galactopyranose (3) [3]; Figure S47. Expanded 1H-NMR spectrum of D-galactopyranose (3) [3]; Figure S48. 13C-NMR spectrum of D-galactopyranose (3) [3]; Figure S49. Mass spectrum of bodinioside Q (4) [4]; Figure S50. Expanded 1H-NMR spectrum of bodinioside Q (4) [4]; Figure S51. Expanded 1H-NMR spectrum of bodinioside Q (4) [4]; Figure S52. 13C-NMR spectrum of bodinioside Q (4) [4]; Figure S53. 13C-NMR spectrum of bodinioside Q (4) [4]; Figure S54. 13C-NMR spectrum of bodinioside Q (4) [4]; Figure S55. HSQC spectrum of bodinioside Q (4) [4]; Figure S56. HMBC spectrum of bodinioside Q (4) [4]; Figure S57. Expanded HMBC spectrum of bodinioside Q (4) [4]; Figure S58. Expanded HMBC spectrum of bodinioside Q (4) [4]; Figure S59. Mass spectrum of 5-O-caffeoylquinic acid (5) [5]; Figure S60. 13C-NMR spectrum of 5-O-caffeoylquinic acid (5) [5]; Figure S61. Expanded 1H-NMR spectrum of 5-O-caffeoylquinic acid (5) [5]; Figure S62. Expanded 1H-NMR spectrum of 5-O-caffeoylquinic acid (5) [5]; Figure S63. Expanded 1H-NMR spectrum of 5-O-caffeoylquinic acid (5) [5]; Figure S64. MS spectrum of sucrose (6) [6]; Figure S65. Expanded 1H-NMR spectrum of sucrose (6) [6]; Figure S66. Expanded 1H-NMR spectrum of sucrose (6) [6]; Figure S67. 13C-NMR spectrum of sucrose (6) [6]; Figure S68. COSY spectrum of sucrose (6) [6]; Figure S69. HMBC spectrum of sucrose (6) [6]; Figure S70. Expanded 1H-NMR spectrum of hexadecane (7) [7]; Figure S71. 13C-NMR spectrum of hexadecane (7) [7]; Figure S72. IR spectrum of palmitic acid (8) [8]; Figure S73. MS spectrum of palmitic acid (8) [8]; Figure S74. Expanded 1H-NMR spectrum of palmitic acid (8) [8]; Figure S75. 13C-NMR spectrum of palmitic acid (8) [8].

## Sample Availability

All samples of the compounds are available from the authors.
