# Supplementary material for: Metabolomic Profiling and Antioxidant Activities of Breonadia salicina Using 1H-NMR and UPLC-QTOF-MS Analysis
Source: Molecules. 2021 Nov 5;26(21):6707. doi: 10.3390/molecules26216707 (PMC8587154; doi:10.3390/molecules26216707)
Supplement: Supplementary file 1 [file molecules-26-06707-s001.zip › molecules-1402658-supplementary.pdf]

molecules

MDPI

Supplementary information

Metabolomic Profiling and Antioxidant Activities of *Breonadia Salicina* Using ^{1}H-NMR and UPLC-QTOF-MS Analysis

Dorcas B. Tlhapi [1], Isaiah D. I. Ramaite [1,*], Chinedu P. Anokwuru [2]

[1] Department of Chemistry, University of Venda, Private Bag X5050, Thohoyandou 0950, South Africa; dorcastlhapi@gmail.com (D.B.T.)

[2] Department of Pharmaceutical Sciences, Faculty of Science, Tshwane University of Technology, Pretoria, 0001; anokwuruchi@gmail.com

* Correspondence: Isaiah.Ramaite@univen.ac.za; Tel.: +27(0)15-962-8262

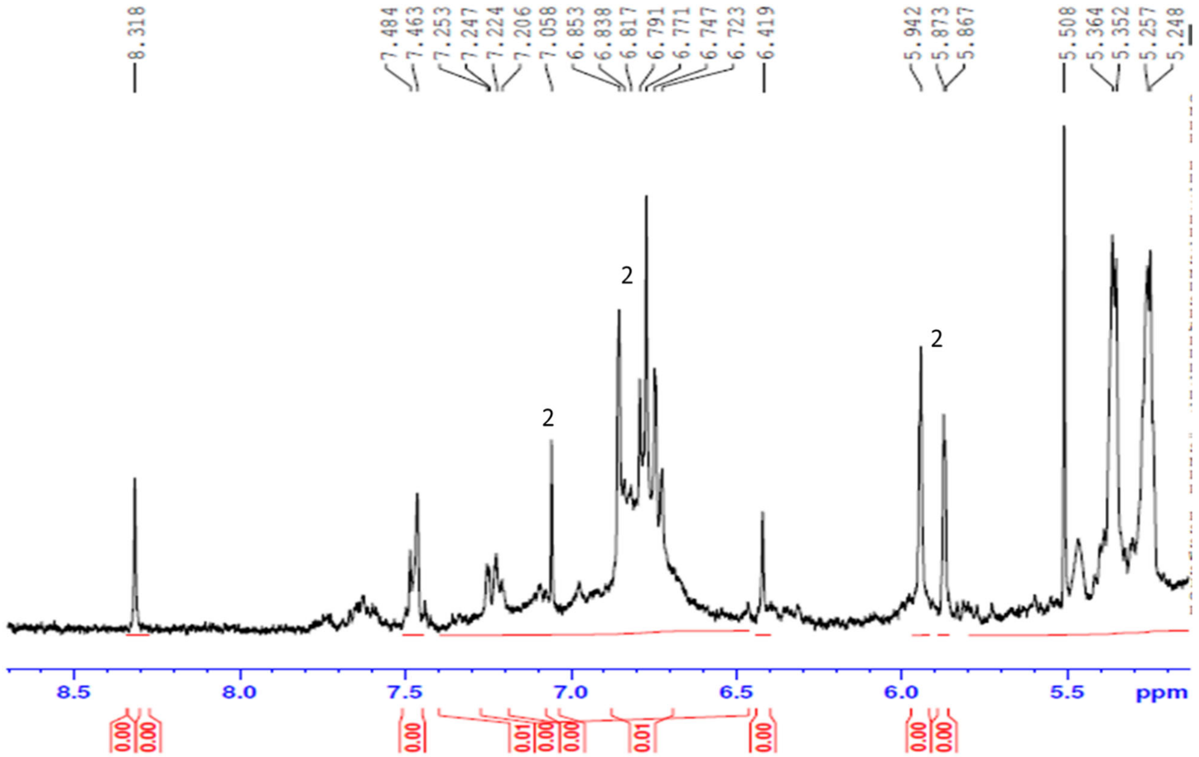

Figure S1A. The representative ^{1}H-NMR spectra of fraction S1. 1, catechin.

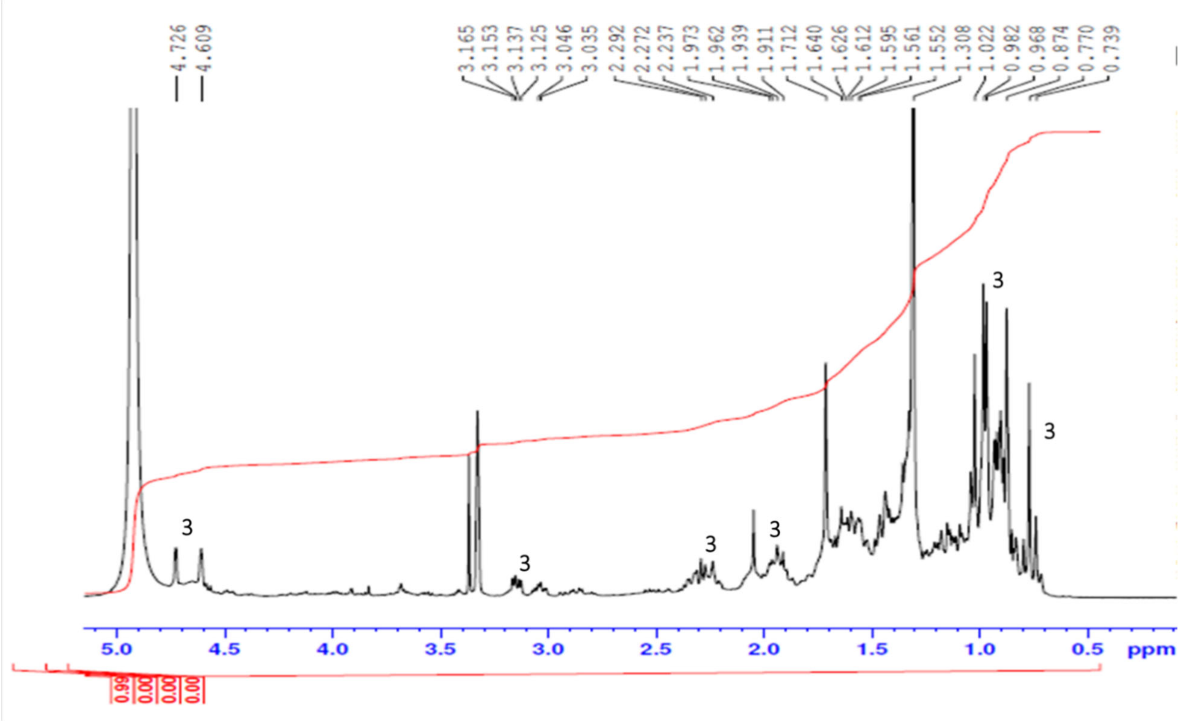

Figure S1B. The representative ^{1}H-NMR spectra of fraction S_1. 3, lupeol.

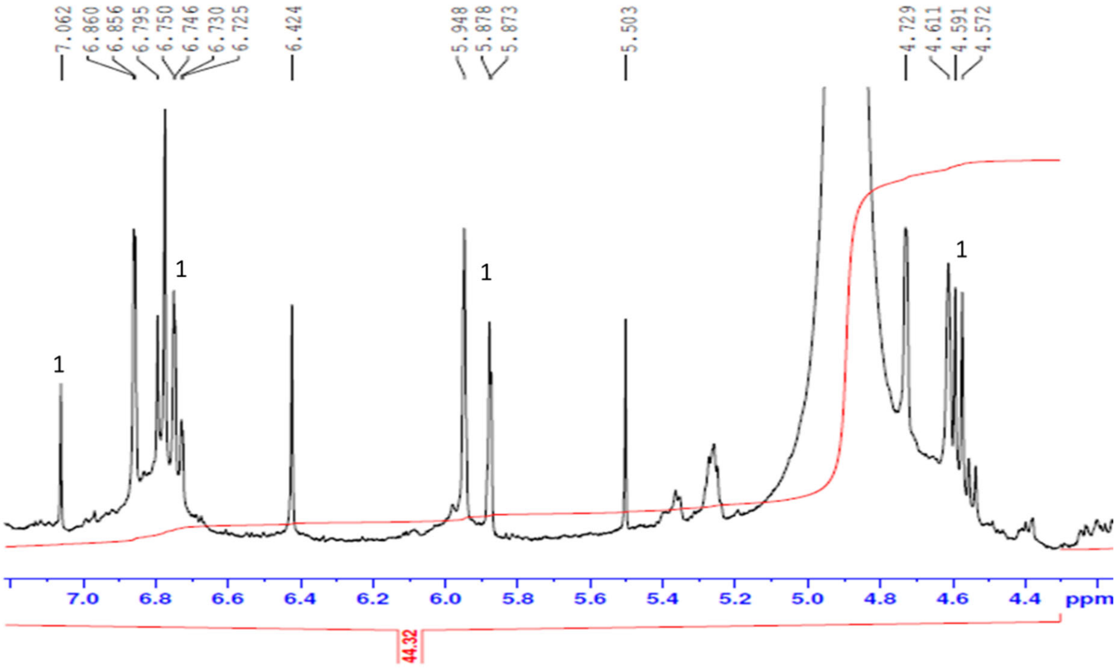

Figure S2. The representative ^{1}H-NMR spectra of fraction S_2. 1, catechin.

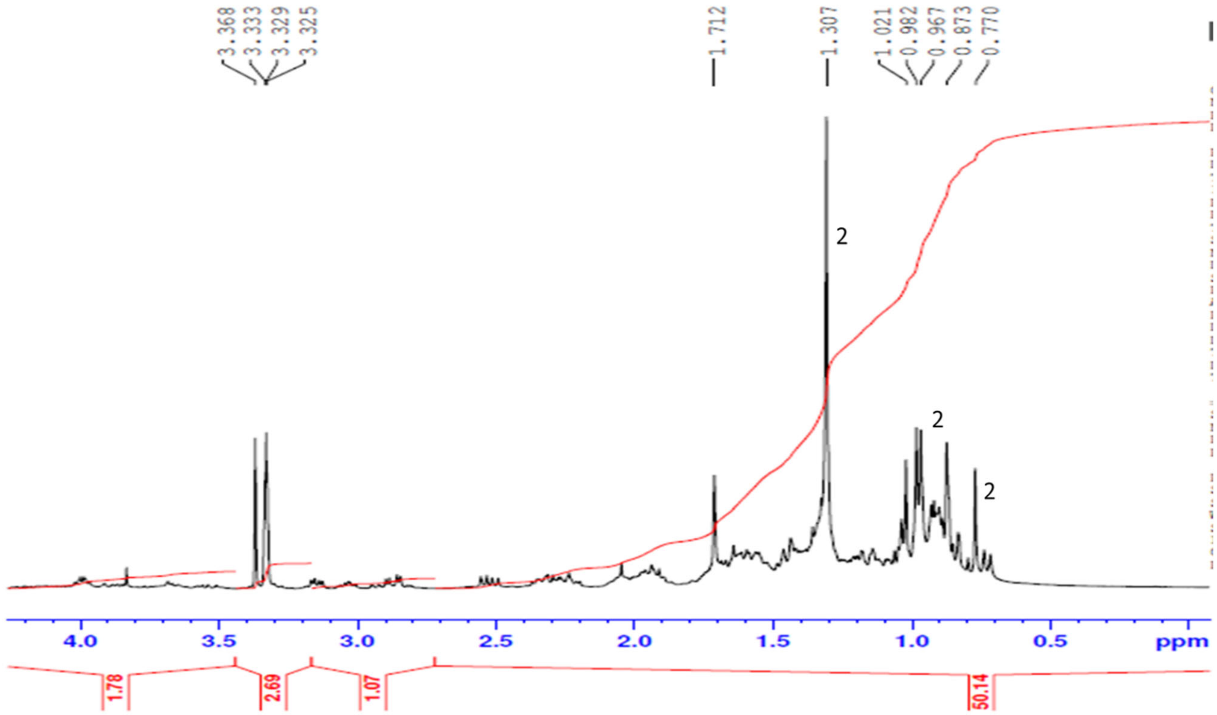

Figure S3. The representative ^{1}H-NMR spectra of fraction S_2. 2, lupeol.

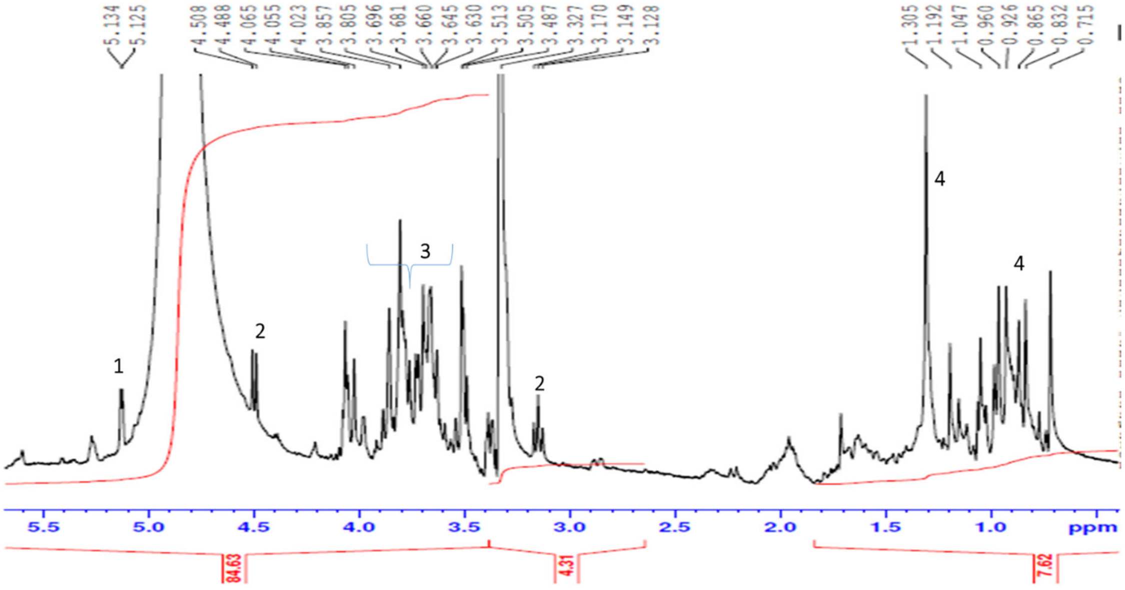

Figure S4. The representative ^{1}H-NMR spectra of R.crude. 1, α-glucose; 2, β-glucose; 3, glucose and fructose; 4, lupeol.

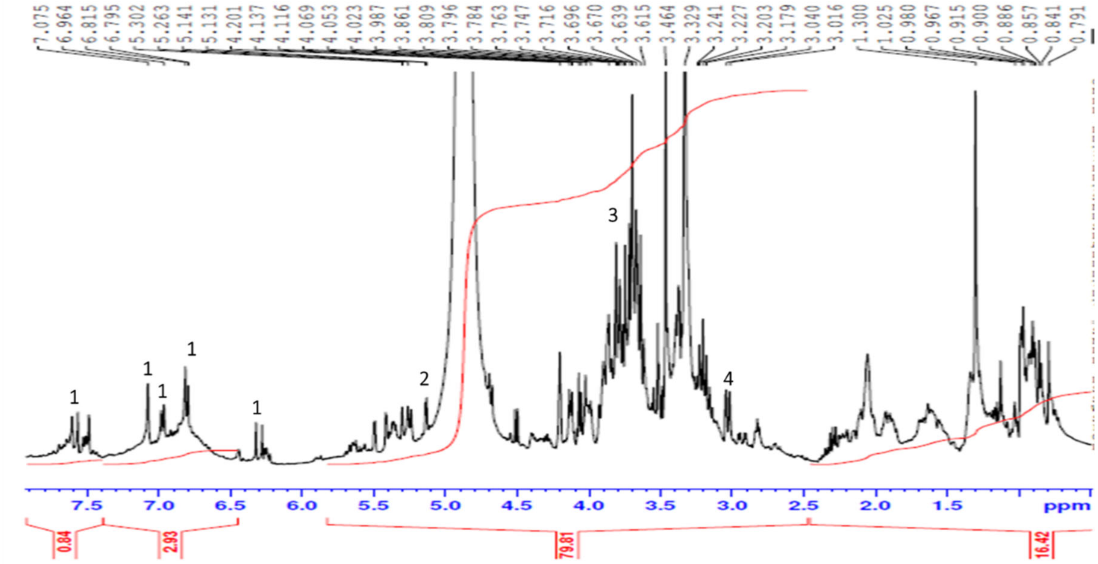

Figure S5. The representative ^{1}H-NMR spectra of LM.crude. 1, 5-*O*-caffeoylquinic acid; 2, α-glucose; 3, glucose and fructose; 4, β-glucose.

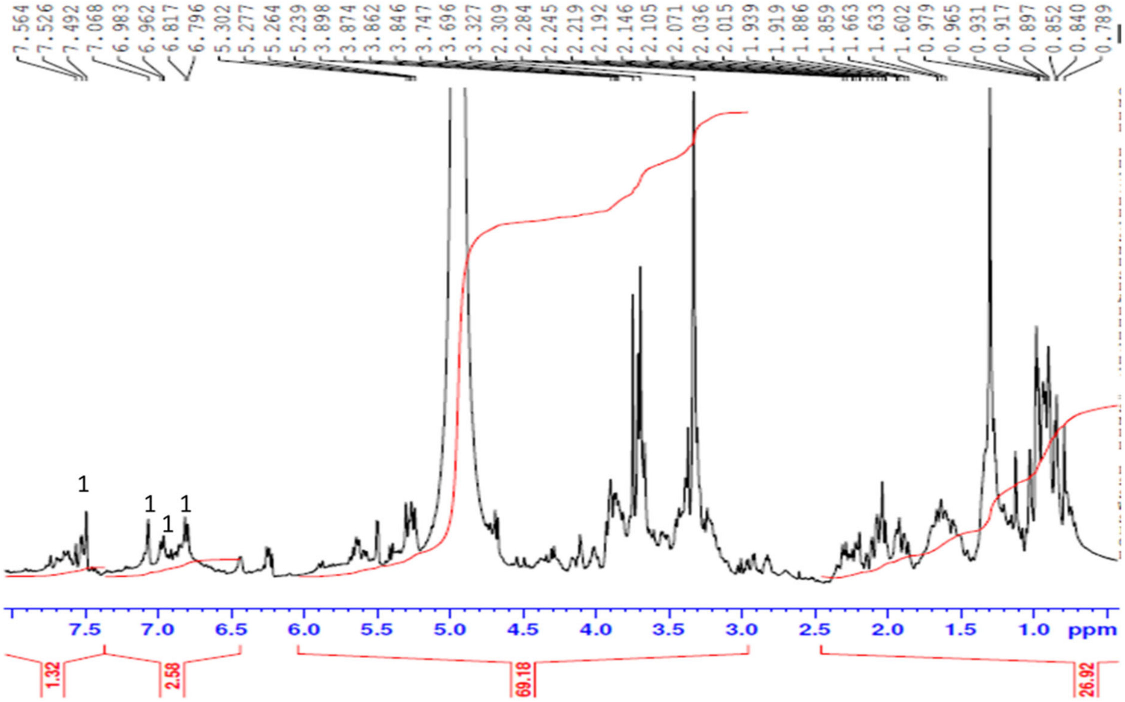

Figure S6. The representative ^{1}H-NMR spectra of fraction LM_2. 1, 5-*O*-caffeoylquinic acid.

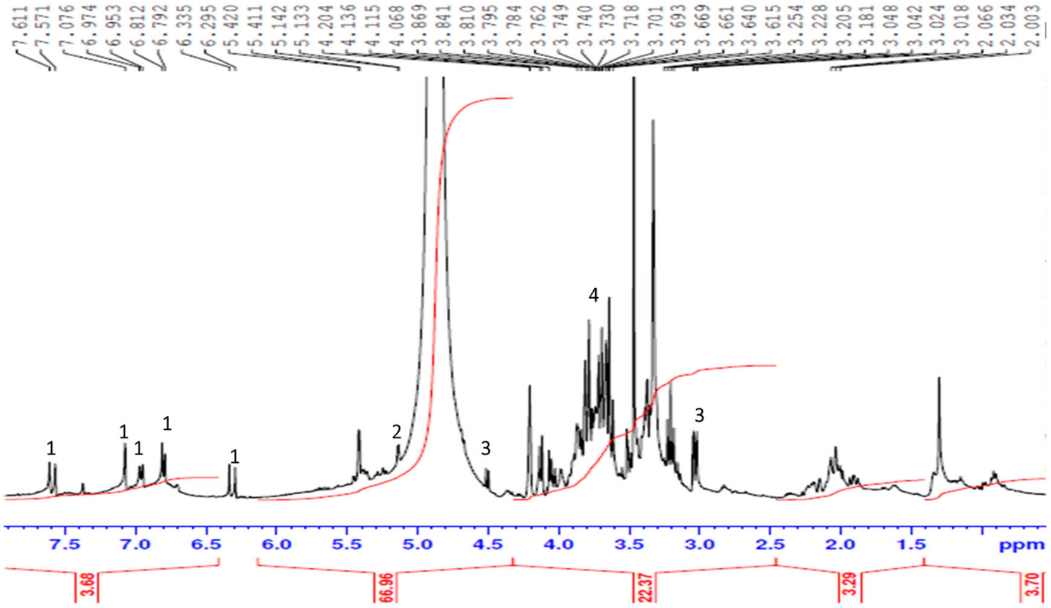

Figure S7. The representative ^{1}H-NMR spectra of fraction LM_3. 1, 5-*O*-caffeoylquinic acid; 2, α-glucose; 3, β-glucose; 4, glucose and fructose.

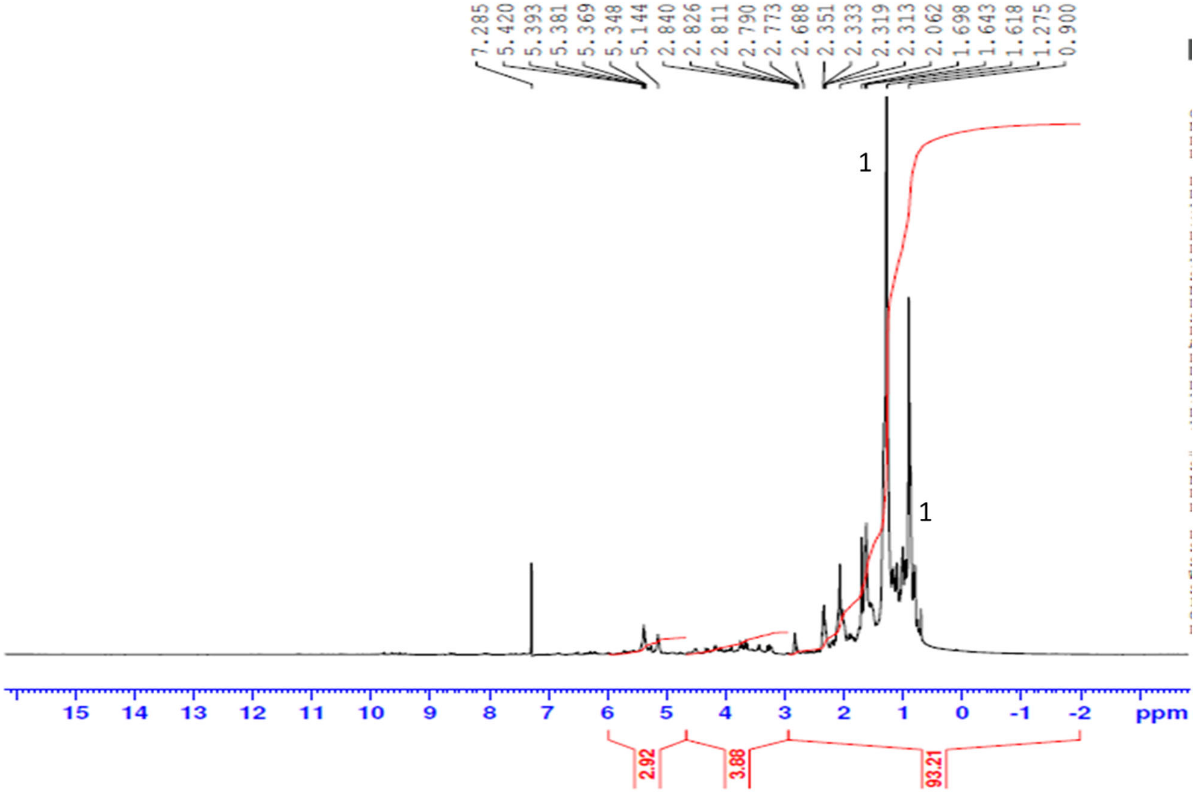

Figure S8. The representative ^{1}H-NMR spectra of LD.crude. 1, hexadecane.

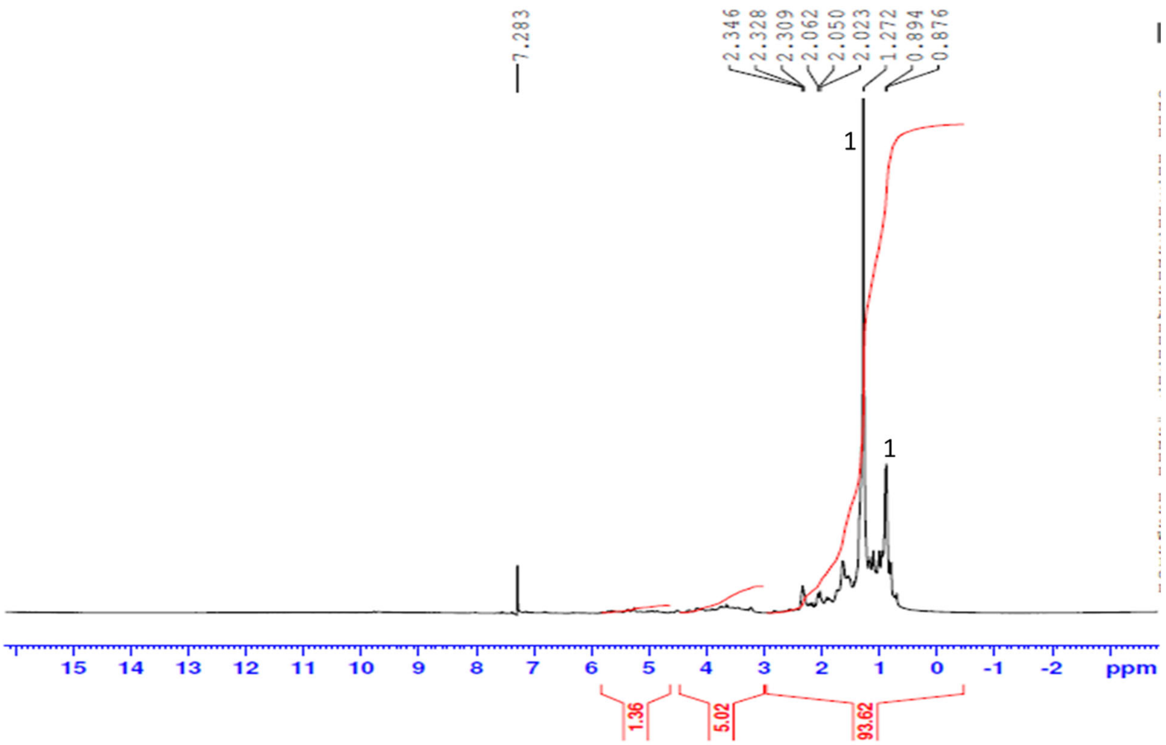

Figure S9. The representative ^{1}H-NMR spectra of fraction R_1. 1, hexadecane.

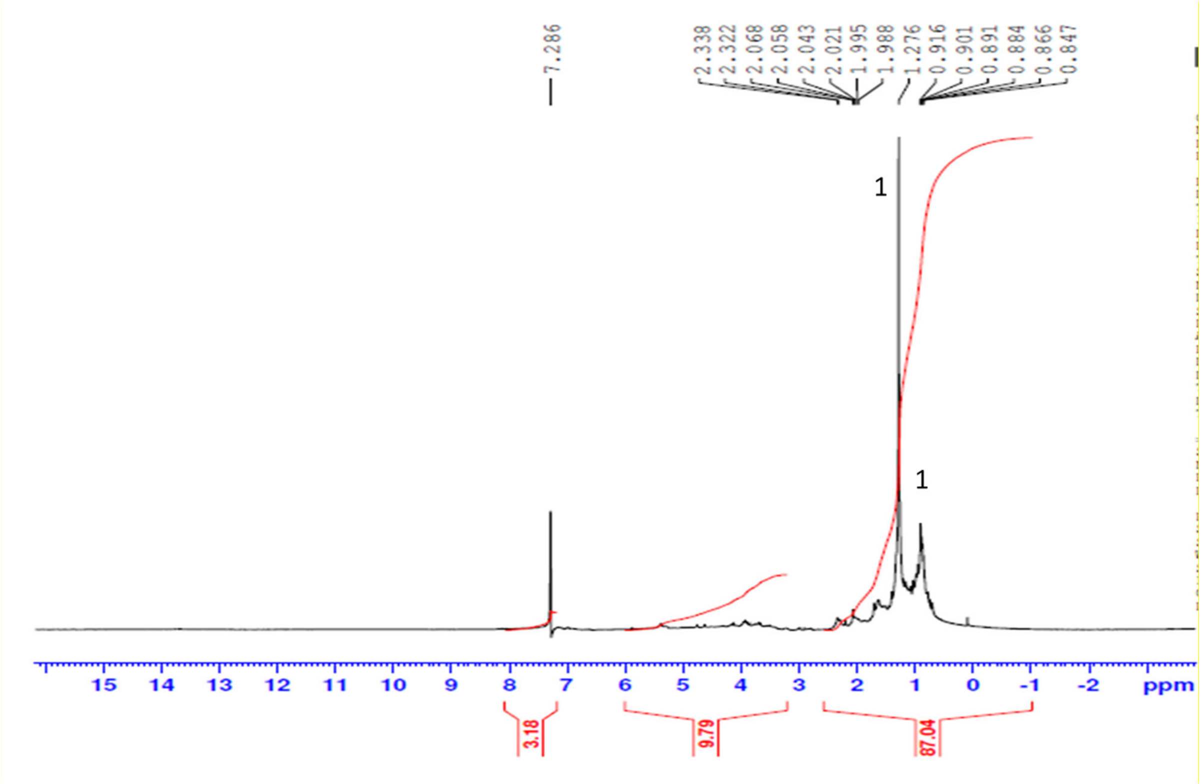

Figure S10. The representative ^{1}H-NMR spectra of fraction LD_3. 1, hexadecane.

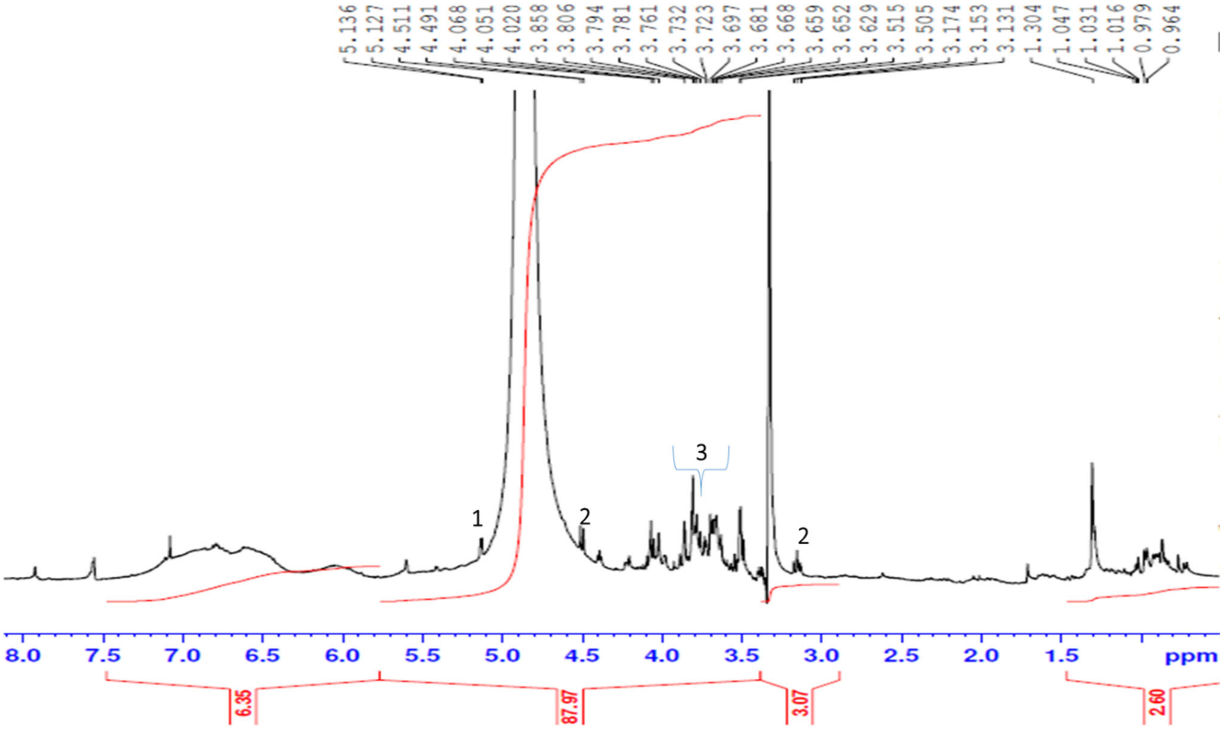

Figure S11. The representative ^{1}H-NMR spectra of S.crude. 1, α-glucose; 2, β-glucose; 3, glucose and fructose.

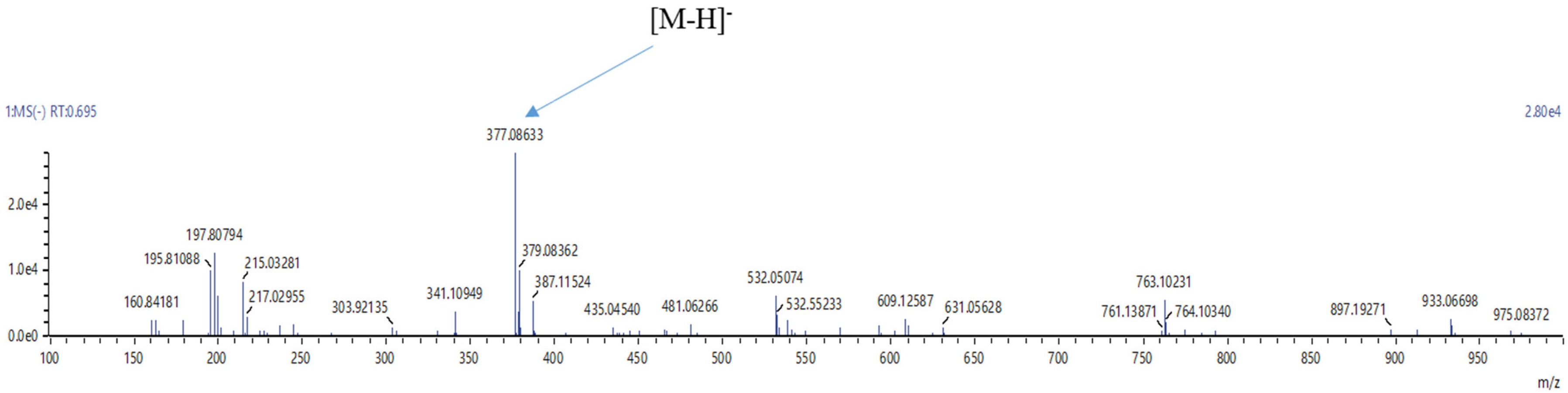

Figure S12. Molecular ion of caffeic acid derivative.

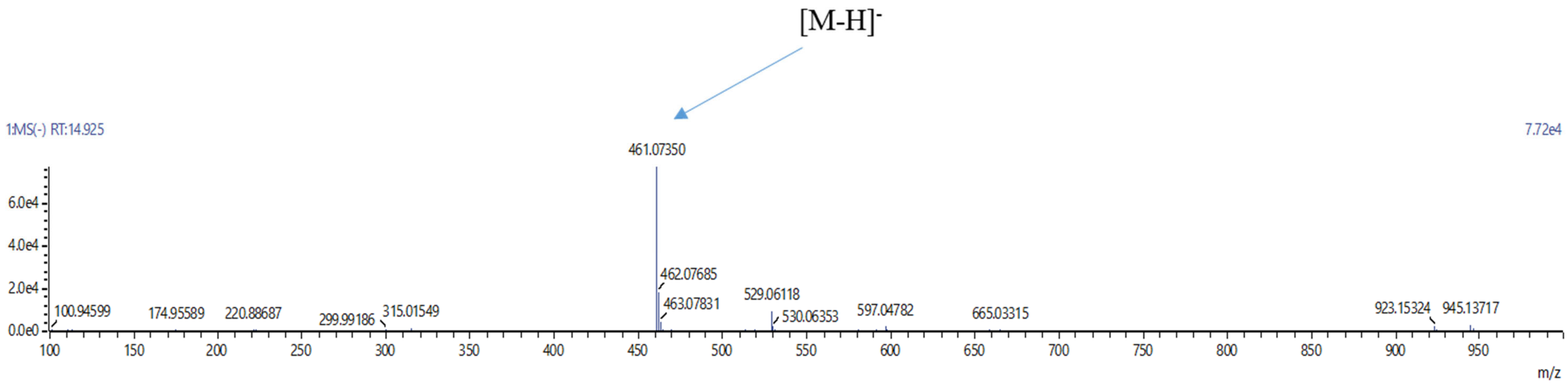

Figure S13. Molecular ion of 4'-*O*-methyellagic acid-3-*O*-α-L-rhamnopyranoside.

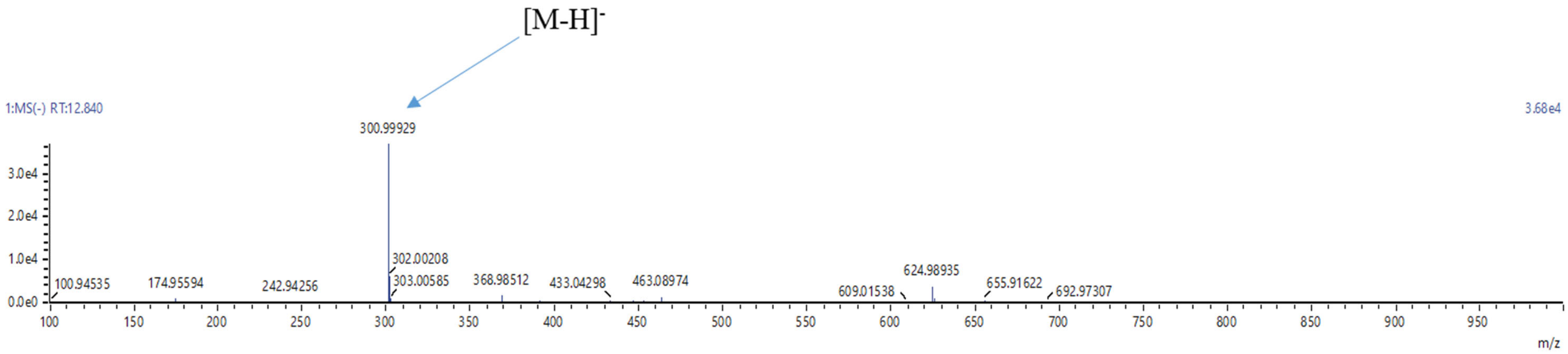

Figure S14. Molecular ion of ellagic acid.

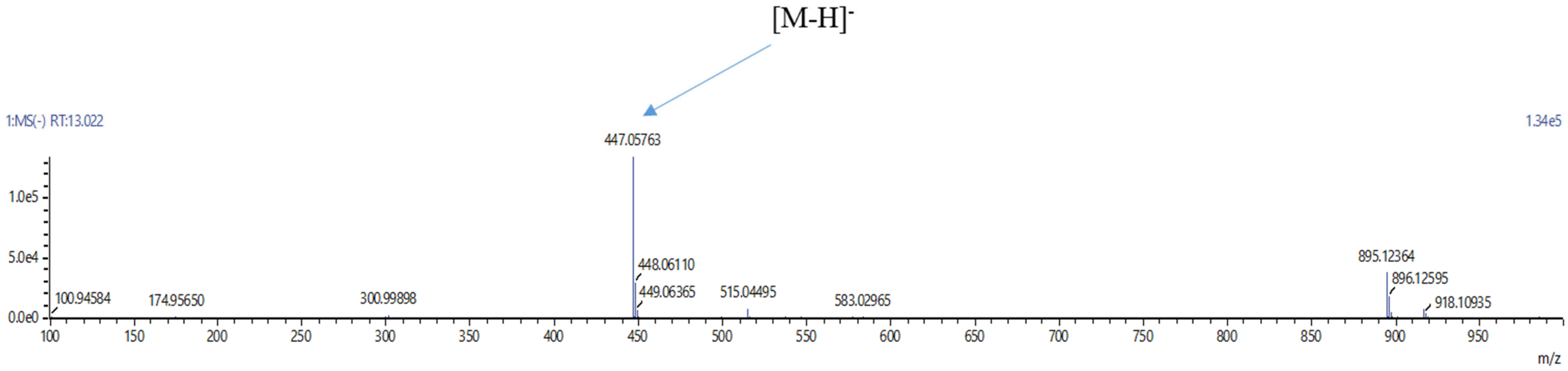

Figure S15. Molecular ion of ellagic acid-rhamnopyranoside isomer I.

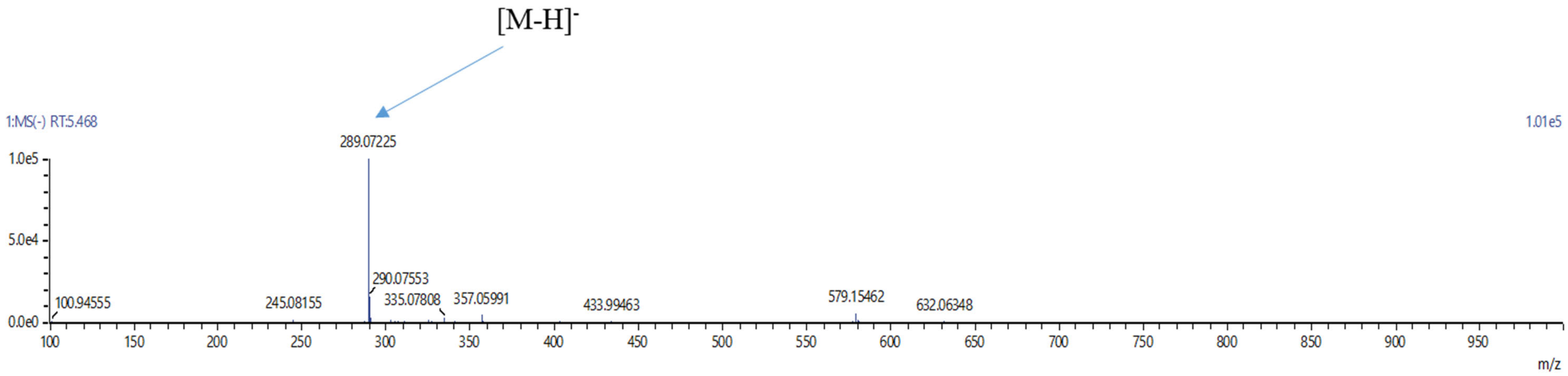

Figure S16. Molecular ion of catechin.

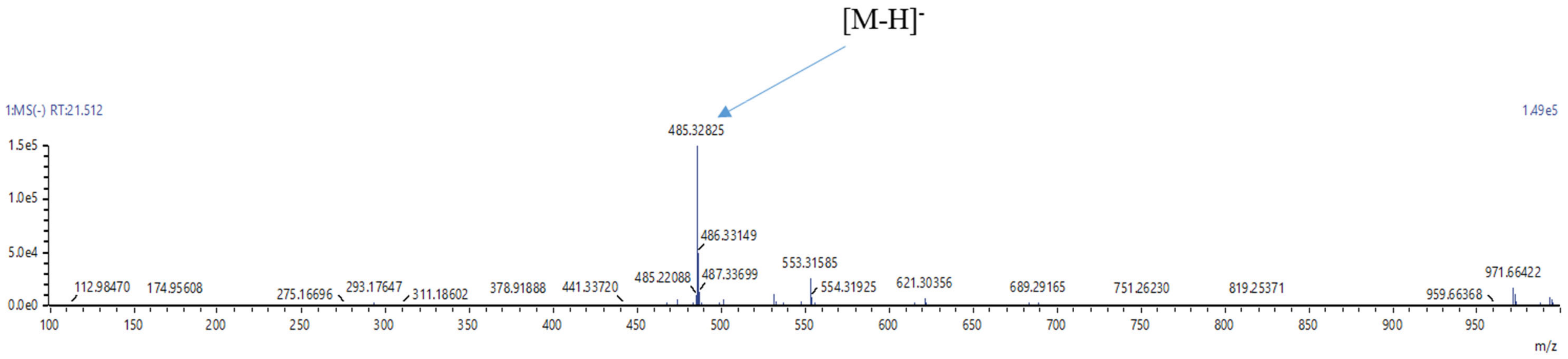

Figure S17. Molecular ion of hydroxyglycyrrhetinic acid.

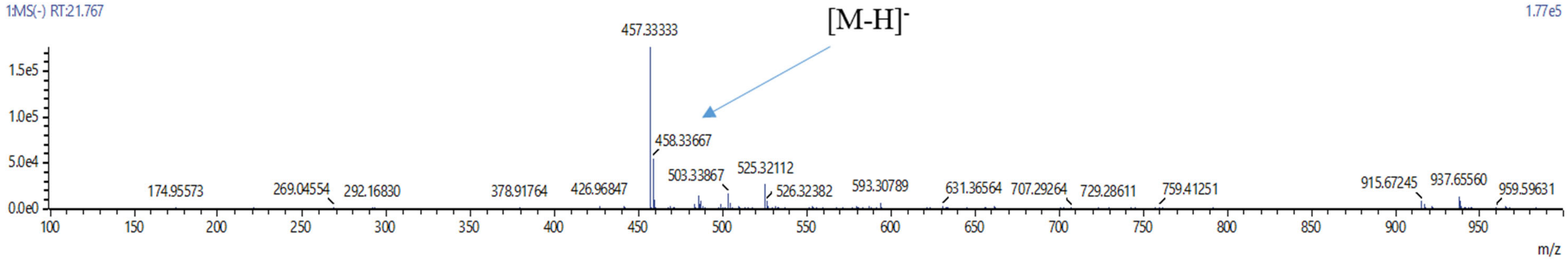

Figure S18. Molecular ion of neotigogenin acetate.

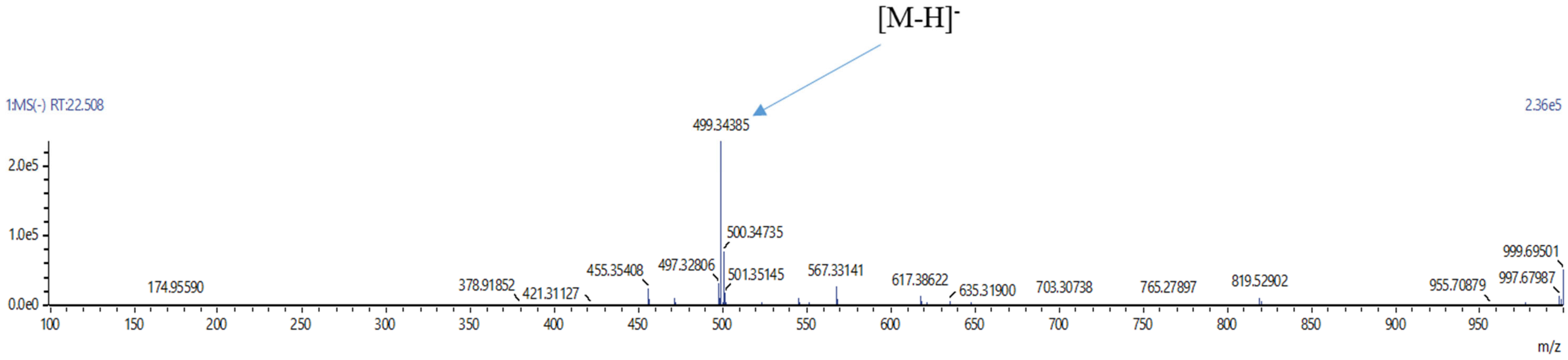

Figure S19. Molecular ion of 25-hydroxy-3-epi-dehydrotumulosic acid.

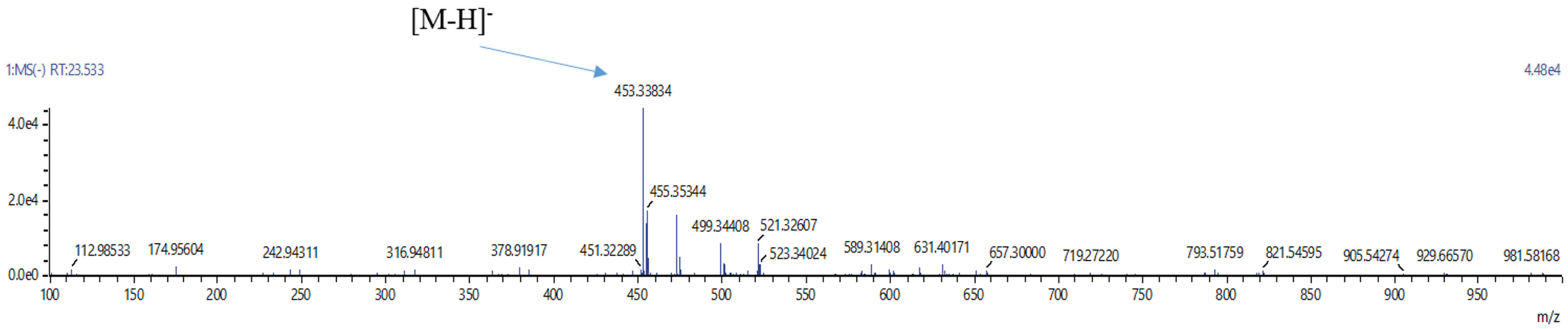

Figure S20. Molecular ion of micromeric acid.

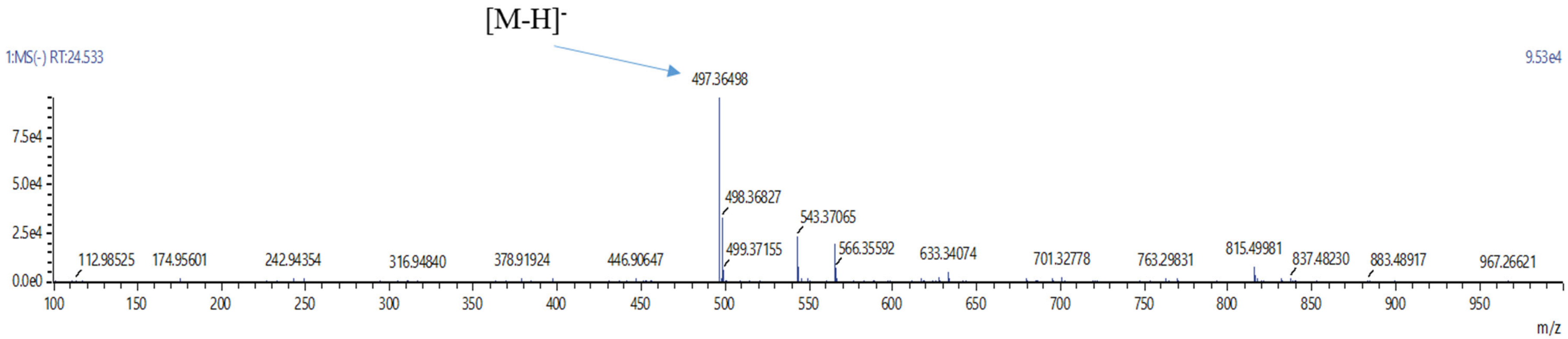

Figure S21. Molecular ion of 3-acetylursolic acid.

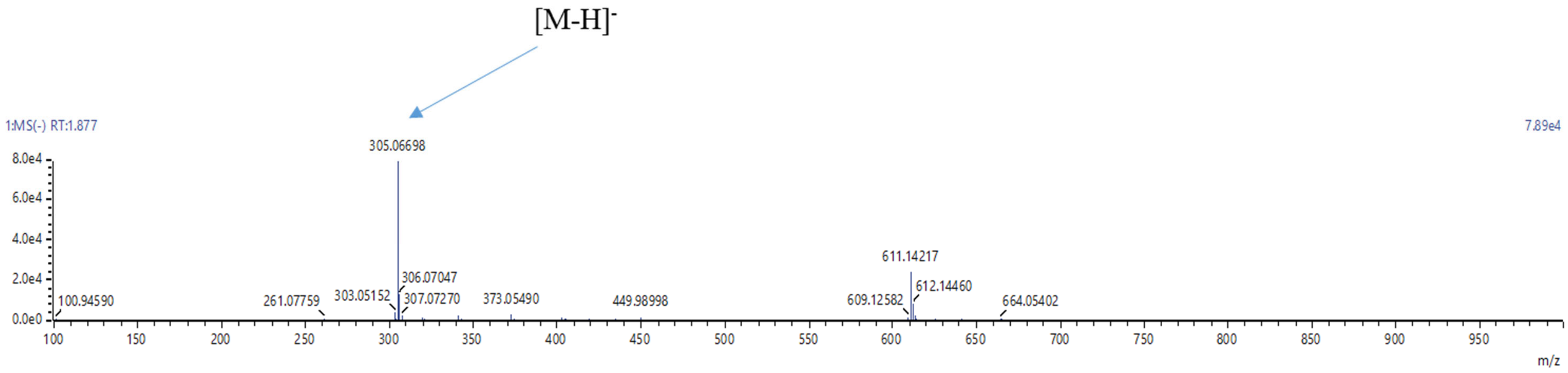

Figure S22. Molecular ion of (epi) gallocatechin.

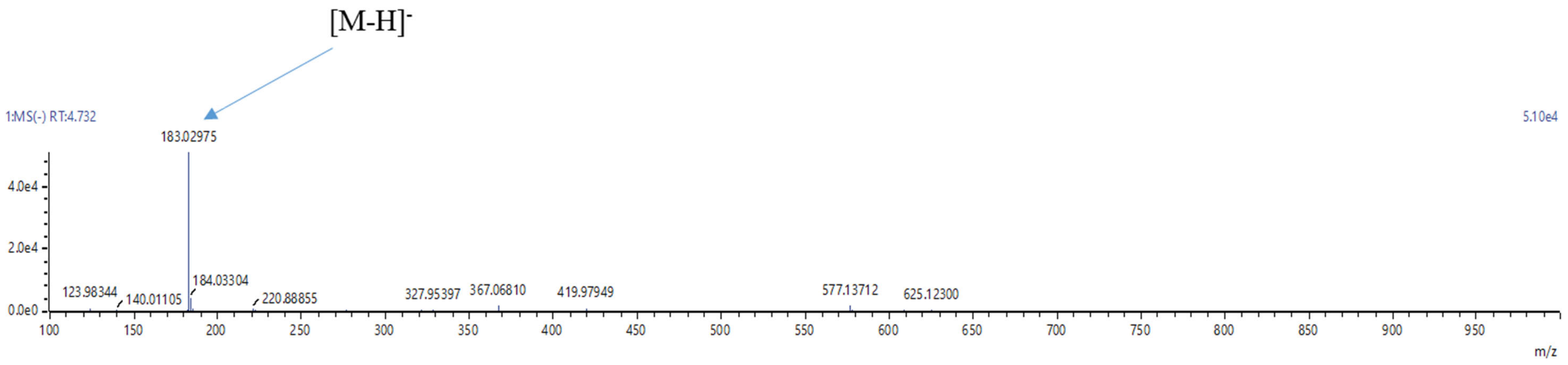

Figure S23. Molecular ion of 4-*O*-methylgallic acid.

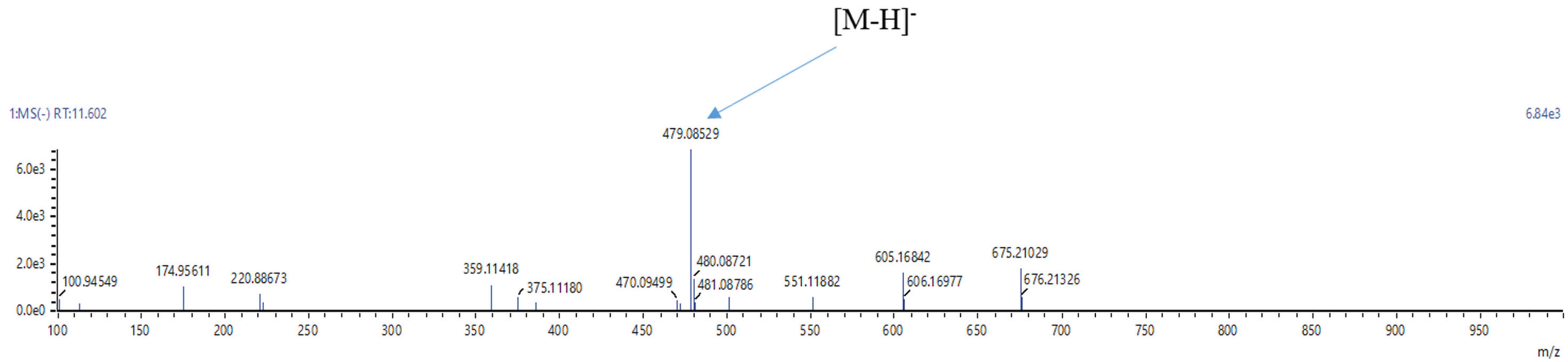

Figure S24. Molecular ion of myricetin 3-*O*-glucoside.

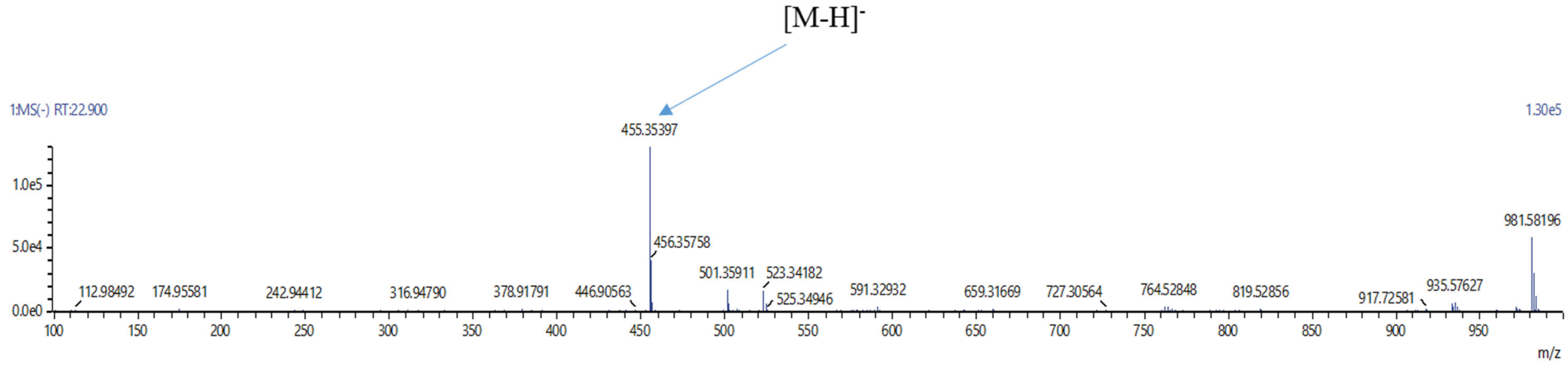

Figure S25. Molecular ion of ursolic acid.

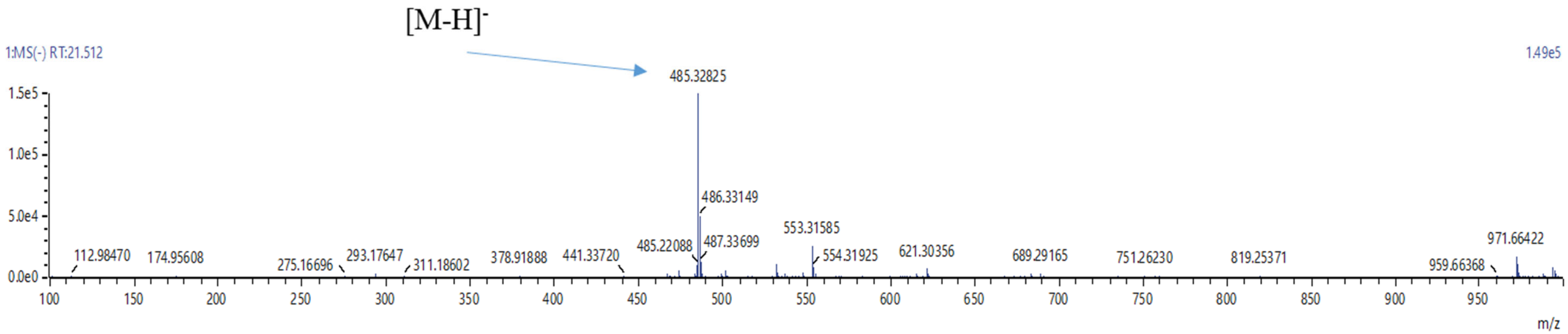

Figure S26. Molecular ion of asiatic acid.

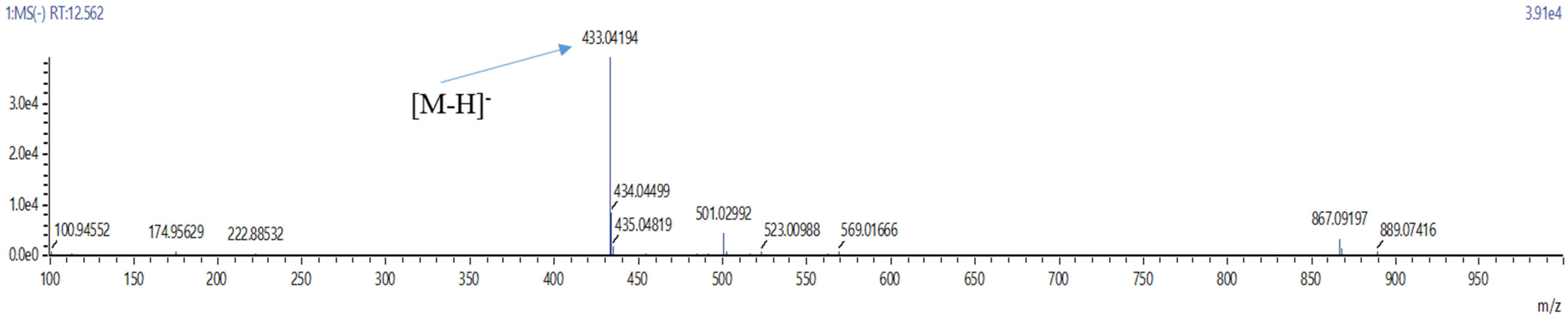

Figure S27. Molecular ion of ellagic acid pentoside.

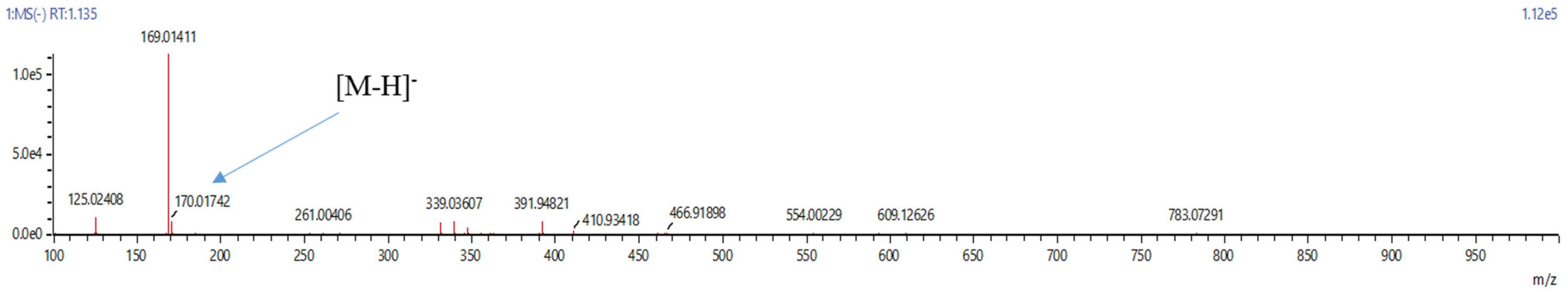

Figure S28. Molecular ion of gallic acid.

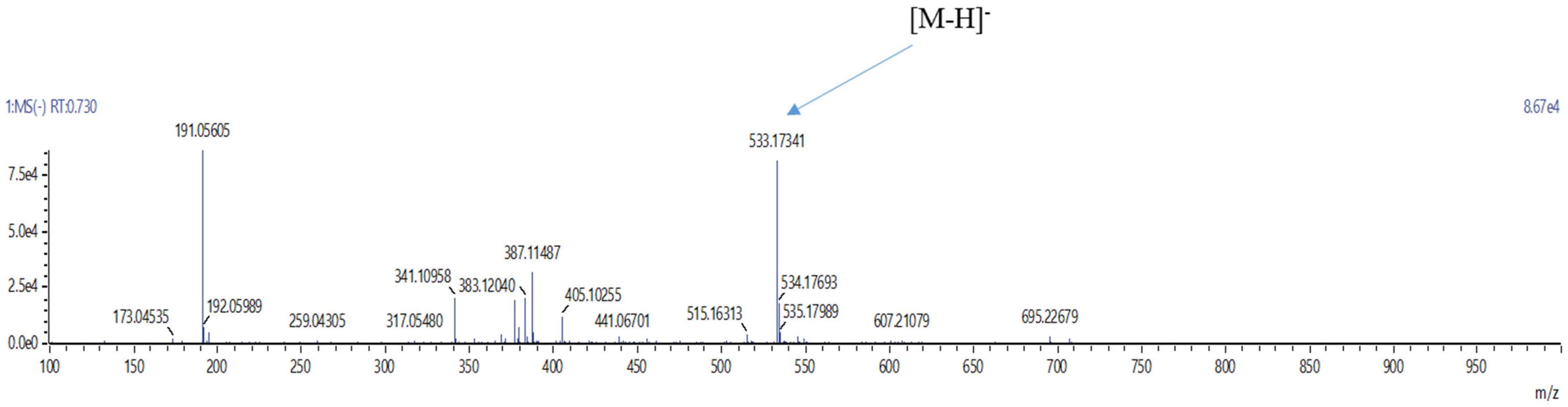

Figure S29. Molecular ion of quinic acid + hexose2.

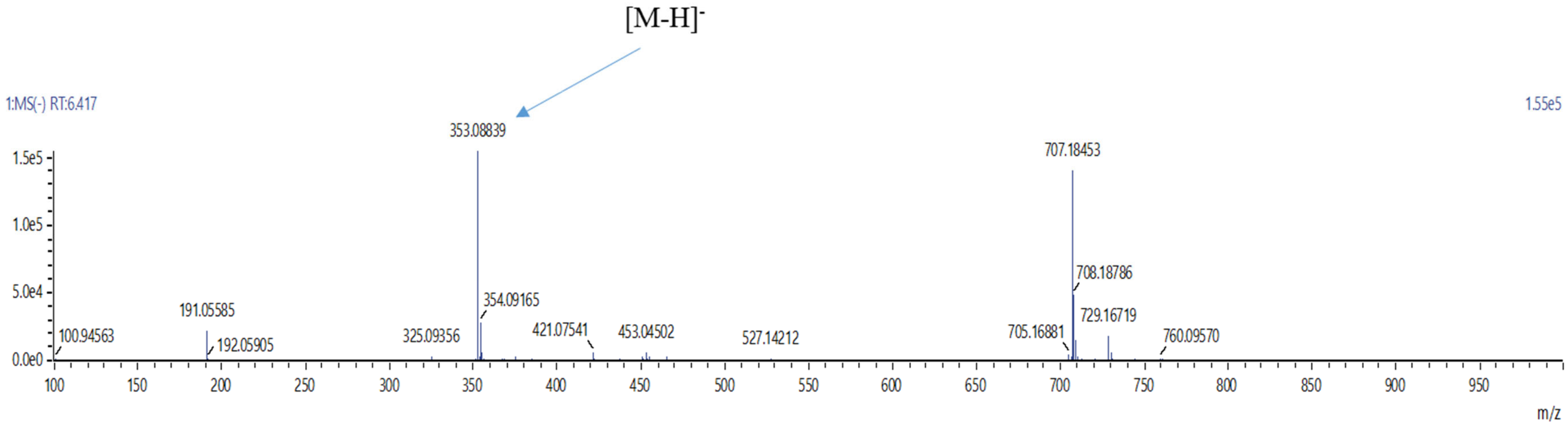

Figure S30. Molecular ion of chlorogenic acid [3.4-dihydroxycinnamoylquinic acid; 5-caffeoylquinic acid].

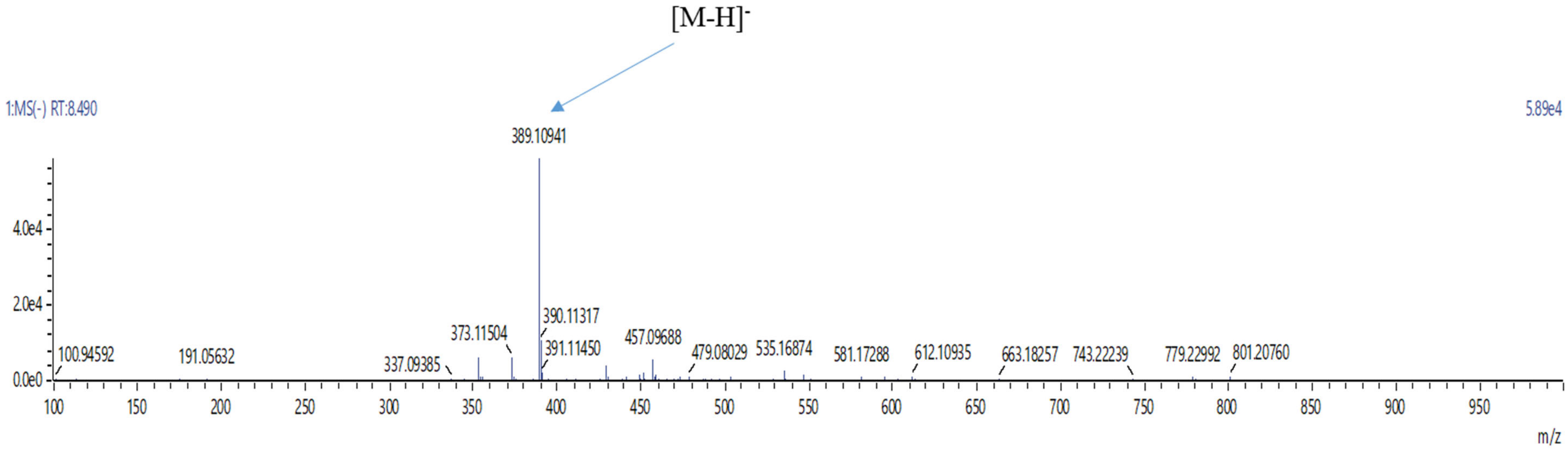

Figure S31. Molecular ion of deacetyl asperuloside acid.

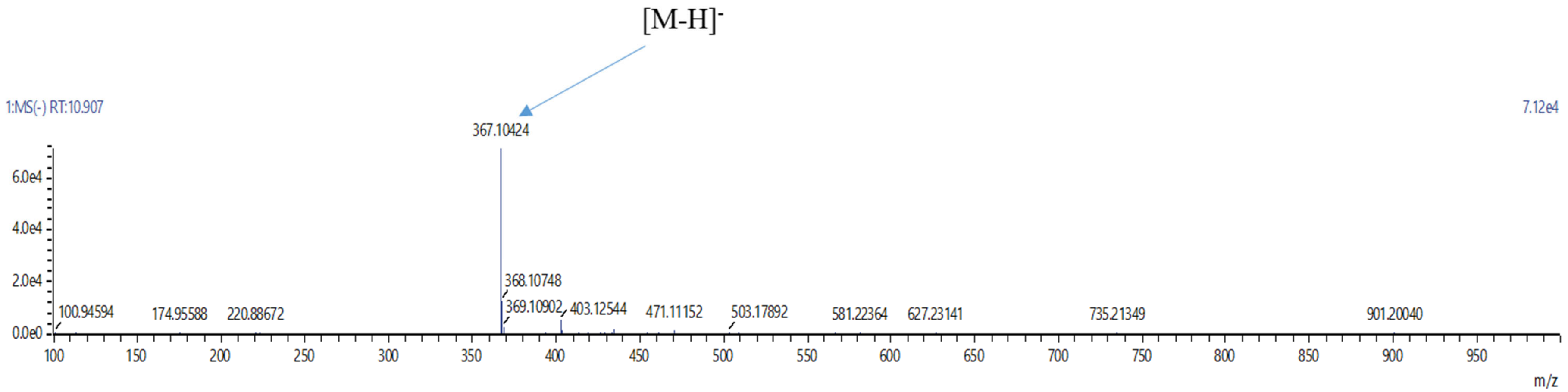

Figure S32. Molecular ion of 5-methyl caffeoylquinic acid.

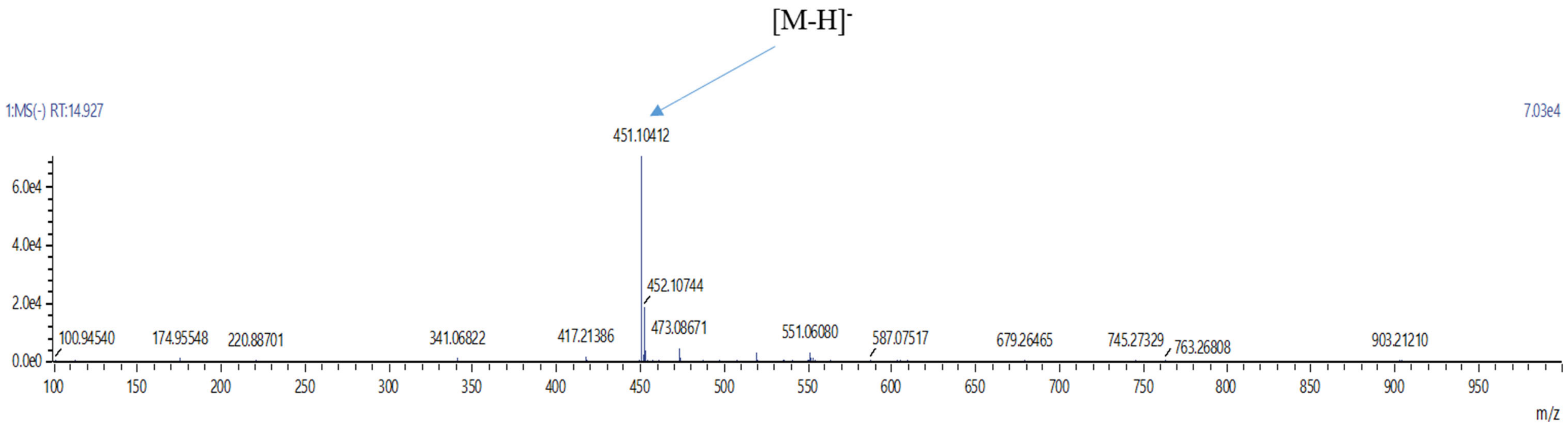

Figure S33. Molecular ion of cinchonain I isomer.

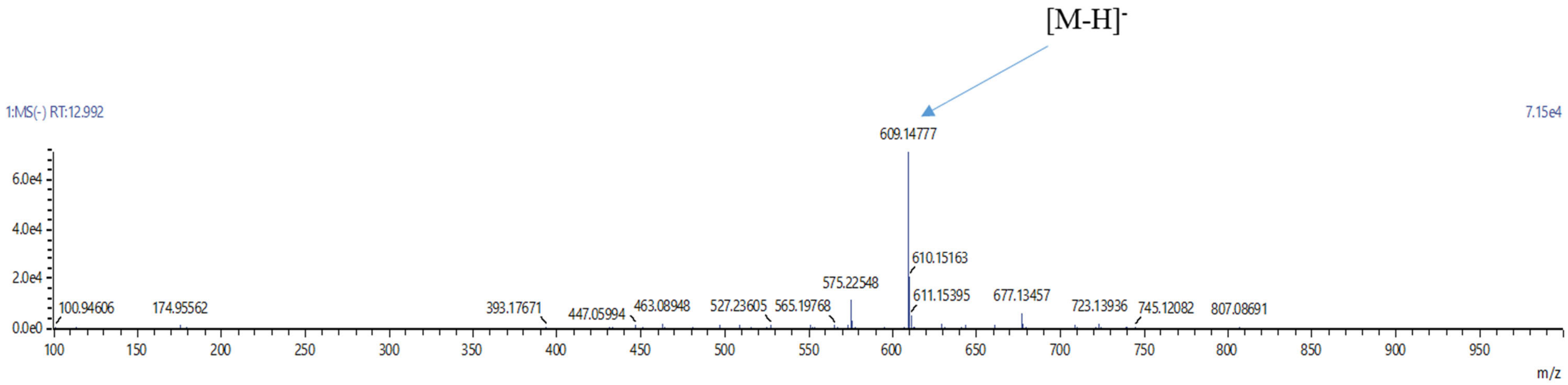

Figure S34. Molecular ion of rutin.

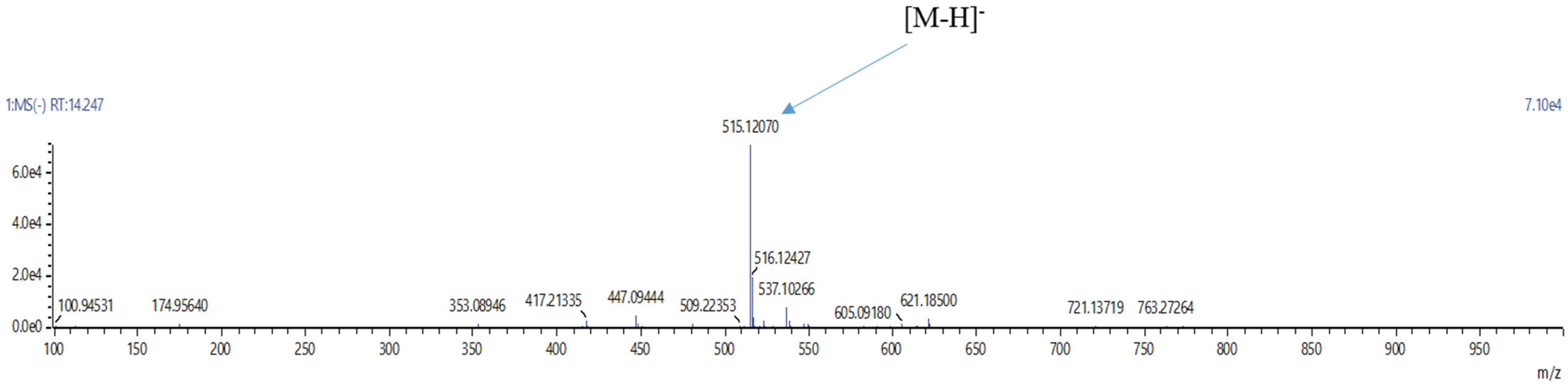

Figure S35. Molecular ion of di-*O*-caffeoylquinic acid.

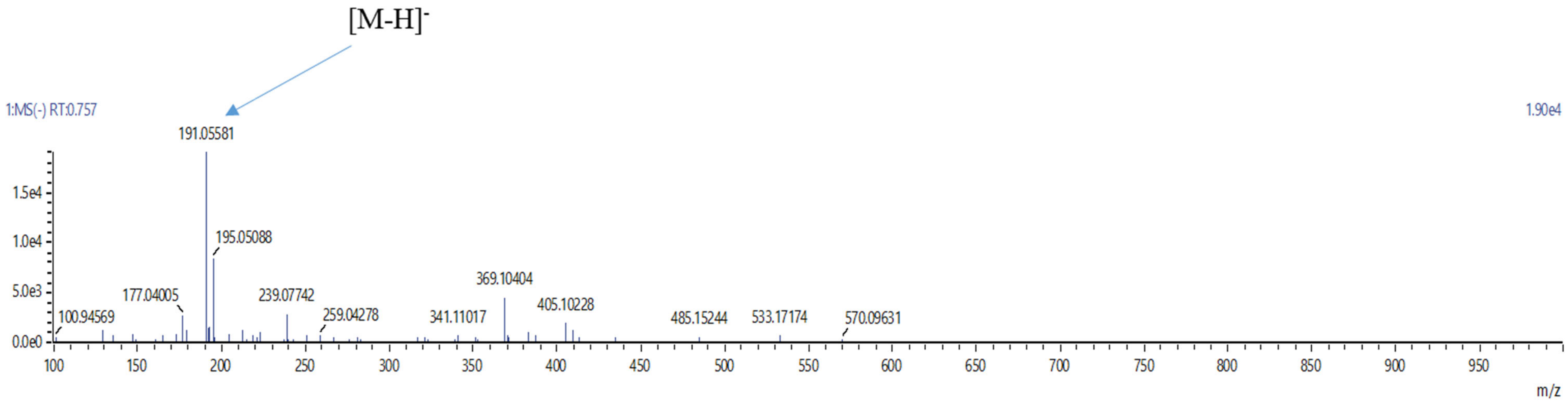

Figure S36. Molecular ion of quinic acid.

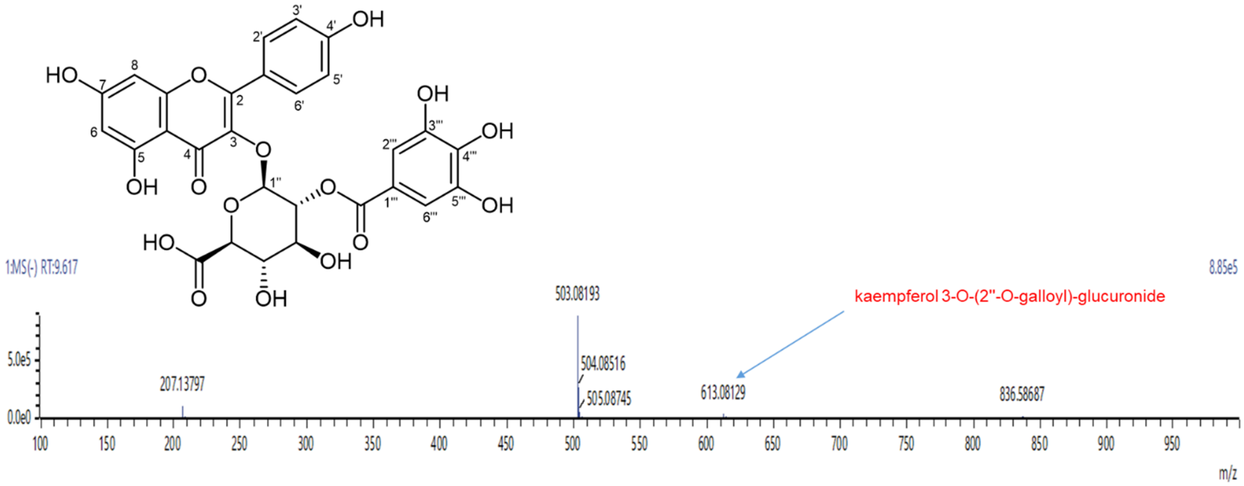

Figure S37. Mass spectrum of kaempferol 3-*O*-(2''-*O*-galloyl)-glucuronide (**1**) [1].

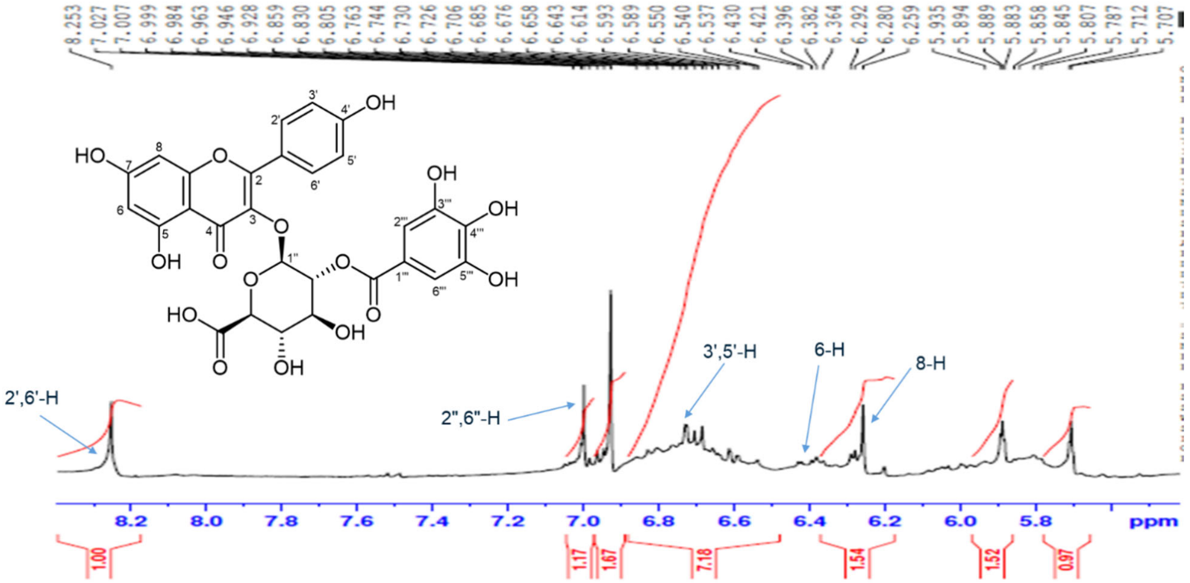

Figure S38. Expanded ^{1}H-NMR spectrum of kaempferol 3-*O*-(2''-*O*-galloyl)-glucuronide (**1**) [1].

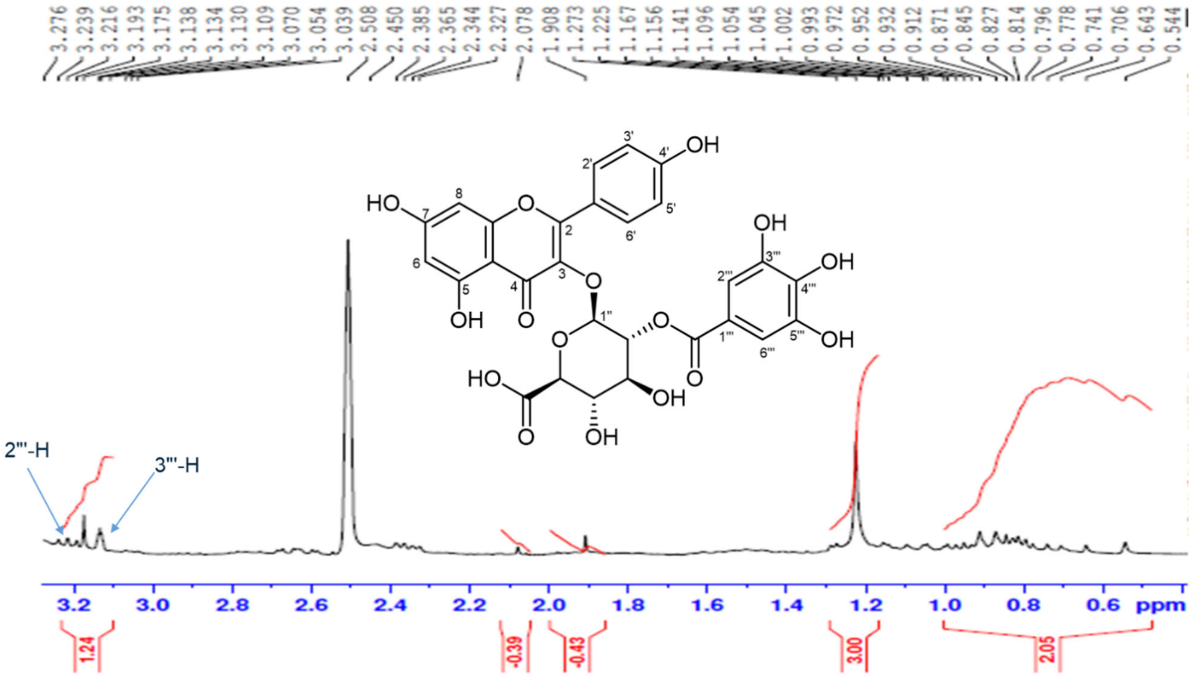

Figure S39. Expanded ^{1}H-NMR spectrum of kaempferol 3-*O*-(2''-*O*-galloyl)-glucuronide (**1**) [1].

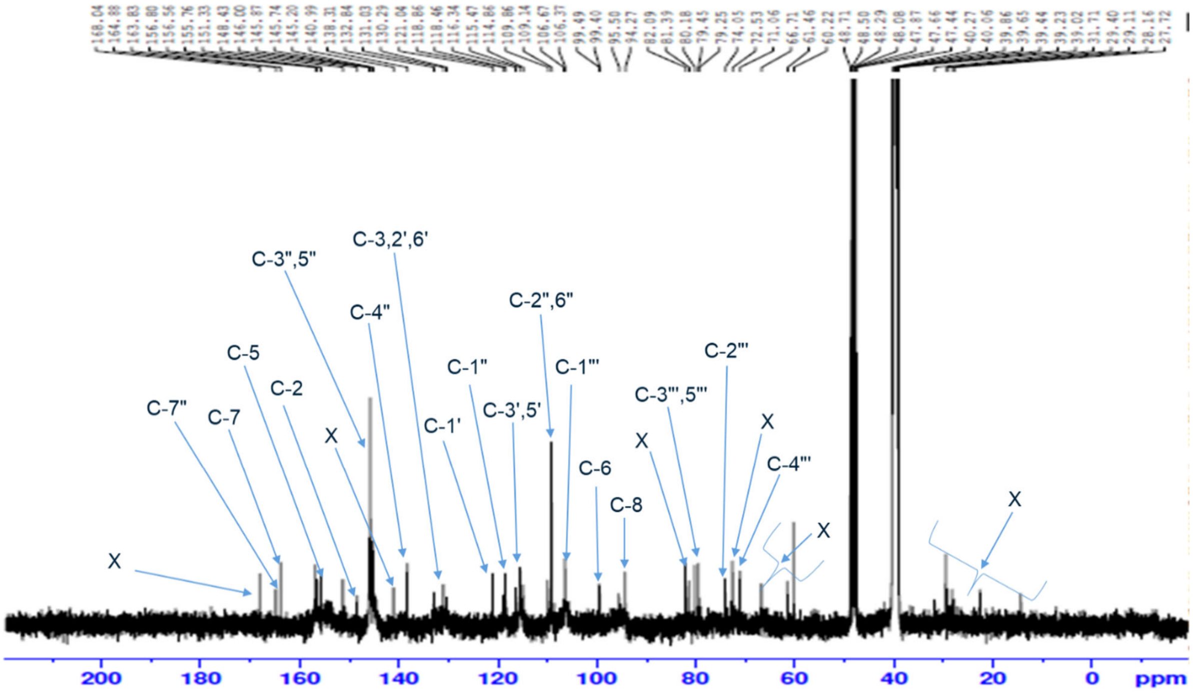

Figure S40. ^{13}C-NMR spectrum of kaempferol 3-*O*-(2''-*O*-galloyl)-glucuronide (**1**) [1].

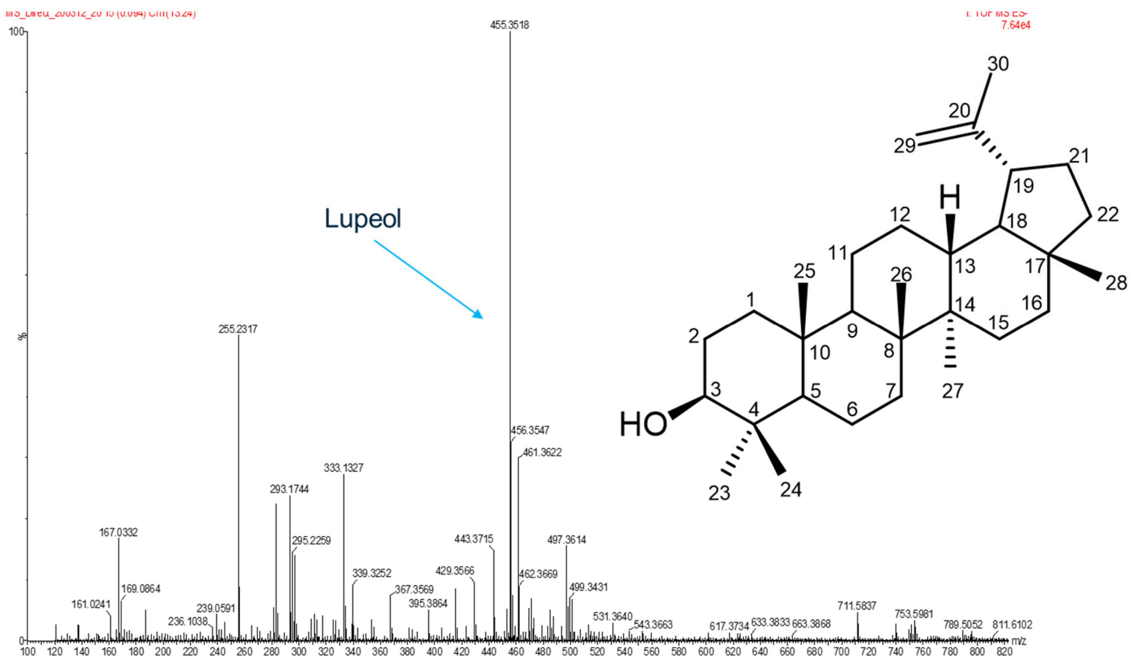

Figure S41. Mass spectrum of lupeol (**2**) [2].

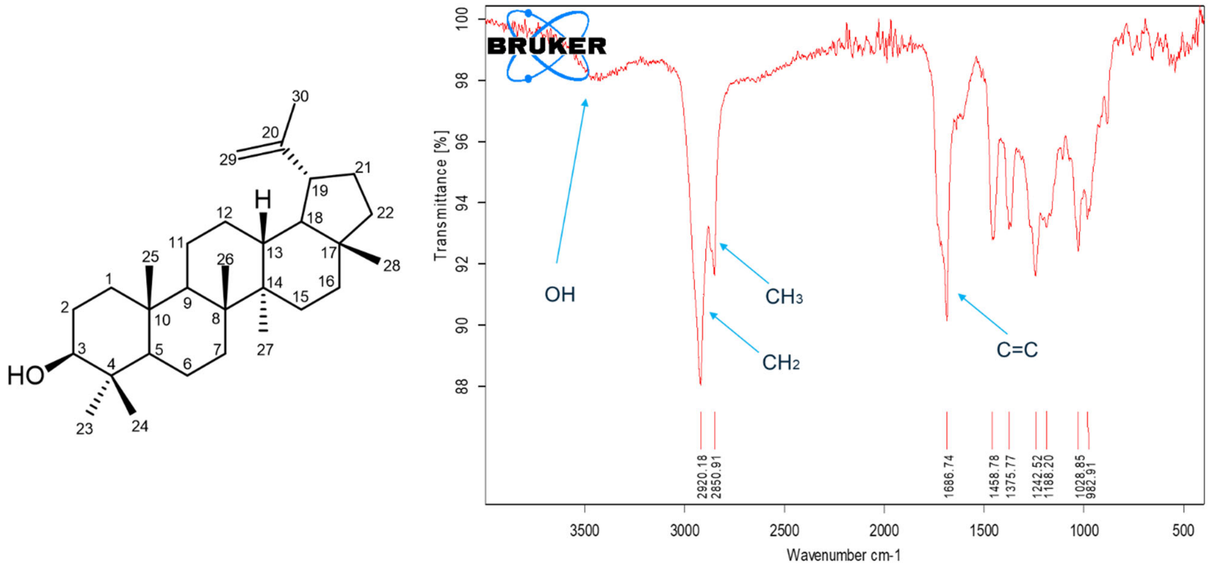

Figure S42. IR spectrum of lupeol (**2**) [2].

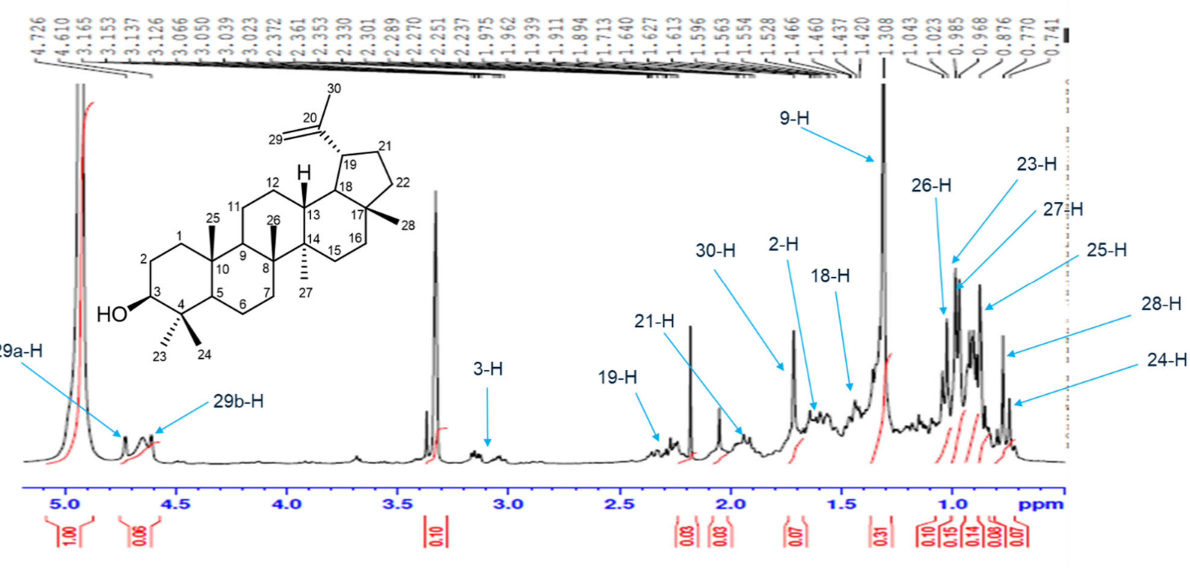

Figure S43. Expanded ^{1}H-NMR spectrum of lupeol (**2**) [2].

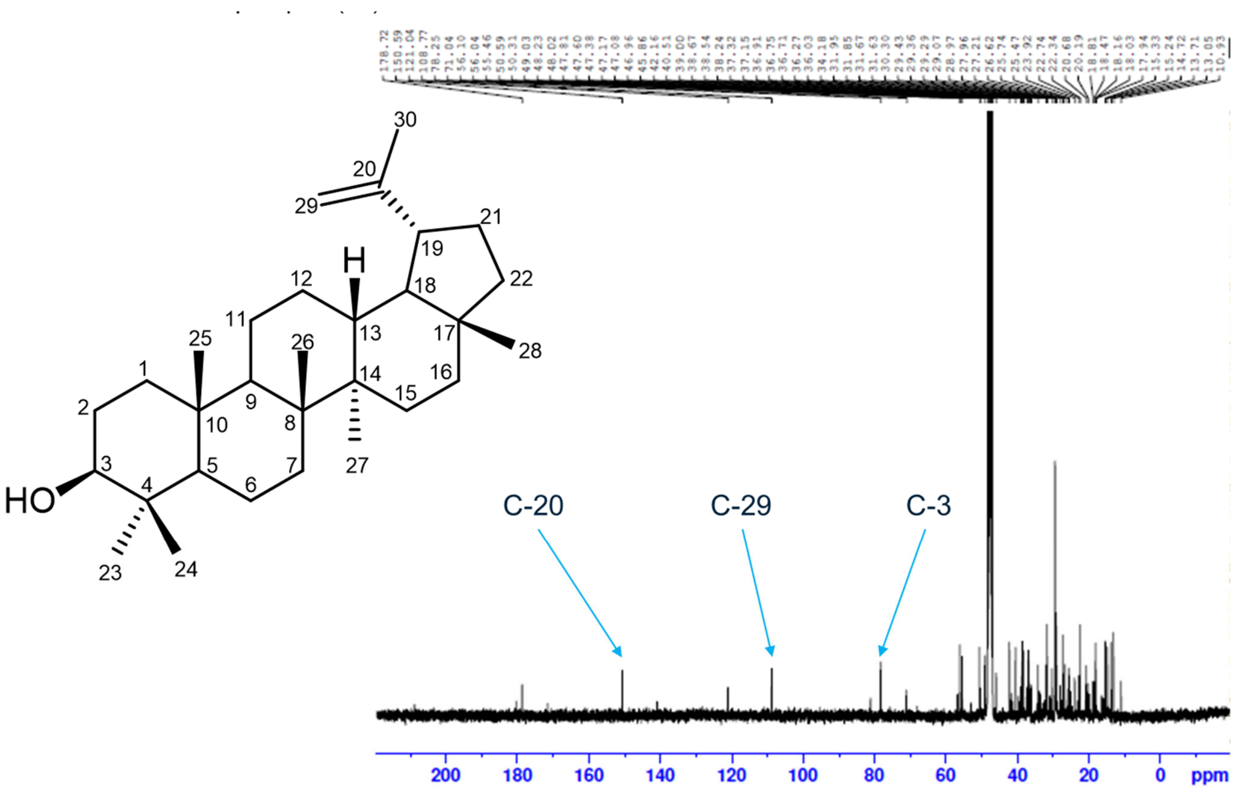

Figure S44. ^{13}C-NMR spectrum of lupeol (**2**) [2].

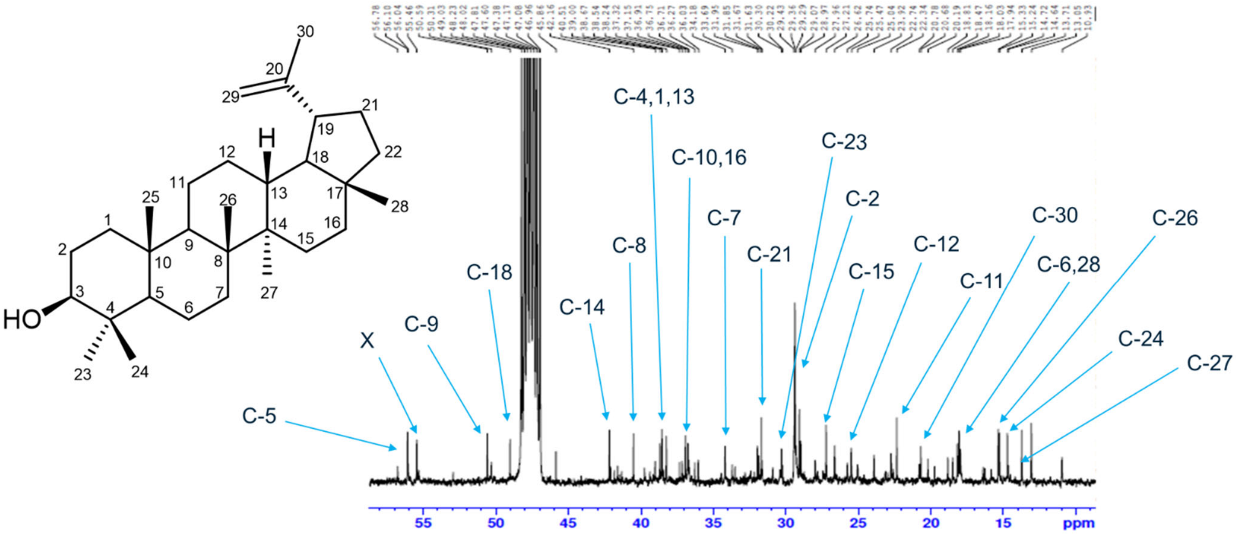

Figure S45. Expanded ^{13}C-NMR spectrum of lupeol (**2**) [2].

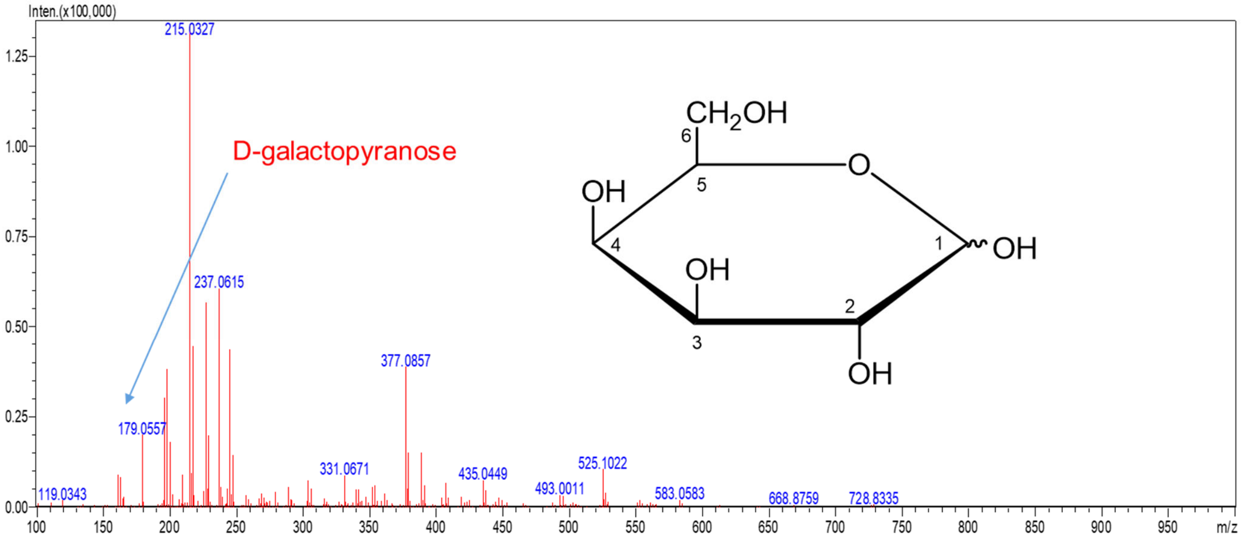

Figure S46. Mass spectrum of D-galactopyranose (**3**) [3].

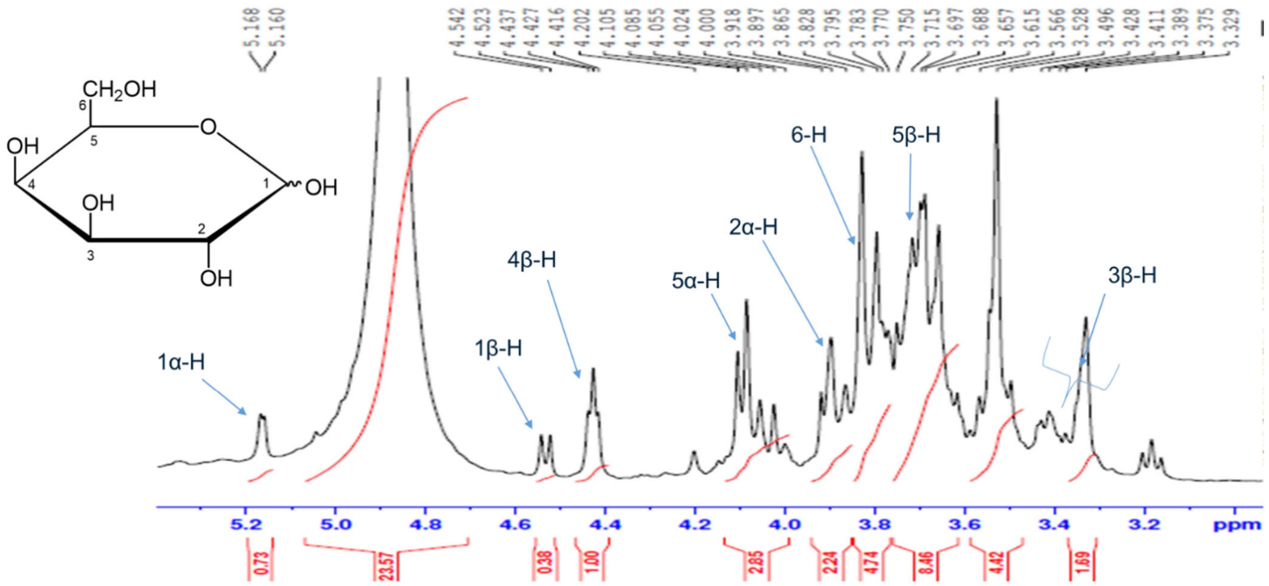

Figure S47. Expanded ^{1}H-NMR spectrum of D-galactopyranose (**3**) [3].

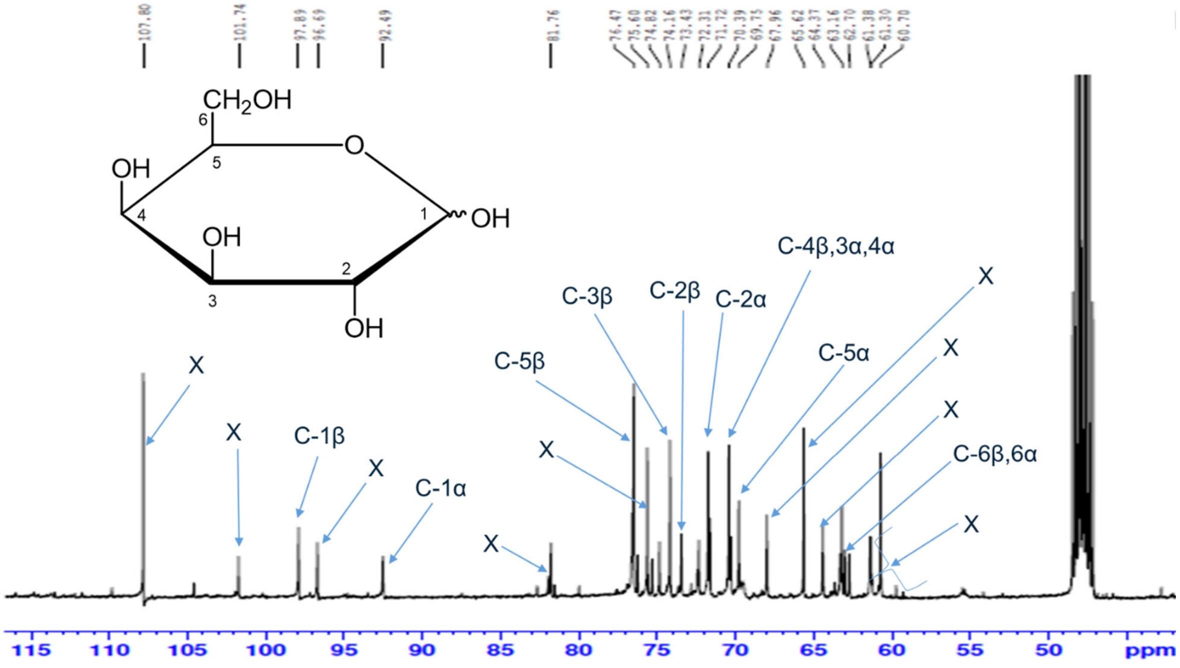

Figure S48. ^{13}C-NMR spectrum of D-galactopyranose (**3**) [3].

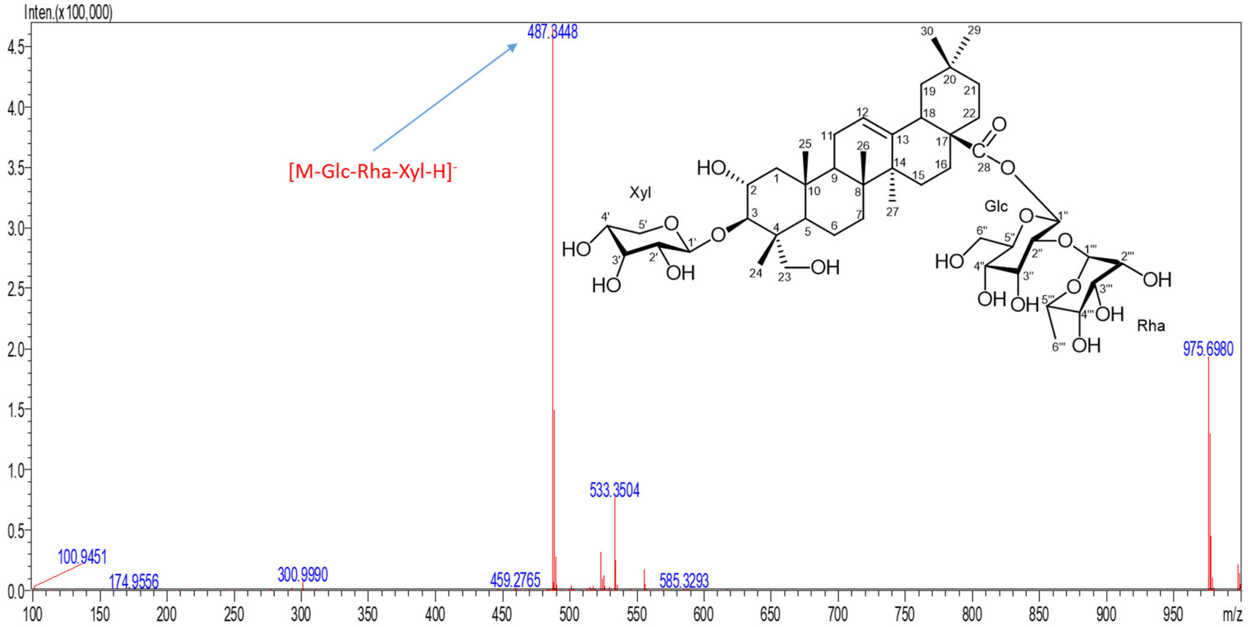

Figure S49. Mass spectrum of bodinioside Q (**4**) [4].

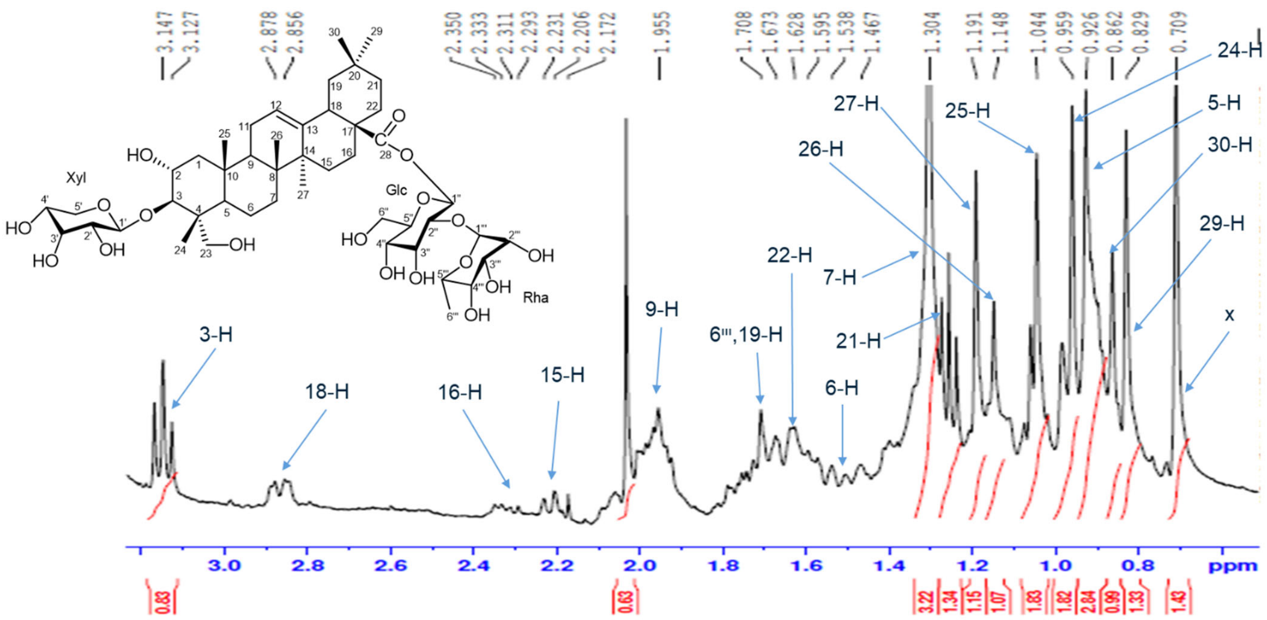

Figure S50. Expanded ^{1}H-NMR spectrum of bodinioside Q (**4**) [4].

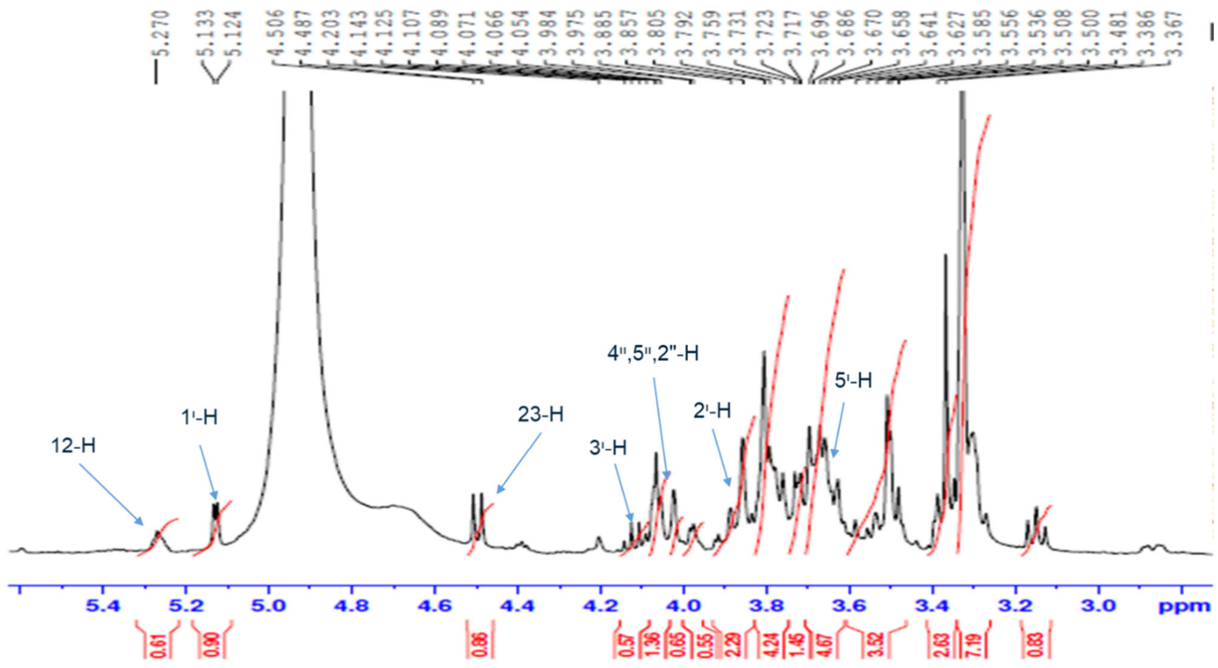

Figure S51. Expanded ¹H-NMR spectrum of bodinioside Q (**4**) [4].

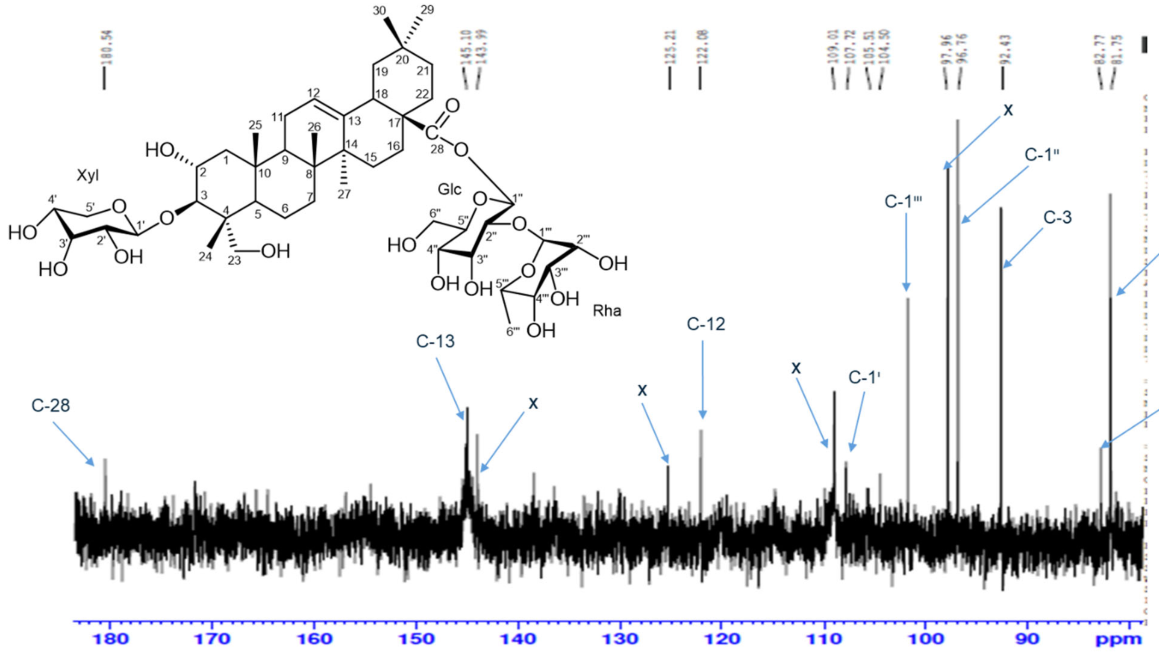

Figure S52. ¹³C-NMR spectrum of bodinioside Q (**4**) [4].

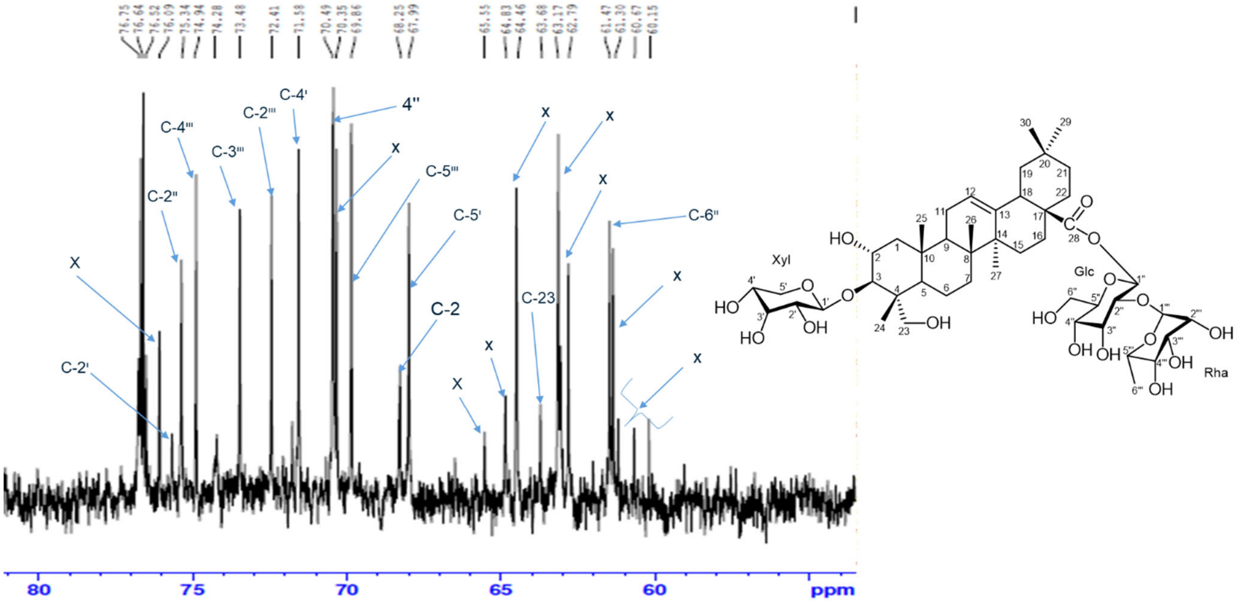

Figure S53. ^{13}C-NMR spectrum of bodinioside Q (**4**) [4].

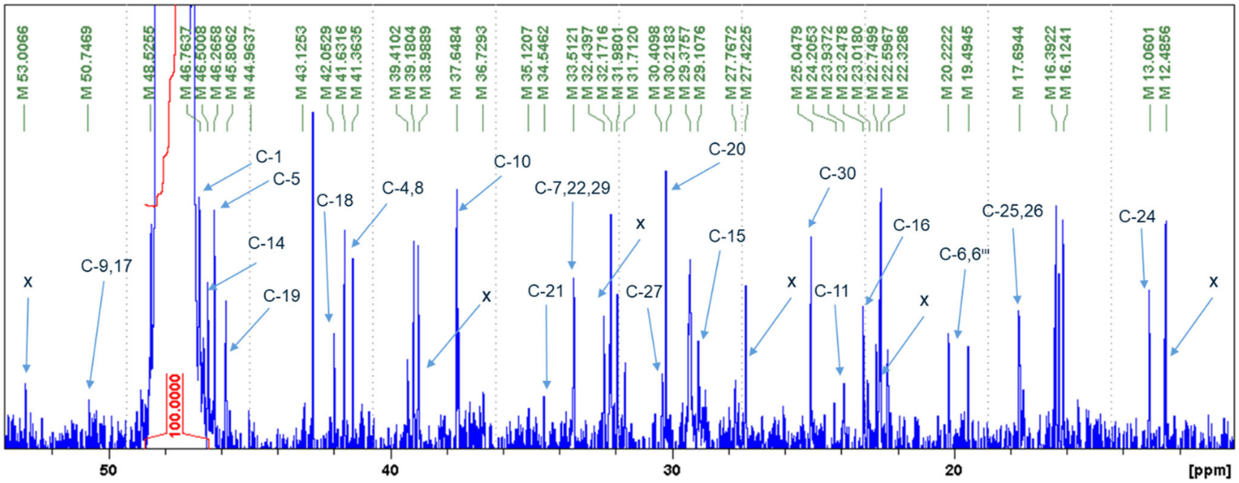

Figure S54. ^{13}C-NMR spectrum of bodinioside Q (**4**) [4].

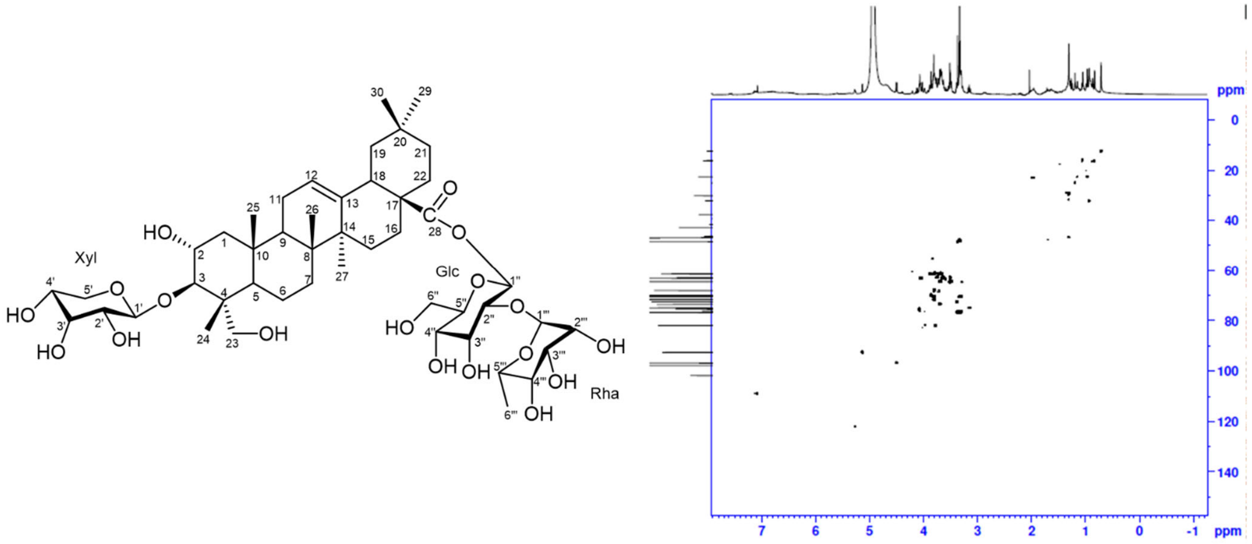

Figure S55. HSQC spectrum of bodinioside Q (**4**) [4].

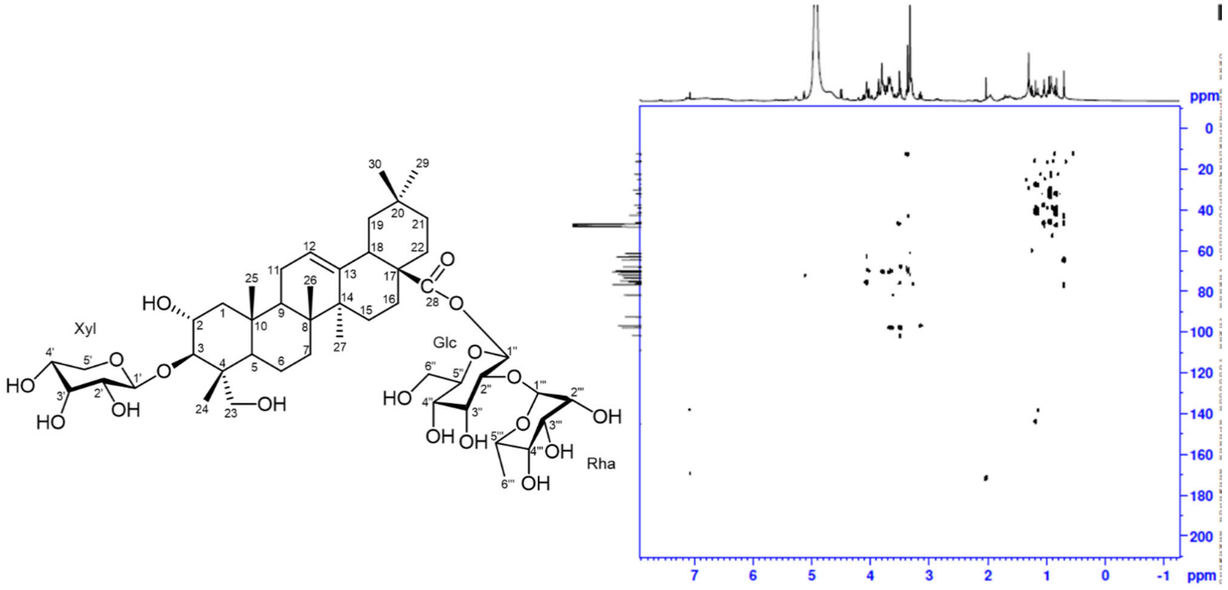

Figure S56. HMBC spectrum of bodinioside Q (**4**) [4].

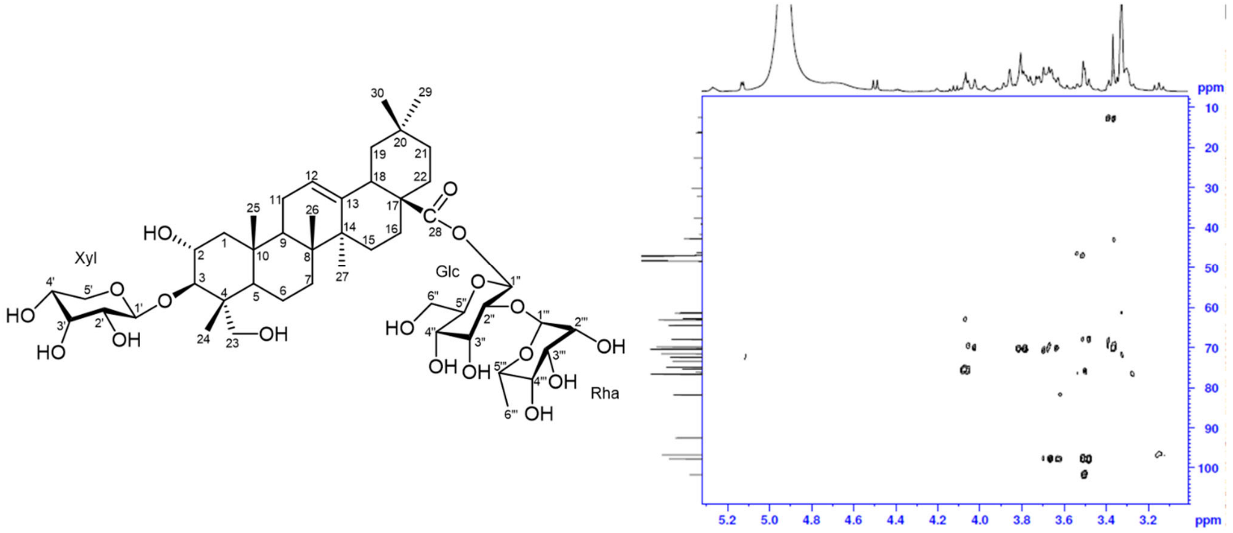

Figure S57. Expanded HMBC spectrum of bodinioside Q (**4**) [4].

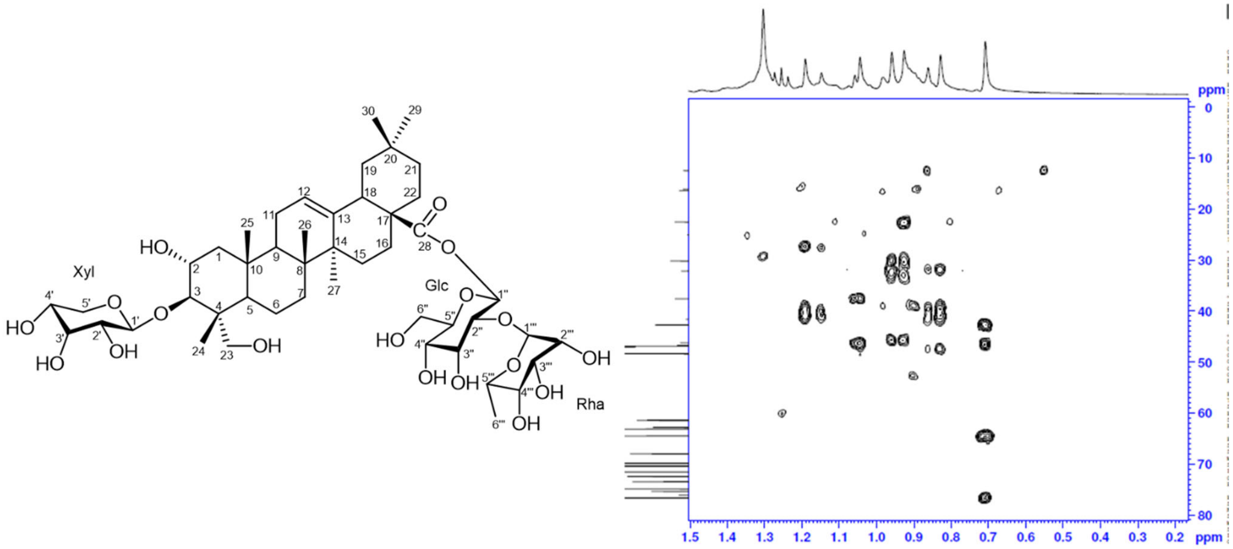

Figure S58. Expanded HMBC spectrum of bodinioside Q (**4**) [4].

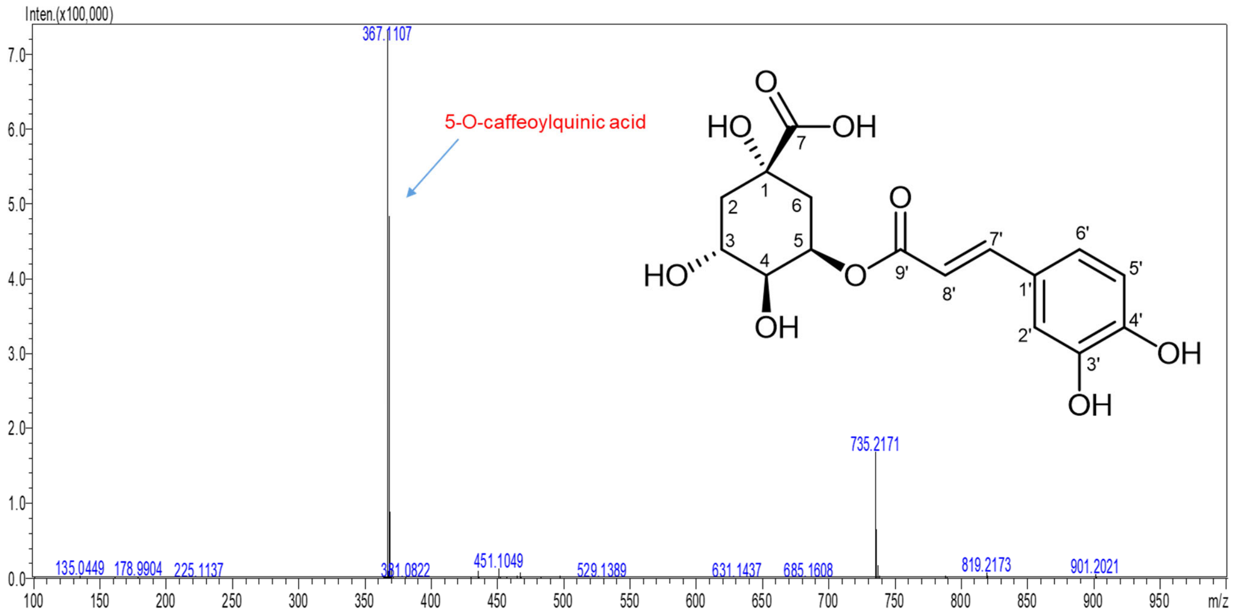

Figure S59. Mass spectrum of 5-*O*-caffeoylquinic acid (**5**) [5].

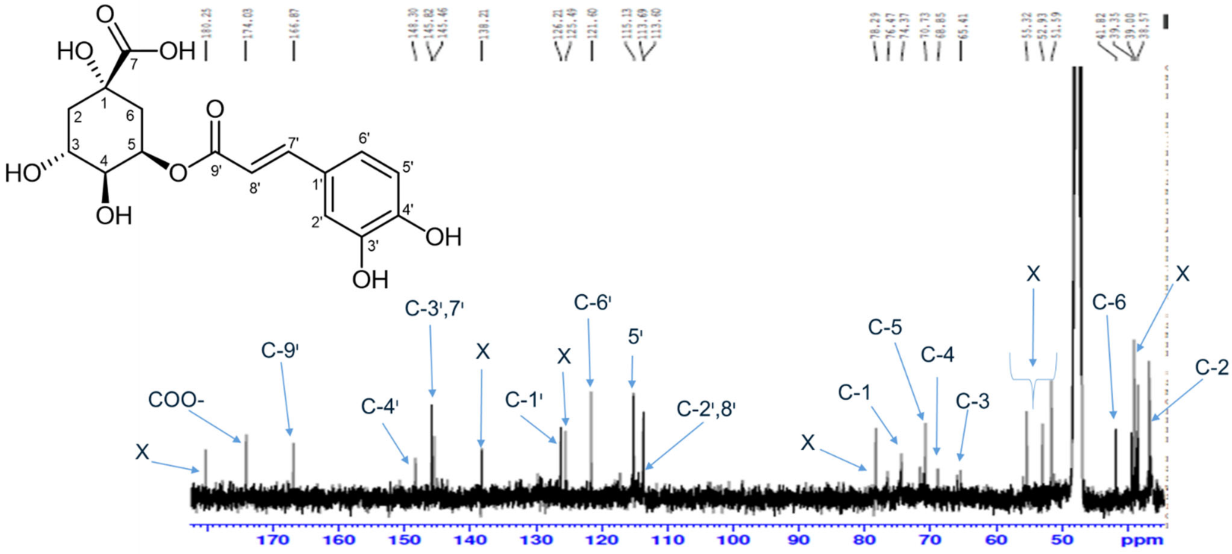

Figure S60. ^{13}C-NMR spectrum of 5-*O*-caffeoylquinic acid (**5**) [5].

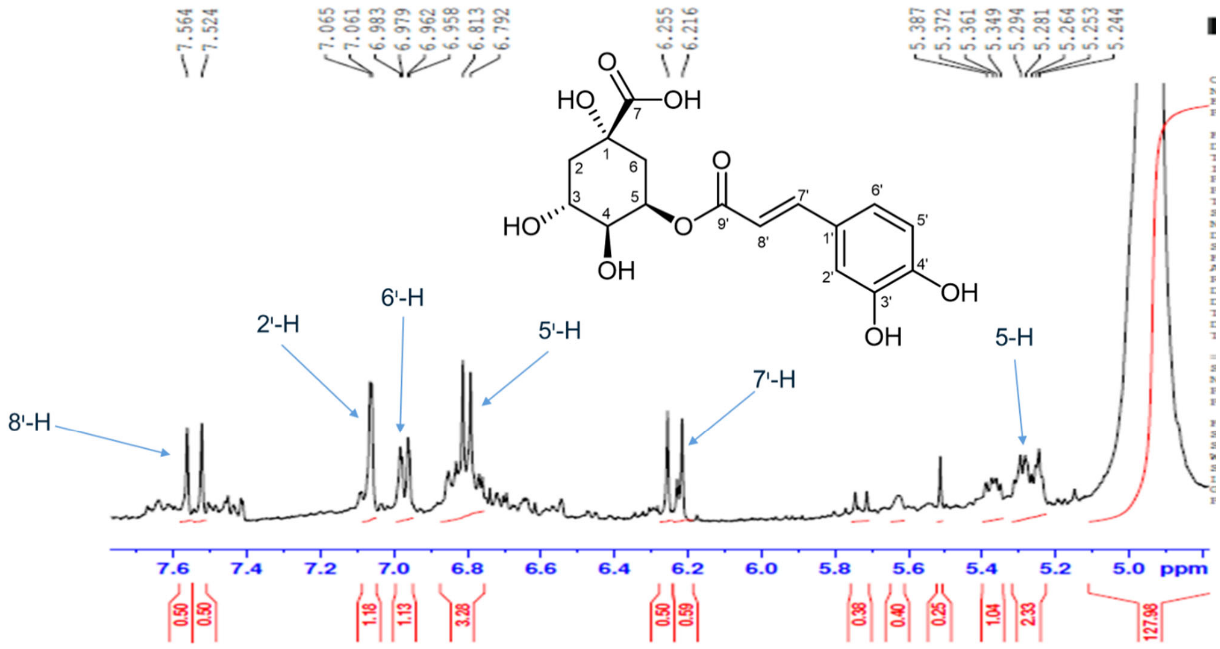

Figure S61. Expanded ^{1}H-NMR spectrum of 5-*O*-caffeoylquinic acid (**5**) [5].

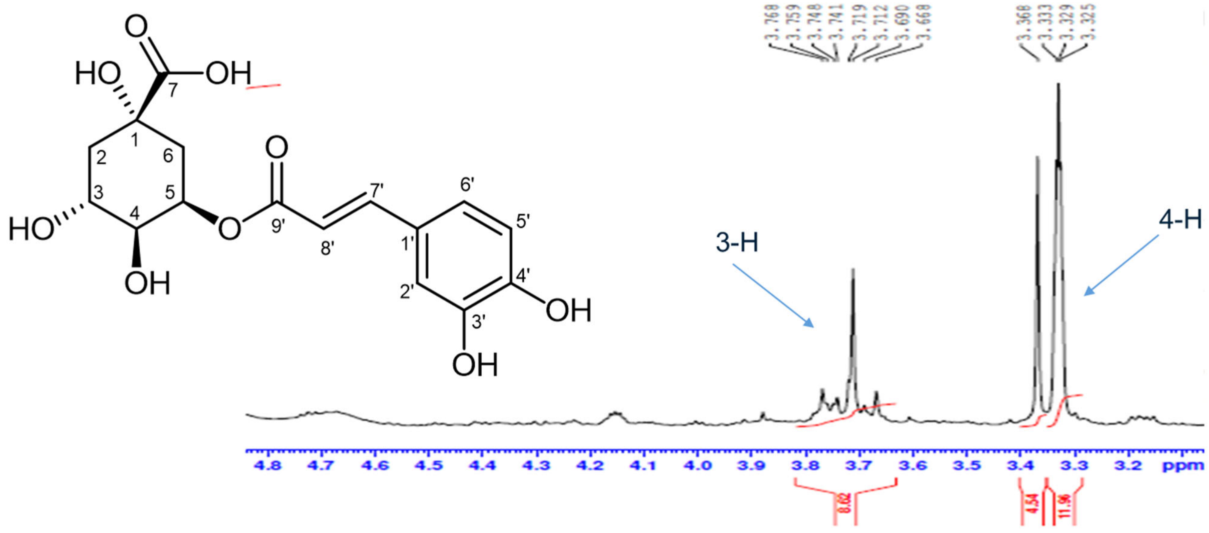

Figure S62. Expanded ^{1}H-NMR spectrum of 5-*O*-caffeoylquinic acid (**5**) [5].

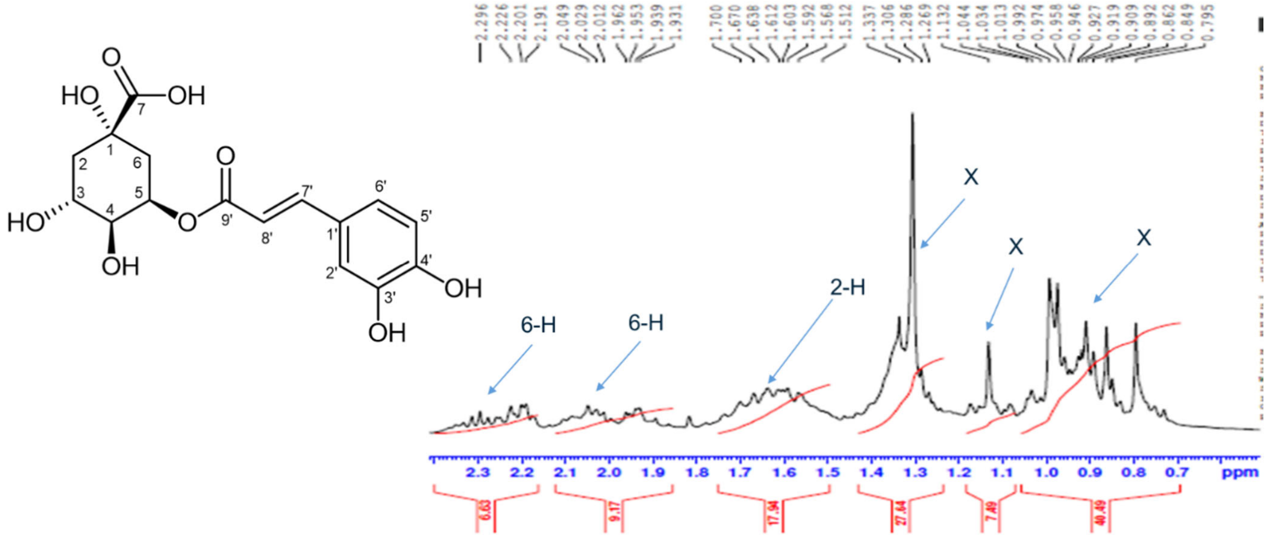

Figure S63. Expanded ^{1}H-NMR spectrum of 5-*O*-caffeoylquinic acid (**5**) [5].

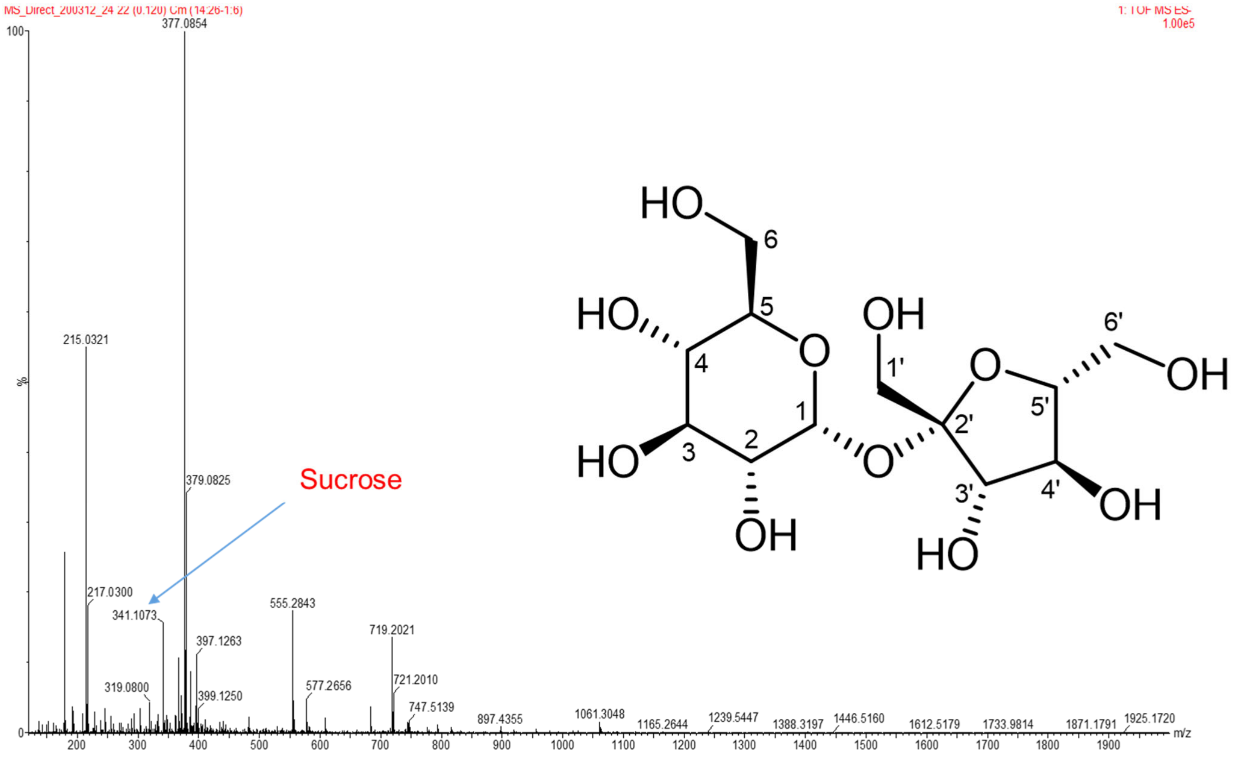

Figure S64. MS spectrum of sucrose (**6**) [6].

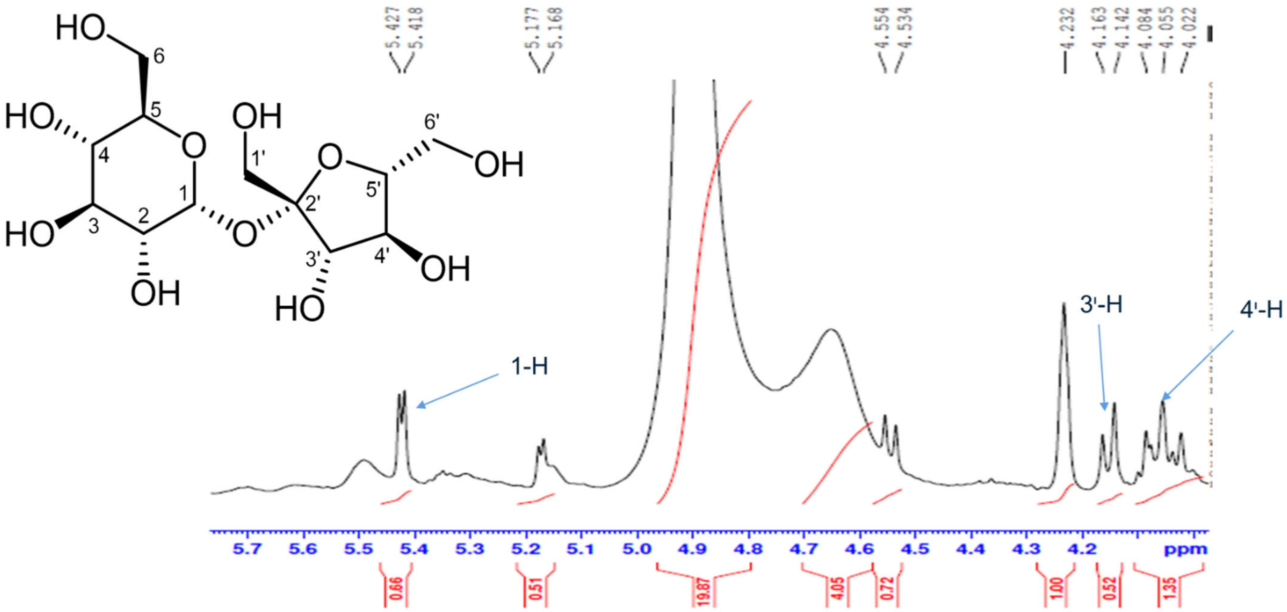

Figure S65. Expanded ^{1}H-NMR spectrum of sucrose (**6**) [6].

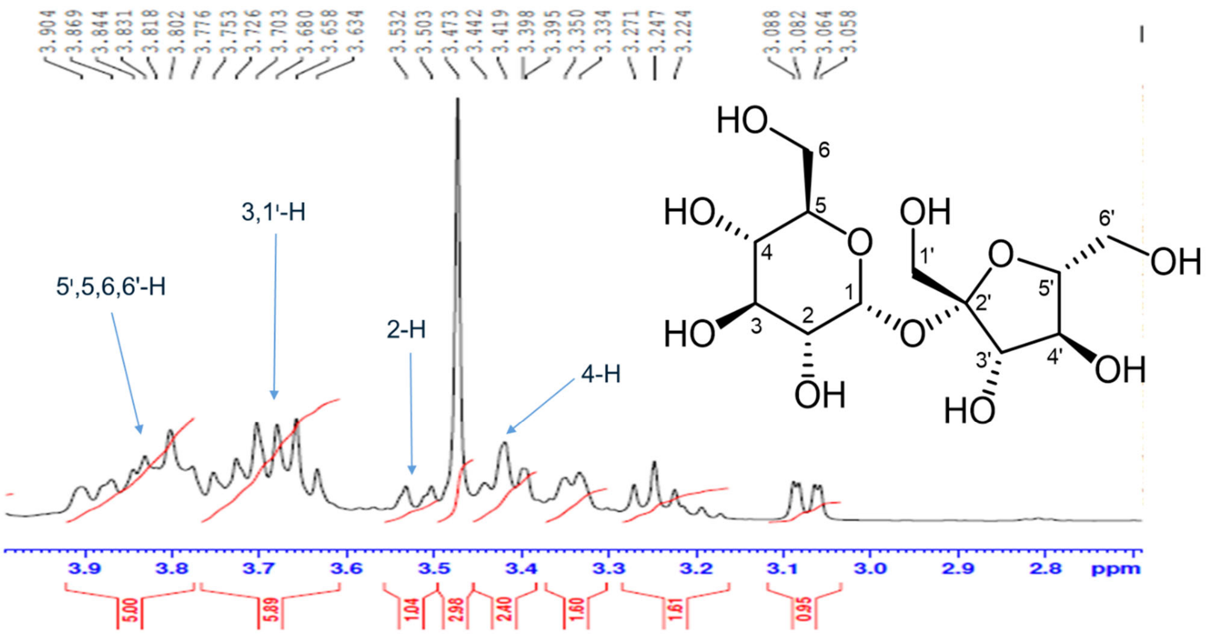

Figure S66. Expanded ^{1}H-NMR spectrum of sucrose (**6**) [6].

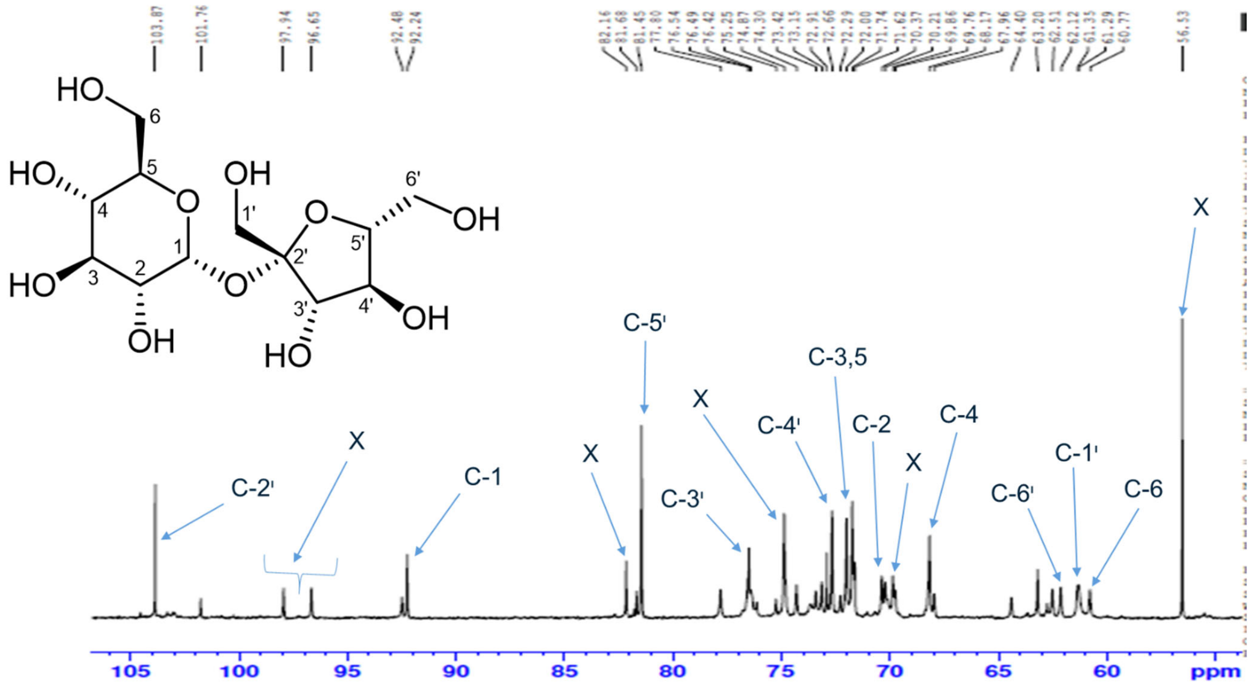

Figure S67. ^{13}C-NMR spectrum of sucrose (**6**) [6].

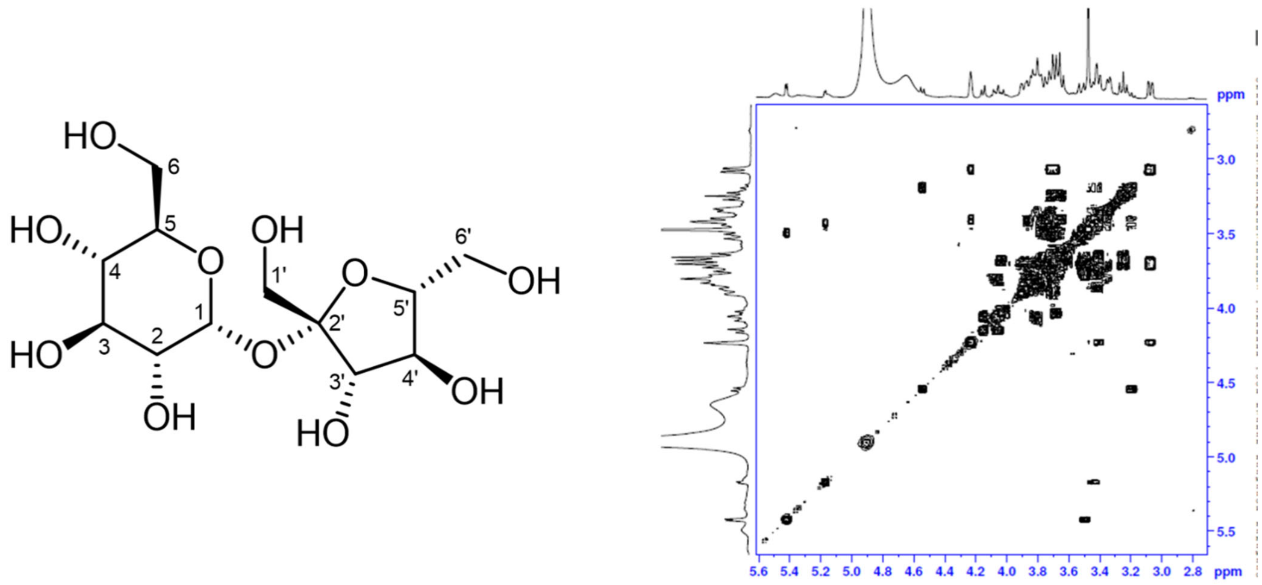

Figure S68. COSY spectrum of sucrose (**6**) [6].

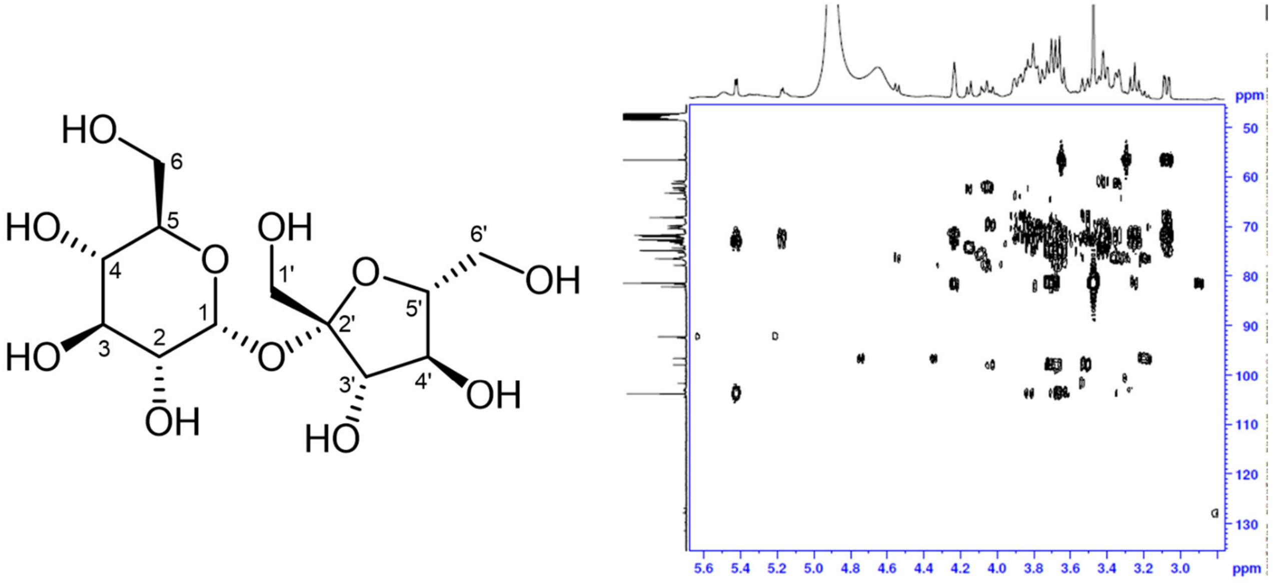

Figure S69. HMBC spectrum of sucrose (**6**) [6].

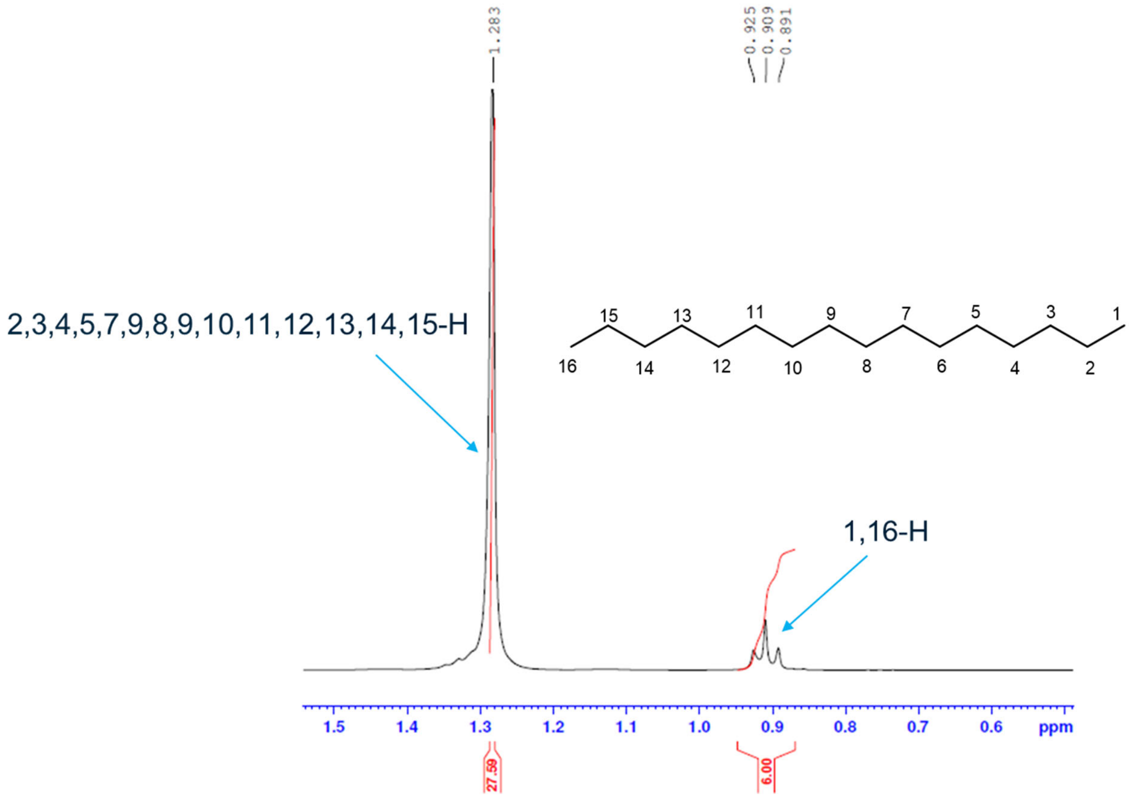

Figure S70. Expanded ^{1}H-NMR spectrum of hexadecane (**7**) [7].

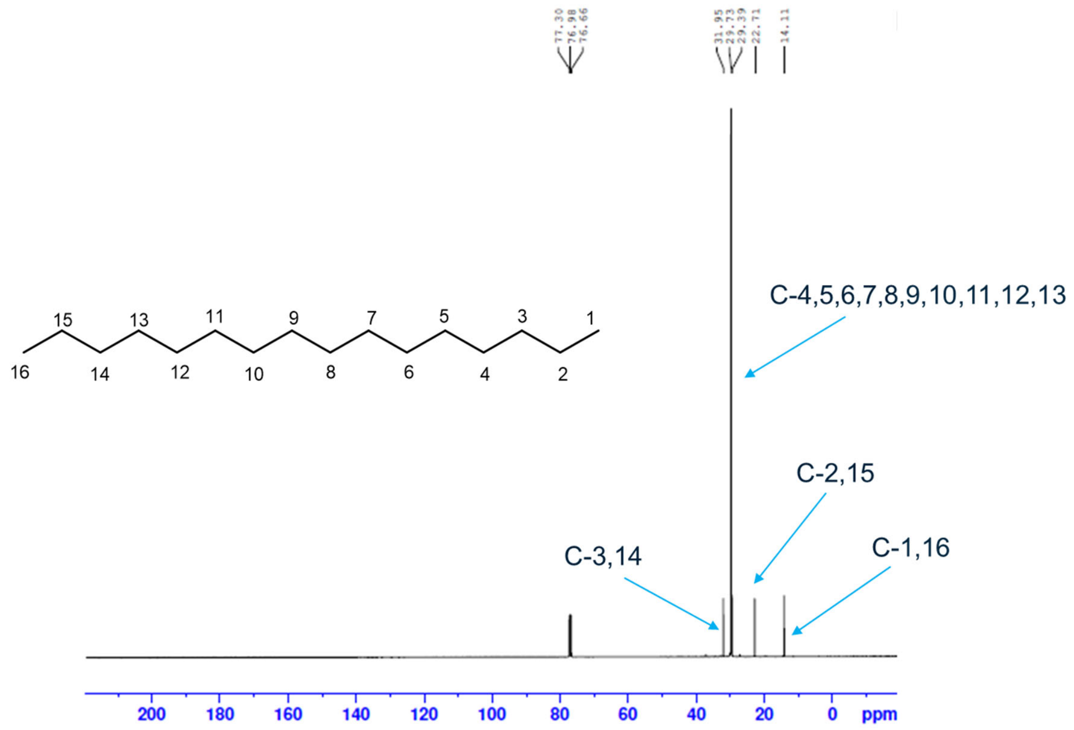

Figure S71. ^{13}C-NMR spectrum of hexadecane (**7**) [7].

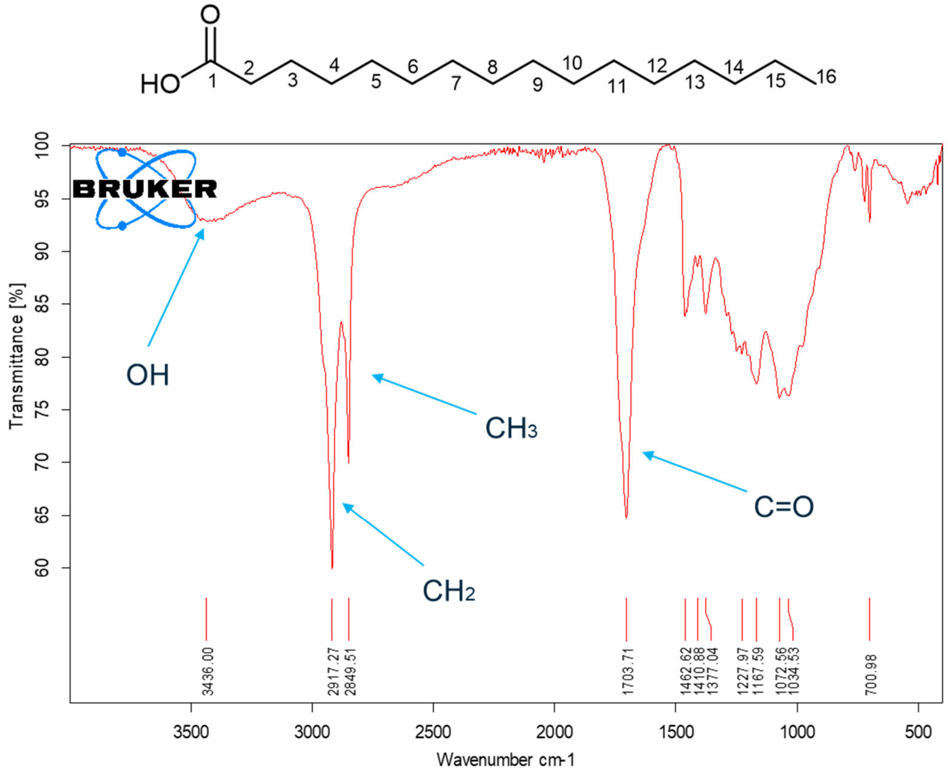

Figure S72. IR spectrum of palmitic acid (**8**) [8].

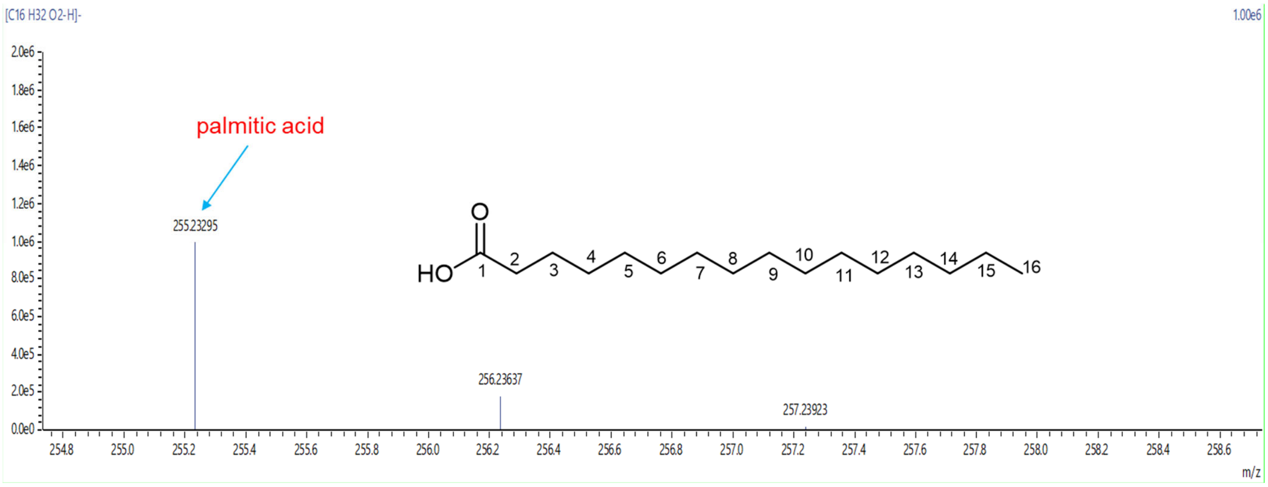

Figure S73. MS spectrum of palmitic acid (**8**) [8].

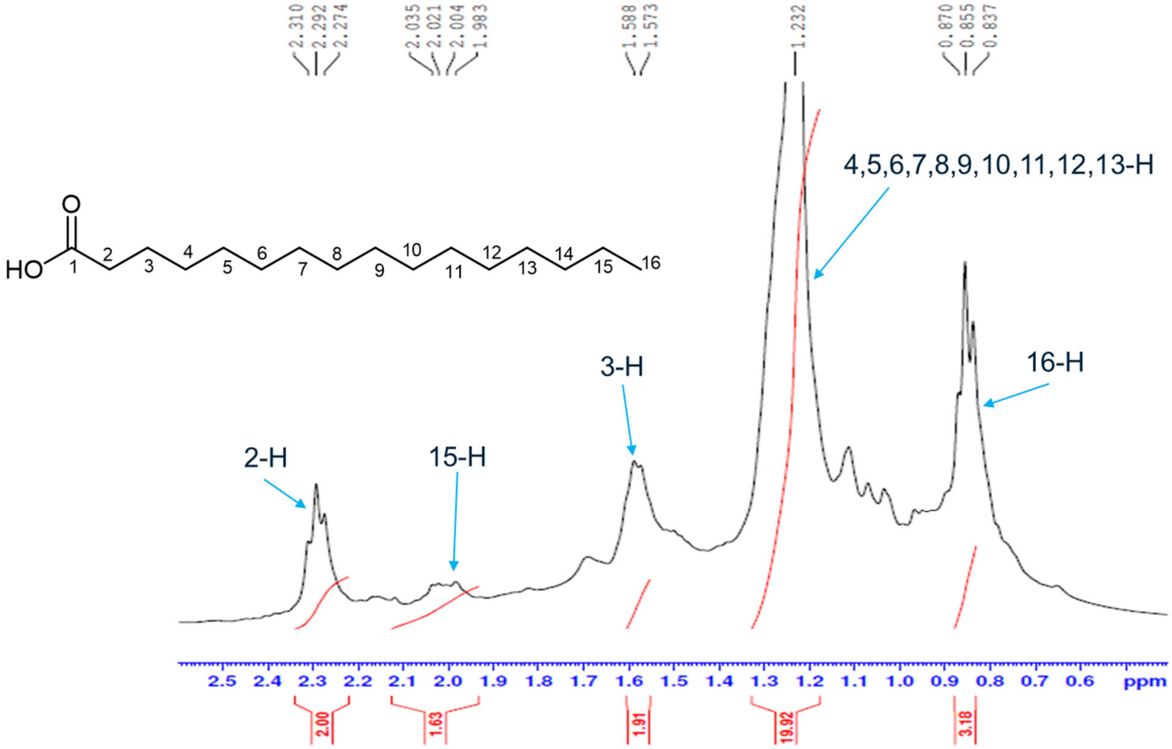

Figure S74. Expanded ¹H-NMR spectrum of palmitic acid (**8**) [8].

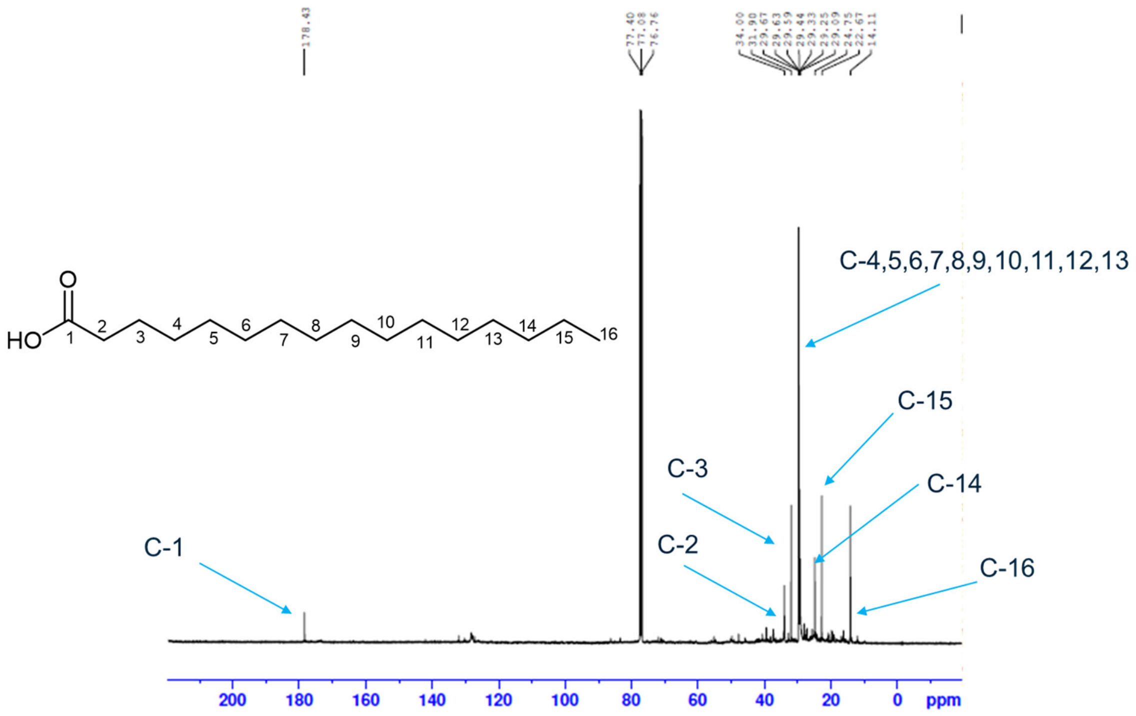

Figure S75. ^{13}C-NMR spectrum of palmitic acid (**8**) [8].

References

1. Yoshimura, M.; Ito, H.; Miyashita, K.; Hatano, T.; Taniguchi, S.; Amakura, Y.; Yoshida, T. Flavonol glucuronides and C-glucosidic ellagitannins from *Melaleuca squarrosa. Phytochemistry*, **2008**, *69*, 3062-3069.

2. Mouffok, S.; Haba, H.; Lavaud, C.; Long, C.; Benkhaled, M. Chemical constituents of Chemical constituents of *Centaurea omphalotricha Coss. & Durieu ex Batt. & Trab. Records of Natural Products*, **2012**, *6*, 292-5.

3. Ogawa, K.; Yamamura, M.; Maruyama, I. Isolation and identification of 3-*O*-methyl-d-galactose as a constituent of neutral polysaccharide of *Chlorella vulgaris. Bioscience, Biotechnology, and Biochemistry*, **1994**, *58*, 942-944.

4. Yang, L.; Zhang, L.; Du, J.; Shao, L.; Yu, F.; Li, R.; Zhong, J. Two new oleanane triterpenoid saponins from Elsholtzia bodinieri. *Natural Product Research*, **2020**, 1-9.

5. Suarez-Quiroz, M.L.; Campos, A.A.; Alfaro, G.V.; González-Ríos, O.; Villeneuve, P.; Figueroa-Espinoza, M.C. Isolation of green coffee chlorogenic acids using activated carbon. *Journal of Food Composition and Analysis*, **2014**, *33*, 55-58.

6. Ben Youssef, S.; Fakhfakh, J.; Tchoumtchoua, J.; Halabalaki, M.; Allouche, N. Efficient purification and complete NMR characterization of galactinol, sucrose, raffinose, and stachyose isolated from *Pinus halepensis* (Aleppo pine) seeds using acetylation procedure. *Journal of Carbohydrate Chemistry*, **2016**, *35*, 224-237.

7. Li, X.Y.; Shang, R.; Fu, M.C.; Fu, Y. Conversion of biomass-derived fatty acids and derivatives into hydrocarbons using a metal-free hydrodeoxygenation process. *Green Chemistry*, **2015**, *17*, 2790-2793.

8. Bulama, J.; Dangoggo, S.; Halilu, M.; Tsafe, A.I.; Hassan, S.W. Isolation and characterization of palmitic acid from ethyl acetate extract of root bark of *Terminalia glaucescens*. *Chemistry and Materials Research*, **2014**, *6*, 140-3.
